# Nuclear import receptors are recruited by FG-nucleoporins to rescue hallmarks of TDP-43 proteinopathy

**DOI:** 10.1186/s13024-022-00585-1

**Published:** 2022-12-08

**Authors:** Bilal Khalil, Deepak Chhangani, Melissa C. Wren, Courtney L. Smith, Jannifer H. Lee, Xingli Li, Christian Puttinger, Chih-Wei Tsai, Gael Fortin, Dmytro Morderer, Junli Gao, Feilin Liu, Chun Kim Lim, Jingjiao Chen, Ching-Chieh Chou, Cara L. Croft, Amanda M. Gleixner, Christopher J. Donnelly, Todd E. Golde, Leonard Petrucelli, Björn Oskarsson, Dennis W. Dickson, Ke Zhang, James Shorter, Shige H. Yoshimura, Sami J. Barmada, Diego E. Rincon-Limas, Wilfried Rossoll

**Affiliations:** 1 Department of Neuroscience, Mayo Clinic, Jacksonville, FL 32224 USA; 2 Department of Neurology, McKnight Brain Institute, Norman Fixel Institute for Neurological Diseases, University of Florida, Gainesville, FL 32610 USA; 3 Mayo Clinic Graduate School of Biomedical Sciences, Neuroscience Track, Mayo Clinic, Jacksonville, FL USA; 4 Department of Neurology, University of Michigan, Ann Arbor, MI 48109 USA; 5 Graduate School of Biostudies, Kyoto University, Yoshida-konoe, Sakyo-ku, Kyoto, Japan; 6 Geriatric Department, Affiliated Hospital of Qingdao University, Qingdao, Shandong China; 7 Department of Biology, Stanford University, Stanford, CA 94305 USA; 8 Department of Neuroscience, Center for Translational Research in Neurodegenerative Disease, University of Florida, Gainesville, FL 32610 USA; 9 UK Dementia Research Institute at University College London, London, UK; 10 Department of Neurobiology, University of Pittsburgh School of Medicine, Pittsburgh, PA 15213 USA; 11 LiveLikeLou Center for ALS Research, University of Pittsburgh Brain Institute, Pittsburgh, PA 15261 USA; 12 Department of Neurology, Mayo Clinic, Jacksonville, FL USA; 13 Department of Biochemistry and Biophysics, Perelman School of Medicine, University of Pennsylvania, Philadelphia, PA 19104 USA; 14 Genetics Institute, University of Florida, Gainesville, FL 32610 USA

**Keywords:** Amyotrophic lateral sclerosis, Frontotemporal dementia, TDP-43, Nucleocytoplasmic transport, Importin, Nuclear pore, Aggregation, Prion-like domain, Drosophila

## Abstract

**Background:**

Cytoplasmic mislocalization and aggregation of TAR DNA-binding protein-43 (TDP-43) is a hallmark of the amyotrophic lateral sclerosis and frontotemporal dementia (ALS/FTD) disease spectrum, causing both nuclear loss-of-function and cytoplasmic toxic gain-of-function phenotypes. While TDP-43 proteinopathy has been associated with defects in nucleocytoplasmic transport, this process is still poorly understood. Here we study the role of karyopherin-β1 (KPNB1) and other nuclear import receptors in regulating TDP-43 pathology.

**Methods:**

We used immunostaining, immunoprecipitation, biochemical and toxicity assays in cell lines, primary neuron and organotypic mouse brain slice cultures, to determine the impact of KPNB1 on the solubility, localization, and toxicity of pathological TDP-43 constructs. Postmortem patient brain and spinal cord tissue was stained to assess KPNB1 colocalization with TDP-43 inclusions. Turbidity assays were employed to study the dissolution and prevention of aggregation of recombinant TDP-43 fibrils in vitro. Fly models of TDP-43 proteinopathy were used to determine the effect of KPNB1 on their neurodegenerative phenotype in vivo.

**Results:**

We discovered that several members of the nuclear import receptor protein family can reduce the formation of pathological TDP-43 aggregates. Using KPNB1 as a model, we found that its activity depends on the prion-like C-terminal region of TDP-43, which mediates the co-aggregation with phenylalanine and glycine-rich nucleoporins (FG-Nups) such as Nup62. KPNB1 is recruited into these co-aggregates where it acts as a molecular chaperone that reverses aberrant phase transition of Nup62 and TDP-43. These findings are supported by the discovery that Nup62 and KPNB1 are also sequestered into pathological TDP-43 aggregates in ALS/FTD postmortem CNS tissue, and by the identification of the fly ortholog of KPNB1 as a strong protective modifier in *Drosophila* models of TDP-43 proteinopathy. Our results show that KPNB1 can rescue all hallmarks of TDP-43 pathology, by restoring its solubility and nuclear localization, and reducing neurodegeneration in cellular and animal models of ALS/FTD.

**Conclusion:**

Our findings suggest a novel NLS-independent mechanism where, analogous to its canonical role in dissolving the diffusion barrier formed by FG-Nups in the nuclear pore, KPNB1 is recruited into TDP-43/FG-Nup co-aggregates present in TDP-43 proteinopathies and therapeutically reverses their deleterious phase transition and mislocalization, mitigating neurodegeneration.

**Graphical Abstract:**

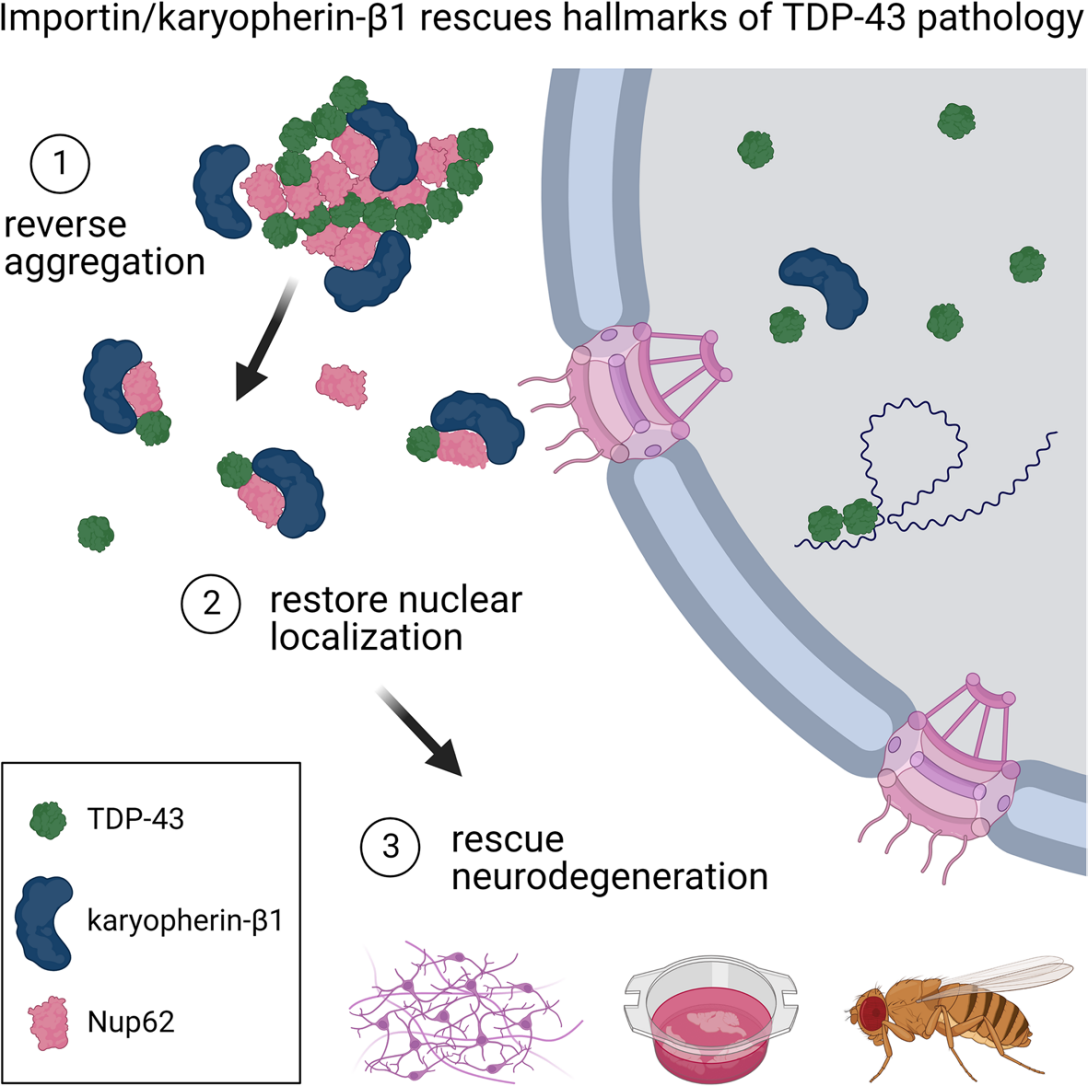

**Supplementary Information:**

The online version contains supplementary material available at 10.1186/s13024-022-00585-1.

## Background

TDP-43 pathology is characterized by the mislocalization of TDP-43 protein from the nucleus into cytoplasmic deposits where it undergoes hyperphosphorylation, ubiquitination and proteolytic fragmentation [1–3]. It is the major neuropathological hallmark in ~ 97% of ALS and ~ 50% of FTD cases, two progressive and devastating neurodegenerative disorders with overlapping genetic, clinical, and histopathological features [4], but is also present in other neurodegenerative disorders including Alzheimer’s disease (AD) and its mimic limbic-predominant age-related TDP-43 encephalopathy (LATE) [5, 6]. TDP-43 is a predominantly nuclear regulator of mRNA splicing and translation that is also found in cytoplasmic stress granules and axonal mRNA transport granules [7–9]. Its mislocalization causes both nuclear loss-of-function and cytoplasmic toxic gain-of-function defects that contribute to neurodegeneration [10]. While nuclear depletion of TDP-43 leads to mis-splicing events and cryptic exon inclusion in key neuronal genes including *STMN2* (stathmin-2) [11, 12] and *UNC13A* [13, 14], cytoplasmic TDP-43 aggregates cause defects in different cellular pathways such as autophagy, endocytosis and nucleocytoplasmic transport (NCT) [3, 15].

Previously, we demonstrated that pathological TDP-43 can affect the distribution and function of nuclear pore complexes (NPC) by recruiting nucleoporins (Nups) into cytoplasmic aggregates in model systems and patient brain tissue [16]. Here, we show that several nuclear import receptors (NIRs) can increase the solubility of TDP-43 in vitro. We investigated how the NIR karyopherin-β1 (KPNB1), which mediates the nuclear import of TDP-43 via its classical nuclear localization signal (NLS) [17], affects TDP-43 pathology across various in vitro and in vivo models. We demonstrate that expression of KPNB1 rescues the mislocalization, aggregation and neurotoxicity of pathological TDP-43 across multiple disease models in cells and mouse brain tissue in an NLS-independent manner. These findings are further supported by our identification of the *Drosophila* KPNB1 ortholog as a robust modifier of human TDP-43-induced toxicity in a genetic screen. We discovered that KPNB1 activity towards pathological TDP-43 aggregates depends on its prion-like domain (PrLD), and that this novel mechanism is mediated by FG-rich Nups, such as Nup62, which co-aggregate with TDP-43 in vitro and in human autopsy brain and spinal cord tissue. This mechanism differs from previous studies showing that the NIR transportin-1 (TNPO1/KPNB2) can reverse pathological phase transition of FUS, by binding its PY-type NLS motif [18–21]. The FUS NLS and TDP-43 PrLD are also the domains where the majority of ALS-causing mutations are clustered [22]. Based on our findings, we propose a new model where KPNB1, related to its canonical role in dissociating FG-Nups in the nuclear pore, is recruited into cytoplasmic TDP-43/FG-Nup co-aggregates where it can reverse all hallmarks of TDP-43 pathology by restoring protein solubility and localization and reducing TDP-43-dependent neurodegeneration.

## Methods

### DNA constructs

The expression plasmids used in this study were obtained from multiple sources or generated as summarized in Supplementary Table 3. A flexible linker [(GGGS)_3_] was inserted between fluorescent fusion proteins to ensure proper protein folding.

### Mammalian cell culture and transfection

HEK293T cells were cultured in DMEM media (Life Technologies) containing 10% FBS. Human SH-SY5Y neuroblastoma cells were cultured in DMEM/F12 media (Life Technologies) containing 10% FBS. Cells were transfected with NeuroMag Neo (OZ Biosciences) according to the manufacturer’s protocol. HEK293T and SH-SY5Y cells were purchased from ATCC (CRL-11268 and CRL-2266, respectively). HEK293T cells were selected for certain experiments due to their robust protein aggregation and resistance to TDP-43 toxicity compared to SH-SY5Y neuroblastoma cells and primary neurons.

### Immunostaining and image acquisition and analysis

Cells were plated on coverslips in 12-well plates. Twenty-four hours after transfection, cells were fixed with 4% paraformaldehyde (PFA) in PBS for 15 min at room temperature (RT). For experiments with GFP-(GR)_100_, cells were fixed 48 h after transfection. Cells were permeabilized with 0.2% Triton X-100 in PBS for 10 min, blocked with 5% bovine serum albumin (BSA) for 45 min and incubated with the following primary antibodies: anti-FLAG (1:500), anti-TDP-43 (1:500), anti-phospho-TDP-43^S409/410^ (1:2000), anti-Nup62 (1:250), anti-Nup98 (1:250), anti-Ran (1:200) and anti-KPNB1 (1:100) overnight at 4 °C, followed by incubation with fluorophore-coupled secondary antibodies for 1 h at RT.

For high-resolution fluorescence microscopy, single and multiple z-plane images were acquired using an epifluorescence microscope (Nikon Eclipse Ti2) equipped with a Zyla camera (Zyla sCMOS, Andor). Within each experiment, all groups were imaged with the same acquisition settings. Image stacks were deconvolved using a 3D blind constrained iterative algorithm (Nikon NIS Elements, version 5.30.03).

To quantitate the spatial overlap between Nup62-mCherry and GFP-tagged TDP-43 constructs, Mander’s colocalization coefficient was calculated using the JACoP plugin on ImageJ [23]. Values close to 0 and 1 indicate weak and perfect colocalization, respectively.

As a reporter for nucleocytoplasmic transport of proteins, NES-tdTomato-NLS was co-transfected with GFP or GFP-tagged KPNB1 constructs in SH-SY5Y cells. The mean pixel intensity of NES-tdTomato-NLS in the nucleus and cytoplasm was measured to calculate the nuclear-to-cytoplasmic (N-to-C) ratio.

To semi-quantitatively determine nuclear-cytoplasmic localization of GFP-tagged TDP-43^mNLS^, HEK293T cells were co-transfected with GFP-TDP-43^mNLS^ and different mCherry-KPNB1 constructs, fixed, DNA-stained with Hoechst and imaged. Z-stacks were acquired as described above. Images were blinded across conditions and TDP-43^mNLS^ fluorescent signal was characterized as nuclear/nucleocytoplasmic/cytoplasmic for at least 20 cells per condition, in triplicate.

### Cell lysis, subcellular fractionation and immunoblotting

Cells were seeded in 12-well plates. Forty-eight hours after transfection, cells were lysed in RIPA Lysis and Extraction buffer (Thermo Fisher Scientific) supplemented with a protease inhibitor cocktail (Thermo Fisher Scientific) and centrifuged at 15000 rpm for 20 min at 4 °C. The supernatant was collected as the detergent-soluble fraction and the insoluble pellet was recovered in urea buffer (7 M urea, 2 M thiourea, 4% CHAPS, 50 mM Tris pH 8, complete protease inhibitor cocktail). For experiments with total protein lysate, cells were collected in urea buffer. The samples were left at RT for 20 min then briefly sonicated and centrifuged at 15000 rpm for 20 min. The supernatant was collected as the detergent-insoluble fraction. For immunoblotting, the samples were boiled in Laemmli sample buffer for 5 min at 98 °C and separated in Bolt™ 4 to 12% Bis-Tris gels (Thermo Fisher Scientific). Proteins were blotted onto nitrocellulose membranes using an iBlot™ 2 Gel Transfer device (Thermo Fisher Scientific). Membranes were blocked with Odyssey blocking buffer (LI-COR) for 1 h followed by incubation with the following primary antibodies: anti-mCherry (1:1000), anti-GFP (1:2000), anti-β-tubulin (1:1000), anti-β-actin (1:1000) and anti-KPNB1 (1:1000) overnight at 4 °C and incubation with secondary antibodies (1:10,000) in PBS blocking buffer with 0.05% Tween 20 for 1 h at RT. Blots were imaged using an Odyssey scanner (LI-COR) and Image Studio software (version 5.2, LI-COR), quantification of bands intensity was performed using Image Studio Lite (version 5.2, LI-COR).

### Immunoprecipitation (IP)

HEK293T cells were seeded in 6-well plates. Forty-eight hours after transfection, cells were lysed in IP Lysis buffer (Thermo Fisher Scientific) supplemented with a protease inhibitor cocktail (Thermo Fisher Scientific) and centrifuged at 15000 rpm for 20 min at 4 °C. A small aliquot of the recovered supernatant was collected as input extract, and the rest was used for IP. Pull-down was performed using GFP- and RFP-Trap magnetic beads (Chromotek) according to the manufacturer’s protocol. Briefly, beads were washed twice in wash buffer (10 mM Tris/Cl pH 7.5, 150 mM NaCl, 0.5 mM EDTA). The lysate was then incubated with the equilibrated beads overnight at 4 °C with end-over-end rotation. Beads were magnetically separated from the supernatant (flow-through) and washed 3 times with wash buffer. Bound proteins were released from the beads in an elution buffer (200 mM glycine pH 2.5). Samples were boiled in Laemmli buffer for 10 min at 95 °C and used for immunoblotting. The following primary antibodies were used: anti-mCherry (1:1000), anti-GFP (1:2000), anti-mAb414 (1:1000), anti-Nup62 (1:1000), anti-Nup50 (1:1000), anti-importin-α1 (1:1000) and anti-Ran (1:2000).

### RNA isolation and real-time RT-PCR

RNA from cultured HEK293T cells was isolated using RNeasy Plus Mini Kit (Qiagen) according to the manufacturer’s protocol. Isolated RNA was treated with RQ1 DNase (Promega) and used for the cDNA synthesis with SuperScript First-Strand Synthesis System (Invitrogen) using oligo-dT and random hexamer primers. Real-time RT-PCR was performed using TaqMan Universal PCR Master Mix (Applied Biosystems) and TaqMan gene expression assays for *EGFP* (Mr04097229_mr, Thermo Fisher Scientific) and human *GAPDH* (Hs02758991_g1, Thermo Fisher Scientific) on QuantStudio 7 Flex Real-Time PCR system (Applied Biosystems) with QuantStudio Real-Time PCR software (version 1.1, Thermo Fisher Scientific). Data was analyzed using the Design and Analysis software (version 2.6.0, Thermo Fisher Scientific).

### Cell death assay

Cell death of SH-SY5Y cells was assessed by the uptake of the membrane-impermeant Live-or-Dye™ Fixable Dead Cell Dye 640/662 (Live-or-Dye™ Fixable Viability Staining Kit, 32,007, Biotium). The dye penetrates dying cells that have compromised membrane integrity and labels amines on intracellular proteins. Cells were transfected with mCherry/mCherry-TDP-CTF and GFP/GFP-tagged KPNB1 constructs using FuGene6 (Promega). Forty-eight hours after transfection, cells were incubated with the dye for 30 min at 37 °C protected from light, then fixed in 4% PFA and imaged using an epifluorescence microscope (Nikon Eclipse Ti2). Cell death was assessed by scoring the proportion of dying cells that incorporated the dye.

### Recombinant protein purification

Hexahistidine (His_6_)-tagged proteins (TDP-CTF and Nup62FG) were purified as described previously [24]. *E. coli* cells (BL21-CodonPlus(DE3)-RIL, Agilent Technologies) harbouring the expression vector for His_6_-tagged IDRs were cultured in Luria–Bertani medium. Protein expression was induced by adding 1 mM IPTG, and the cultures were further incubated at 18 °C overnight. Cells were harvested by centrifugation (5000 *g*, 15 min, 4 °C), subjected to two rounds of freeze-thaw cycles and finally dissolved in urea-containing buffer (8 M urea, 10 mM Tris-HCl, 100 mM NaH_2_PO_4_, 5 mM 2-mercaptoethanol, 10 mM imidazole, pH 8.0) at 4 °C overnight for 2 days. The lysate was centrifuged (10,000 *g*, 4 °C, 30 min) and the supernatant was collected and mixed with Ni-NTA agarose beads (Qiagen). The beads were gently agitated at 4 °C for 1 h, washed with wash buffer (8 M urea, 10 mM Tris-HCl, 100 mM NaH_2_PO_4_, 10 mM 2-mercaptoethanol, 20 mM imidazole, pH 8.0), and His_6_-tagged proteins were eluted with elution buffer (8 M urea, 10 mM Tris-HCl, 100 mM NaH_2_PO_4_, 5 mM 2-mercaptoethanol, and 100, 300, or 500 mM imidazole, pH 8.0). Eluted proteins were sequentially dialyzed at 4 °C in dialysis buffer 1 (0.1% (v/v) trifluoroacetic acid (TFA), 2 mM 2-mercaptoethanol) for 3 h, dialysis buffer 2 (0.05% (v/v) TFA, 2 mM 2-mercaptoethanol) for 3 h or overnight, and dialysis buffer 3 (0.05% (v/v) TFA) for 3 h. The purified proteins were lyophilized (FDU-2200, EYELA) and stored at 4 °C.

*E. coli* cells (BL21-CodonPlus (DE3)-RIL, Agilent Technologies) harbouring the expression vector for GST-tagged KPNB1 (full-length and HEAT1–9) were cultured in LB medium. Protein expression was induced by adding 0.5 mM IPTG, and the cultures were further incubated at 18 °C overnight. The cells were harvested by centrifugation (5000 *g*, 15 min, 4 °C) and stored at − 80 °C. The cell pellet was dissolved in lysis buffer (50 mM Tris-HCl, 500 mM NaCl, 1 mM MgCl_2_, 2 mM 2-mercaptoethanol, 1 mM phenylmethylsulfonyl fluoride, 200 μg/mL lysozyme, 20 μg/mL DNaseI, pH 7.4) and subjected to three rounds of quick freeze-thaw cycles. The cell debris were removed by centrifugation (12,000 *g*, 4 °C, 20 min) and the supernatant was mixed with Glutathione-Sepharose beads (Cytiva) at 4 °C for 1 h. The beads were then washed with wash buffer (50 mM Tris-HCl, 500 mM NaCl, 2 mM 2-mercaptoethanol, pH 7.4) three times. KPNB1 protein were separated from the beads by protease digestion. The protein-bound beads were incubated with PreScission Protease (Cytiva) in Digestion Buffer (50 mM, 100 mM NaCl, 1 mM DTT) at 4 °C for 14 h. Protein was concentrated using Amicon centrifugal filters (Millipore) and stored at − 80 °C.

### In vitro protein dissolution and prevention assays

Lyophilized protein (His_6_-TDP-CTF and His_6_-Nup62FG) was dissolved in dissolving buffer (2 M guanidine hydrochloride, 100 mM Tris-HCl, pH 8.0, 10 mM HEPES) to a final concentration of 4 mM. Protein was incubated with 10 μM ATTO488/610-maleimide (ATTO-TEC) at RT for 1 h, and then with 5 mM dithiothreitol for 1 h. The labelled protein solution was diluted by a buffer (50 mM HEPES, 100 mM NaCl, pH 7.4) to a final concentration of 10 μM and incubated at RT for 30 min to allow aggregation. For dissolution assays, purified KPNB1 or BSA was added to pre-formed aggregates to a final concentration of 20 μM and incubated for 24 h. For aggregation prevention assays, TDP-CTF, Nup62FG and KPNB1 constructs were mixed at RT for 30 min. The mixture was then transferred to a 96-well clear-bottom plate (Greiner Bio-one) for microscopic observation (FV3000, Olympus). To measure turbidity, the mixture was transferred to a microcuvette and the optical density at 395 nm was measured using a V-630 spectrophotometer (JASCO).

### *Drosophila* genetics and behavioral assays

The stocks Gmr-Gal4 (BDSC:1104, all eye cells), Elav GeneSwitch (GS)-Gal4 (BDSC:43642, conditional pan-neural), control UAS-LacZ (BDSC:8529 and 8530), Ketel RNAi lines (BDSC:31242, 41,845, 44,576 and 27,567), and EP-Ketel (BDSC:15967) were obtained from the Bloomington Drosophila Stock Center. The Ketel RNAi line 107,622 was obtained from the Vienna Drosophila Resource Center. To generate flies expressing UAS-V5-Ketel, a synthetic DNA fragment carrying the Ketel coding sequence fused to the V5 tag at the N-terminal end was cloned into pJFRC-MUH via NotI and XbaI linkers, subsequently microinjected into fly embryos (Rainbow Transgenics, Inc.), and targeted to the attP2 landing site in the third chromosome. The UAS lines expressing human TDP-43^WT^, TDP-43^M337V^ and TDP-43^ΔNLS^ were previously described [25]. For eye experiments, females carrying the Gmr-Gal4 driver recombined with TDP-43 transgenes were crossed with males from all the aforesaid Ketel stocks at 26.5 °C. Then, 1-day old flies were collected after eclosion for eye imaging [26] and phenotype quantification using the Flynotyper tool and severity scores as described [25, 27]. For conditional expression in the fly CNS, females carrying UAS-TDP-43^M337V^ and ElavGS-Gal4 transgenes were crossed with males expressing the control UAS-LacZ reporter along with UAS-V5-Ketel and UAS-Ketel RNAi transgenes at 25 °C. After eclosion, adults were kept in the presence or absence of RU486 (40 μg/mL) and aged depending on the assay. For whole-mount immunofluorescence of pTDP-43, brains from 2-day old fly males were dissected in cold PBS, fixed in 4% formaldehyde, and stained with primary rabbit anti-human phospho-TDP-43^S409/410^ (1:300) and secondary goat anti-rabbit Alexa Fluor 594 (1:100) antibodies. In addition, fly heads were also used to quantify the levels of total TDP-43 protein by western blot as described [25]. For survival assays, 80 adult males from each genotype were distributed in five vials containing JazzMix fly food (Thermo Fisher Scientific) supplemented with RU486 or solvent (ethanol) and transferred every 2 days to fresh vials until the end of the experiment. The number of dead flies was recorded daily, and survival curves were plotted using GraphPad Prism 9.2.0 (332). The statistical analysis for survival was performed using the OASIS 2 online tool [28]. For locomotion analysis, 13 males were individually placed in tubes containing 5% sucrose and 2% agar with RU486 or solvent and incubated horizontally in DAM2 Drosophila activity monitor at 25 °C as described [29]. Flies were transferred to fresh food twice a week, and the activity was monitored every minute and summed up to Daily Activity for 14 days.

### Co-immunofluorescence on human post-mortem tissue

Well-characterized human post-mortem tissue cases were provided by the Mayo Clinic Florida brain bank. Tissue derived from spinal cord and hippocampus were obtained from patients with ALS or FTLD, respectively, in addition to primary motor cortex, and tissue from all three regions from neuropathologically normal control tissue blocks. The neuropathological curation of cases consists of sporadic and familial disease, where select cases were available for patients who developed ALS or FTLD due to genetic mutations in *C9orf72*, *SOD1*, or *TARDBP*. Post-mortem case demographics are provided in Supplementary Table 1. Informed consent was obtained for patient tissue donated for research studies and samples were obtained with approval from the Mayo Clinic Institutional Review Board. Brains and spinal cords were processed in 10% neutral buffered formalin before tissue blocks were selected during gross neuroanatomically examination and embedded in paraffin for their preservation and sectioning. Double immunofluorescence staining was performed on 5 μm formalin fixed paraffin embedded (FFPE) tissue sections obtained from the spinal cord, hippocampus, and primary motor cortex. Briefly, FFPE tissue sections were deparaffinized by immersion in xylene and rehydrated through a series of graded ethanol solutions (100, 90 and 70% ethanol). Tissue was rinsed in dH_2_O and antigen retrieval was performed by steaming tissue sections in citrate buffer, pH 6 (Dako Target Retrieval Solution, S2369) for 30 min. Tissue sections were cooled to RT and rinsed with dH_2_O for 10 min. Sections were permeabilized and blocked with serum-free protein block containing 0.3% Triton-X 100 at RT for 1 h. Tissues were immunostained with antibodies against phospho-TDP-43^S409/410^ (1:200), Nup62 (1:100) or KPNB1 (1:2000), diluted in antibody diluent (Dako, S0809) and incubated overnight at 4 °C in a humidified chamber. Tissue sections were washed in tris buffered saline (TBS) containing 0.05% Tween-20 (TBS-T), three times for 10 min each. Alexa Fluor conjugated secondary antibodies (1:1000, F′(ab)anti-mouse 488 or anti-rat 555 or anti-rat 647, Thermo Fisher Scientific), were diluted in antibody diluent (Dako, S0809) and incubated for 2 h at RT. Tissue was washed with TBS-T and incubated with Hoechst 33342 (1:1000, Thermo Fisher Scientific) for 20 min at RT before rinsing with TBS. To quench autofluorescence, tissues were stained with 1x True Black lipofuscin autofluorescence quencher (Biotium, 23,007) for 30 sec and rinsed with dH_2_O for 5 min. Tissue sections were mounted with glass slides using ProLong Glass Antifade Mountant (Invitrogen). High-resolution imaging was conducted, and optical sections were acquired, according to the depth of the tissue to capture the full contour of the pTDP-43 aggregate. Z-series were obtained using an Eclipse Ti2 fluorescence microscope (Nikon) equipped with a Spectra X multi-LED light engine (Lumencor), single bandpass filter cubes for DAPI, EGFP/FITC and TRITC (Chroma), and a Zyla 4.2 PLUS sCMOS camera (Andor), using NIS Elements HC V5.30 software (Nikon). Immunofluorescent staining of all cases from distinct CNS regions was conducted at the same time and all pathological subgroups were imaged with the same acquisition settings. Out of focus light from each z-series was eliminated using the Clarify.ai module of the NIS-Elements Advanced Research (V5.30) deconvolution package. Clarified z-series are shown as optical projections and 3D volume rendered views to demonstrate colocalization of KPNB1 and Nup62 with pTDP-43 inclusions. Fluorescent tile images of the same tissue sections were captured using a 40x objective on an inverted fluorescent microscope (Keyence BZ-X800) and high-resolution image stitching was completed using the Keyence analyzer package (BZ-X series).

### Organotypic brain slice cultures, transduction and immunohistochemistry

All animal procedures were approved by the Institutional Animal Care and Use Committee (IACUC) at Mayo Clinic. Brain slice cultures (BSCs) were prepared from postnatal day 8–9 (P8–9) C57BL/6 mice as previously reported [30]. Pups were placed in a chamber with isoflurane and decapitated after confirmation with toe-pinch response. The hemi-brains were dissected to collect the cortex and hippocampus in sterile-filtered ice-cold dissection buffer containing Hank’s balanced salt solution (HBSS), calcium, magnesium, no phenol red (Thermo Fisher Scientific), 2 mM ascorbic acid (Sigma Aldrich), 39.4 μM ATP (Sigma Aldrich) and 1% (v/v) penicillin/streptomycin (Thermo Fisher Scientific). Each hemi-brain was placed on filter paper and cut into 350 μm slices using a McIllwain™ tissue chopper (Stoelting Co.). Slices were placed in 6-well tissue culture plates to contain three slices per semi-porous membrane insert per well (0.4 μm pore diameter, MilliporeSigma). BSCs were maintained at 37 °C and 5% CO_2_ in sterile-filtered culture medium containing Basal Medium Eagle (Thermo Fisher Scientific), 26.6 mM HEPES (pH 7.1, Thermo Fisher Scientific), 511 μM ascorbic acid, 1% (v/v) GlutaMAX (Thermo Fisher Scientific), 0.033% (v/v) insulin (Sigma Aldrich), 1% (v/v) penicillin/streptomycin (Thermo Fisher Scientific) and 25% (v/v) heat-inactivated horse serum (Sigma Aldrich). Culture medium was changed every 3–4 days.

For AAV transduction of BSCs, AAV plasmids were first transfected in HEK293T using Polyethylenimine Hydrochloride (Polysciences). 3–4 days after transfection, cell media was collected and centrifuged at 800 rcf for 5 min to recover the supernatant containing secreted AAVs. AAVs were applied directly into the culture medium on the first day of the BSC (DIV0).

For immunostaining of BSCs, slices were fixed 12–15 days after transduction (DIV12–15) with 4% PFA for 1 h at RT, permeabilized with 0.5% Triton X-100 overnight at 4 °C and blocked with 20% BSA for 4 h. BSCs were incubated with the following primary antibodies: anti-FLAG (1:500), anti-GFP (1:500) and anti-TDP-43 (1:500) in 5% BSA for 48 h at 4 °C, followed by incubation with fluorophore-coupled secondary antibodies for 4 h at RT. To stain the nuclei, slices were incubated with the Hoechst dye (1:5000) for 30 min at RT, then coverslipped with ProLong Glass Antifade Mountant (Thermo Fisher Scientific). Nuclear-cytoplasmic localization of TDP-43 was semi-quantitatively determined as described above for HEK293T cells.

### Statistical analysis

Statistical comparisons between experimental groups were performed using either two-sided Student’s *t*-test or analysis of variance (one- or two-way ANOVA followed by Bonferroni’s post hoc test) in GraphPad Prism software (version 9.2.0). Differences were considered statistically significant when *p* < 0.05. Data is shown as mean ± SEM.

## Results

### A subset of β-importin family nuclear import receptors (NIRs) reduces cytoplasmic aggregation of TDP-CTF

An unexplored serendipitous finding from our previous study was that while many components of the NCT machinery, including most Nups, co-aggregated with the aggregation-prone C-terminal fragment of TDP-43 (TDP-CTF) in the cytoplasm, KPNB1 expression appeared to reduce its aggregation [16]. TDP-43 contains an NLS, two RNA-recognition motifs (RRM1 and 2) and an intrinsically disordered C-terminal PrLD that mediates multivalent protein-protein interactions and harbors most of the disease-causing missense mutations described in ALS/FTD patients [3]. TDP-CTF is a 25-kDa C-terminal fragment of TDP-43 that lacks the NLS but contains parts of RRM2 and the complete PrLD and is a major component of insoluble cytoplasmic aggregates in cortical and hippocampal regions of ALS and FTLD-TDP patient brain tissue [31]. Its expression recapitulates histopathological features of TDP-43 pathology by forming detergent-insoluble inclusions that are positive for phospho-TDP-43^S409/410^ (pTDP-43), p62/SQSTM1, and ubiquitin, and induces high levels of toxicity in neuronal cell lines and primary neurons [32, 33]. Expression in SH-SY5Y human neuroblastoma cells was chosen as a cellular model for TDP-43 proteinopathy due to the lack of pronounced TDP-43 pathology in human ALS/FTD patient-derived iPSCs differentiated into neurons. KPNB1 reduces TDP-CTF cytoplasmic aggregation in SH-SY5Y cells, rendering its localization more nucleocytoplasmic (Supplementary Fig. 1a). In complementary biochemical fractionation experiments, KPNB1 expression significantly reduced the detergent-insoluble protein levels of TDP-CTF and variants carrying ALS-causing point mutations without affecting endogenous insoluble TDP-43 levels (Supplementary Fig. 1a,d). Expression of mCherry- or untagged-KPNB1 significantly reduced insoluble TDP-CTF protein levels, with very little effect on soluble TDP-CTF (Supplementary Fig. 1b). TDP-CTF and TDP-43^mNLS^ (full-length TDP-43 with mutant NLS) form pathological cytoplasmic aggregates that are positive for pTDP-43 but show diffuse localization with no pathological hyperphosphorylation in the presence of mCherry-KPNB1 (Supplementary Fig. 1c). In addition, both mCherry- and untagged-KPNB1 constructs significantly reduced total protein levels of TDP-CTF, but not full-length TDP-43, indicating that KPNB1 specifically targets insoluble TDP-CTF and reduces its aggregation in a tag-independent manner (Supplementary Fig. 1e). KPNB1 did not affect transcript levels of either TDP-CTF or TDP-43 (Supplementary Fig. 1f).

**Fig. 1:**
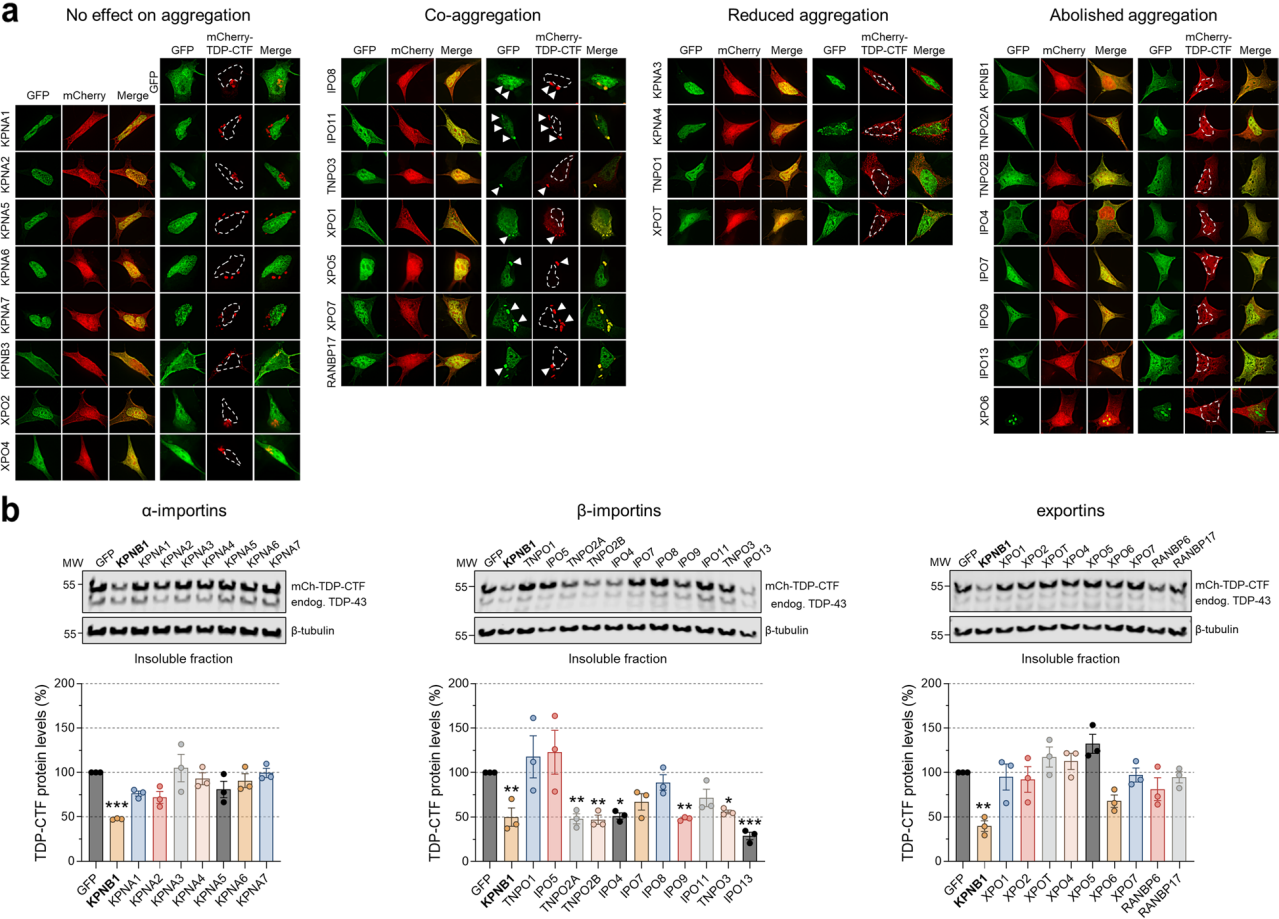
KPNB1 and other nuclear import receptors (NIRs) reduce the pathological aggregation of TDP-CTF. **a** Immunofluorescence of SH-SY5Y human neuroblastoma cells co-expressing mCherry or mCherry-TDP-CTF with GFP or one of 27 GFP-tagged α-importins, β-importins, and exportins. Based on this microscopy screen, transport proteins were divided in four categories according to their activity towards TDP-CTF aggregates: “no effect on aggregation”, “co-aggregation”, “reduced aggregation” (=transport proteins that only reduce the size of TDP-CTF aggregates), and “abolished aggregation” which includes most β-importins. Arrowheads indicate co-aggregation. Hoechst staining was used to outline nuclei. Scale bar: 5 μm. **b** Western blot analysis and quantification of insoluble mCherry-TDP-CTF protein levels in SH-SY5Y cells expressing GFP or one of each 27 GFP-tagged α-importins, β-importins, and exportins. Several β-importin family NIRs significantly reduced insoluble TDP-CTF levels, including KPNB1, IPO3/TNPO2, IPO4, IPO9, and IPO13. KPNB1 (in bold) was used as a reference. β-tubulin was used as a loading control. Statistical analysis was performed using one-way ANOVA and Bonferroni’s post hoc test (*p < 0.05, **p < 0.01, ***p < 0.001, *n* = 3)

Mammalian cells express several members of the α- and β-karyopherin families of nuclear transport receptors that share a similar curved α-solenoid structure composed of stacks of related armadillo or HEAT repeats, respectively [34]. To determine whether KPNB1 was the sole transport factor able to reduce TDP-43 pathology, we tested the effect of all 7 α-importin and 20 β-importin and exportin protein family members on TDP-CTF aggregates using fluorescence microscopy (Fig. 1a) and quantitative western blot analysis of insoluble proteins (Fig. 1b). Transport receptors were subdivided into four categories based on their activity towards TDP-CTF aggregates: “no effect on aggregation”, “co-aggregation”, “reduced aggregation” (including proteins that only reduced the size of individual TDP-CTF aggregates), and “abolished aggregation” (Fig. 1a). Notably, several β-importins, including IPO3/TNPO2, IPO4, IPO9, and IPO13, were able to reduce insoluble TDP-CTF levels similarly to KPNB1. TNPO1/KPNB2, which has previously been shown to disaggregate FUS [18–21], but not TDP-43 [18], did not decrease insoluble TDP-CTF protein levels but reduced the size of cytoplasmic TDP-CTF inclusions (Fig. 1a,b). No such effect was found for α-importins and exportins, with some exportins showing co-aggregation (Fig. 1a,b). These results suggest that while KPNB1 and other β-importins differ in their NLS recognition and substrate specificity for nuclear import, they share a common mechanism in reducing TDP-CTF aggregation.

### The N-terminal half of KPNB1 is required and sufficient to reduce TDP-CTF aggregation and toxicity

**Fig. 2:**
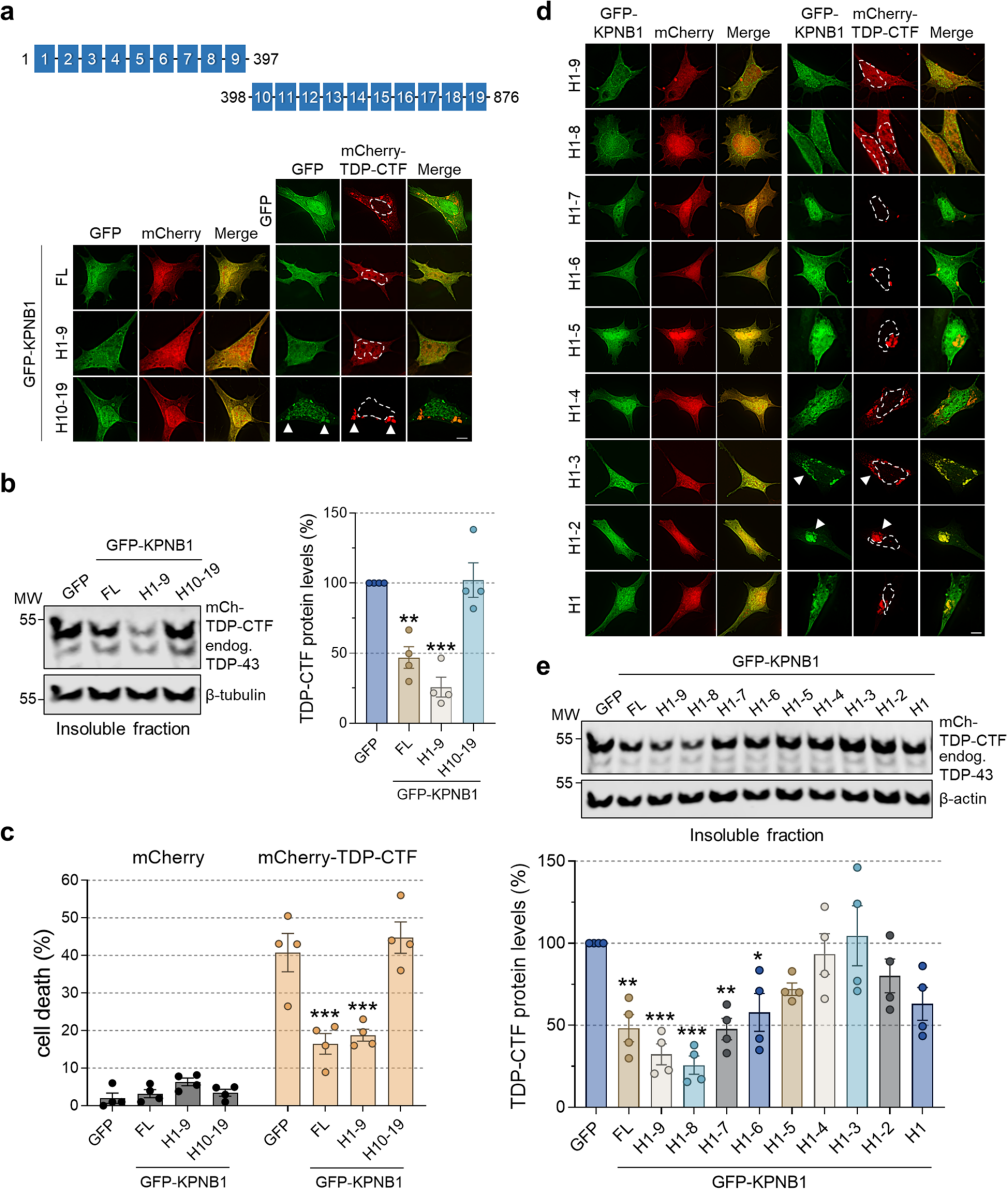
The N-terminal half of KPNB1 is required and sufficient to reduce TDP-CTF aggregation and toxicity. **a** (Top) Schematic domain structure of the N-terminal half (HEAT repeats 1–9, H1–9) and C-terminal half (HEAT repeats 10–19, H10–19) of KPNB1. (Bottom) Immunofluorescence (IF) of SH-SY5Y cells co-expressing mCherry or mCherry-TDP-CTF with GFP, GFP-KPNB1 full-length (FL), H1–9 or H10–19. Both full-length and the N-terminal half of KPNB1 reduce TDP-CTF aggregation, whereas the C-terminal half of KPNB1 co-aggregates with TDP-CTF in the cytoplasm. Arrowheads indicate co-aggregation. Hoechst staining was used to outline nuclei. Scale bar: 5 μm. **b** Western blot analysis and quantification of insoluble mCherry-TDP-CTF protein levels in SH-SY5Y cells expressing GFP, GFP-KPNB1 full-length (FL), H1–9 or H10–19. Both full-length and the N-terminal half of KPNB1 significantly reduced insoluble TDP-CTF levels. β-tubulin was used as a loading control. Statistical analysis was performed using one-way ANOVA and Bonferroni’s post hoc test (**p < 0.01, ***p < 0.001, *n* = 4). **c** Quantification of TDP-CTF-induced cytotoxicity upon overexpression of KPNB1. mCherry or mCherry-TDP-CTF were co-expressed in SH-SY5Y cells with GFP, GFP-KPNB1 full-length (FL), H1–9 or H10–19. Forty-eight hours after transfection, cells were labelled with the Live-or-Dye™ 640/662 dye and dying cells were quantified for each group. Both full-length and the N-terminal half of KPNB1 significantly ameliorated TDP-CTF-mediated toxicity. Statistical analysis was performed with two-way ANOVA and Bonferroni’s post hoc test (four independent experiments; ***p < 0.001. mCherry: GFP (*n* = 400), GFP-KPNB1^FL^ (*n* = 405), GFP-KPNB1^H1–9^ (*n* = 426), GFP-KPNB1^H10–19^ (*n* = 406); mCherry-TDP-CTF: GFP (n = 405), GFP-KPNB1^FL^ (*n* = 401), GFP-KPNB1^H1–9^ (*n* = 405), GFP-KPNB1^H10–19^ (*n* = 400)). **d** IF of SH-SY5Y cells co-expressing mCherry or mCherry-TDP-CTF with GFP-KPNB1 H1–9 or C-terminal deletion constructs. TDP-CTF appeared nucleocytoplasmic with KPNB1 H1–9 and H1–8, while it formed small aggregates in presence of KPNB1 H1–7 or H1–6. Arrowheads indicate co-aggregation. Hoechst staining was used to outline nuclei. Scale bar: 5 μm. **e,** Western blot analysis and quantification of insoluble mCherry-TDP-CTF protein levels in SH-SY5Y cells expressing GFP, GFP-KPNB1 full-length (FL), H1–9 or C-terminal deletion constructs. KPNB1 H1–8 was the most active fragment in reducing insoluble TDP-CTF levels. β-actin was used as a loading control. Statistical analysis was performed using one-way ANOVA and Bonferroni’s post hoc test (*p < 0.05, **p < 0.01, ***p < 0.001, *n* = 4)

We focused on KPNB1, the best characterized member of the NIR family, to gain a better mechanistic understanding of how NIRs reduce TDP-CTF aggregation and determine the region that is required and sufficient for this activity. Since KPNB1 is comprised of 19 highly flexible arrays of short amphiphilic tandem α-helical HEAT repeats, we generated a series of constructs based on these repeats. First, we tested the N-terminal (HEAT repeats 1–9, H1–9) and the C-terminal half of KPNB1 (H10–19). Much like the full-length protein, KPNB1 H1–9 showed diffuse localization with enrichment in the nuclear envelope, whereas KPNB1 H10–19 was mainly present in the nucleoplasm (Fig. 2a). Expression of full-length KPNB1 and H1–9, but not H10–19, reduced TDP-CTF aggregate formation (Fig. 2a) and decreased detergent-insoluble levels of TDP-CTF (Fig. 2b) without affecting endogenous insoluble TDP-43 protein levels (Supplementary Fig. 2). Co-expression of full-length KPNB1 or H1–9 also significantly reduced TDP-CTF-induced cell death by ~ 58%, whereas KPNB1 H10–19 had no effect (Fig. 2c), demonstrating that the N-terminal part of KPNB1 is required and sufficient to reduce cytoplasmic TDP-43 aggregation and toxicity.

To further delineate the active region in the KPNB1 H1–9 fragment, we generated a series of constructs with C-terminal HEAT repeat deletions, ranging from H1–8 down to H1. Only KPNB1 H1–8 was strongly enriched on the nuclear envelope, and was identified as the smallest fragment with full activity towards reducing TDP-CTF aggregation, rendering TDP-CTF diffuse and significantly decreasing insoluble TDP-CTF protein levels by ~ 77% (Fig. 2d,e). KPNB1 H1–7 and KPNB1 H1–6 were less active, only slightly reducing insoluble TDP-CTF protein levels and aggregate size, while even smaller KPNB1 fragments colocalized with TDP-CTF aggregates without exerting any significant chaperone activity to reduce aggregation and restore solubility (Fig. 2d,e).

### Reduction of TDP-CTF aggregation depends on the FG-Nup interaction domain of KPNB1

**Fig. 3:**
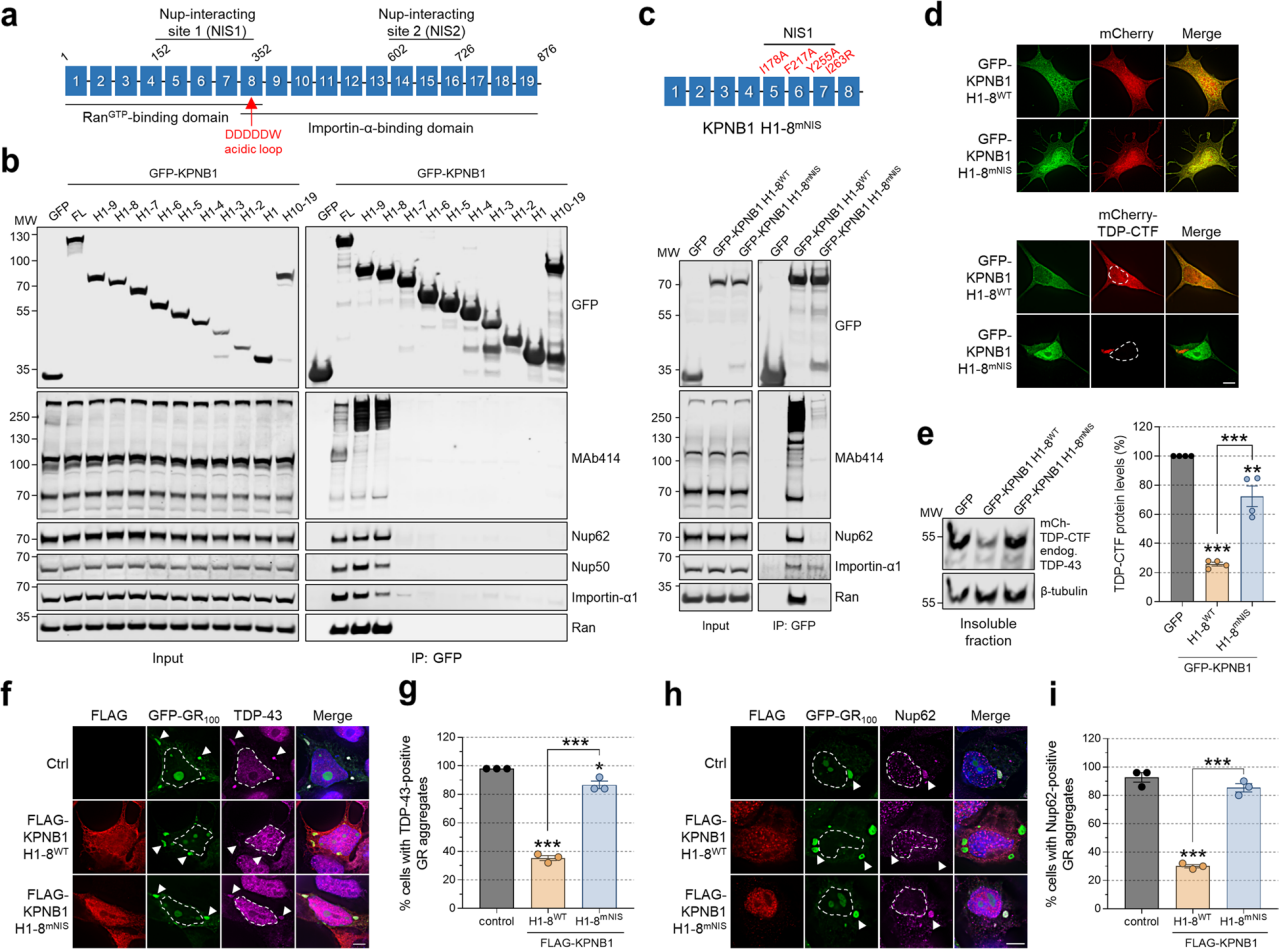
KPNB1-mediated reduction of TDP-CTF and TDP-43 aggregates depends on its FG-Nup interaction domain. **a** Schematic domain structure of KPNB1. KPNB1 is comprised of 19 HEAT repeats (H1–19). Ran^GTP^ and importin-α interact with H1–8 and H8–19, respectively. FG-Nups bind to two regions of KPNB1, H5–7 and H14–16. **b** Lysates from HEK293T cells expressing GFP, GFP-KPNB1 full-length (FL), N-terminal fragments of KPNB1 (H1–9…H1) or the C-terminal fragment of KPNB1 (H10–19) were subjected to immunoprecipitation with GFP-Trap magnetic beads. Whole cell lysates (input) and immunoprecipitates (IP) were subjected to western blot analysis using indicated antibodies. KPNB1 H1–8, the smallest fully active KPNB1 fragment in reducing TDP-43 aggregation, strongly interacts with FG-Nups and Ran, and weakly with importin-α1 in comparison with full-length KPNB1. **c** (Top) Schematic domain structure of KPNB1 H1–8^mNIS^ harboring four missense mutations (I178A, F217A, Y255A, I263R) in the nucleoporin-interacting site (NIS). (Bottom) Lysates from HEK293T cells expressing GFP, GFP-KPNB1 H1–8^WT^ or H1–8^mNIS^ were subjected to immunoprecipitation with GFP-Trap magnetic beads. Whole cell lysates (input) and immunoprecipitates (IP) were subjected to western blot analysis using indicated antibodies. KPNB1 H1–8^mNIS^ shows strongly reduced interaction with FG-Nups. **d** Immunofluorescence (IF) of SH-SY5Y cells co-expressing mCherry or mCherry-TDP-CTF with GFP-KPNB1 H1–8^WT^ or H1–8^mNIS^. KPNB1 H1–8^mNIS^ does not reduce cytoplasmic TDP-CTF aggregates. Hoechst staining was used to outline nuclei. Scale bar: 5 μm. **e** Western blot analysis and quantification of insoluble mCherry-TDP-CTF in SH-SY5Y cells expressing GFP, GFP-KPNB1 H1–8^WT^ or H1–8^mNIS^. KPNB1 H1–8^WT^ strongly decreased insoluble TDP-CTF levels, whereas KPNB1 H1–8^mNIS^ did not. β-tubulin was used as a loading control. Statistical analysis was performed using one-way ANOVA and Bonferroni’s post hoc test (**p < 0.01, ***p < 0.001, *n* = 4). **f** IF of HEK293T cells co-expressing GFP-(GR)_100_ with an empty plasmid (ctrl), FLAG-tagged KPNB1 H1–8^WT^ or H1–8^mNIS^ and stained for endogenous TDP-43. Unlike KPNB1 H1–8^mNIS^, KPNB1 H1–8^WT^ prevents the sequestration of endogenous TDP-43 in cytoplasmic GR aggregates. Arrowheads point to cytoplasmic GR aggregates. Hoechst staining was used to outline nuclei. Scale bar: 5 μm. **g** Quantification of the percentage of cells with cytoplasmic GR aggregates positive for endogenous TDP-43. KPNB1 H1–8^WT^ significantly reduced the number of TDP-43-positive GR aggregates, while KPNB1 H1–8^mNIS^ had no effect. Statistical analysis was performed using one-way ANOVA and Bonferroni’s post hoc test (three independent experiments; *p < 0.05, ***p < 0.001, *n* = 50–52 cells per group). **h** IF of HEK293T cells co-expressing GFP-(GR)_100_ with an empty plasmid (ctrl), FLAG-tagged KPNB1 H1–8^WT^ or H1–8^mNIS^ and stained for endogenous Nup62. Unlike KPNB1 H1–8^mNIS^, KPNB1 H1–8^WT^ prevents the sequestration of endogenous Nup62 in cytoplasmic GR aggregates. Arrowheads point to cytoplasmic GR aggregates. Hoechst staining was used to outline nuclei. Scale bar: 5 μm. **i** Quantification of the percentage of cells with cytoplasmic GR aggregates positive for endogenous Nup62. KPNB1 H1–8^WT^ significantly reduced the number of Nup62-positive GR aggregates, while KPNB1 H1–8^mNIS^ had no effect. Statistical analysis was performed using one-way ANOVA and Bonferroni’s post hoc test (three independent experiments; ***p < 0.001, *n* = 50–51 cells per group)

Numerous studies have mapped binding sites for KPNB1 interactors that regulate its nuclear import activity, including Ran^GTP^, importin-α, and FG-Nups which can bind KPNB1 at two distinct Nup-interacting sites (NIS1 and 2) [35, 36] (Fig. 3a). We used co-immunoprecipitation (co-IP) experiments with KPNB1 constructs to map high-affinity interactions with FG-Nups including Nup62 and Nup50, but also importin-α1, and Ran. KPNB1 H1–9 and H1–8 interacted with high-molecular weight FG-Nups with even greater affinity than full-length KPNB1 (Fig. 3b). While structural studies have shown that importin-α contacts KPNB1 H7–19 [37], our results indicate that H1–8, perhaps via the acidic loop in H8, may be required and sufficient for this interaction.

KPNB1 constructs with the greatest effect on TDP-CTF aggregation are also the constructs that bind to FG-Nups; this raised the question whether the proposed chaperone activity of KPNB1 depends on its interaction with FG-Nups. We therefore introduced missense mutations (I178A, F217A, Y255A, I263R) into the NIS1 of KPNB1 H1–8 (H1–8^mNIS^) to reduce FG-Nup binding [38, 39] (Fig. 3c). While KPNB1 H1–8^WT^ expression caused diffuse nucleocytoplasmic distribution of TDP-CTF, reduced the formation of TDP-CTF cytoplasmic inclusions, and decreased insoluble TDP-CTF, KPNB1 H1–8^mNIS^ had lost most of its activity (Fig. 3d,e). It should be noted that KPNB1 H1–8^mNIS^ is slightly more nuclear than the wild-type protein, which could contribute to its inability to antagonize cytoplasmic aggregation of TDP-CTF (Fig. 3d).

To determine whether KPNB1 can also reduce the aggregation of cytoplasmically mislocalized endogenous TDP-43, we used a C9ALS/FTD disease model based on expression of a *C9orf72* repeat expansion-derived poly(GR) dipeptide repeat protein, which has been shown to induce endogenous TDP-43 mislocalization in mice [40, 41]. pTDP-43-positive GR aggregates have also been detected in dendritic inclusions in the motor cortex of C9-ALS patients [42]. We found that GFP-poly(GR)_100_ forms both nuclear and cytoplasmic aggregates when expressed in HEK293T cells, but only cytoplasmic aggregates were positive for endogenous TDP-43 and Nup62 (Fig. 3f,h and Supplementary Fig. 3). We observed that some cells with nuclear GR aggregates exhibited an irregular nuclear envelope, nucleocytoplasmic distribution of TDP-43, and abnormal aggregation of Nup62 in the cytoplasm (Supplementary Fig. 3a). While KPNB1 H1–8^WT^ did not affect GR inclusion formation, it abolished the accumulation of TDP-43 and Nup62 in these aggregates in ~ 60% of cells (Fig. 3f-i). This effect was dependent on the FG-Nup interaction domain as most GR aggregates remained positive for TDP-43 and Nup62 in the presence of KPNB1 H1–8^mNIS^ (Fig. 3f-i). Taken together, these results suggest a role for FG-Nups in facilitating KPNB1-mediated reduction of TDP-43 aggregation.

### KPNB1 associates with TDP-CTF via both the RRM2 and PrLD domain

To identify the TDP-CTF regions required for recruiting KPNB1, we conducted a series of interaction domain mapping experiments. Co-IP experiments in HEK293T cell lysates showed that GFP-TDP-CTF strongly interacts with untagged KPNB1 both via the remaining RRM2 domain fragment present in TDP-CTF (non-PrLD, aa208–274), and to a lesser degree its PrLD (aa275–414) (Supplementary Fig. 4).

To delineate the interaction domain within the non-PrLD of TDP-CTF, we tested a series of TDP-CTF deletion constructs (Supplementary Fig. 5a) in co-IP experiments with KPNB1 and mapped the main interaction domain to TDP-CTF amino-acids 230–240 (Supplementary Fig. 5b,c). This was confirmed by a TDP-CTF deletion construct lacking these ten amino-acids (TDP-CTF^Δ230–240^) that interacts significantly weaker with KPNB1 in comparison to TDP-CTF^WT^ (Supplementary Fig. 5d). Unexpectedly, KPNB1 was still able to reduce TDP-CTF^Δ230–240^ aggregates and insoluble protein levels, similar to TDP-CTF^WT^ (Supplementary Fig. 5e,f). We therefore concluded that although region 230–240 in TDP-CTF can mediate interaction with KPNB1, it is not required for its TDP-CTF aggregation-reducing activity.

**Fig. 4:**
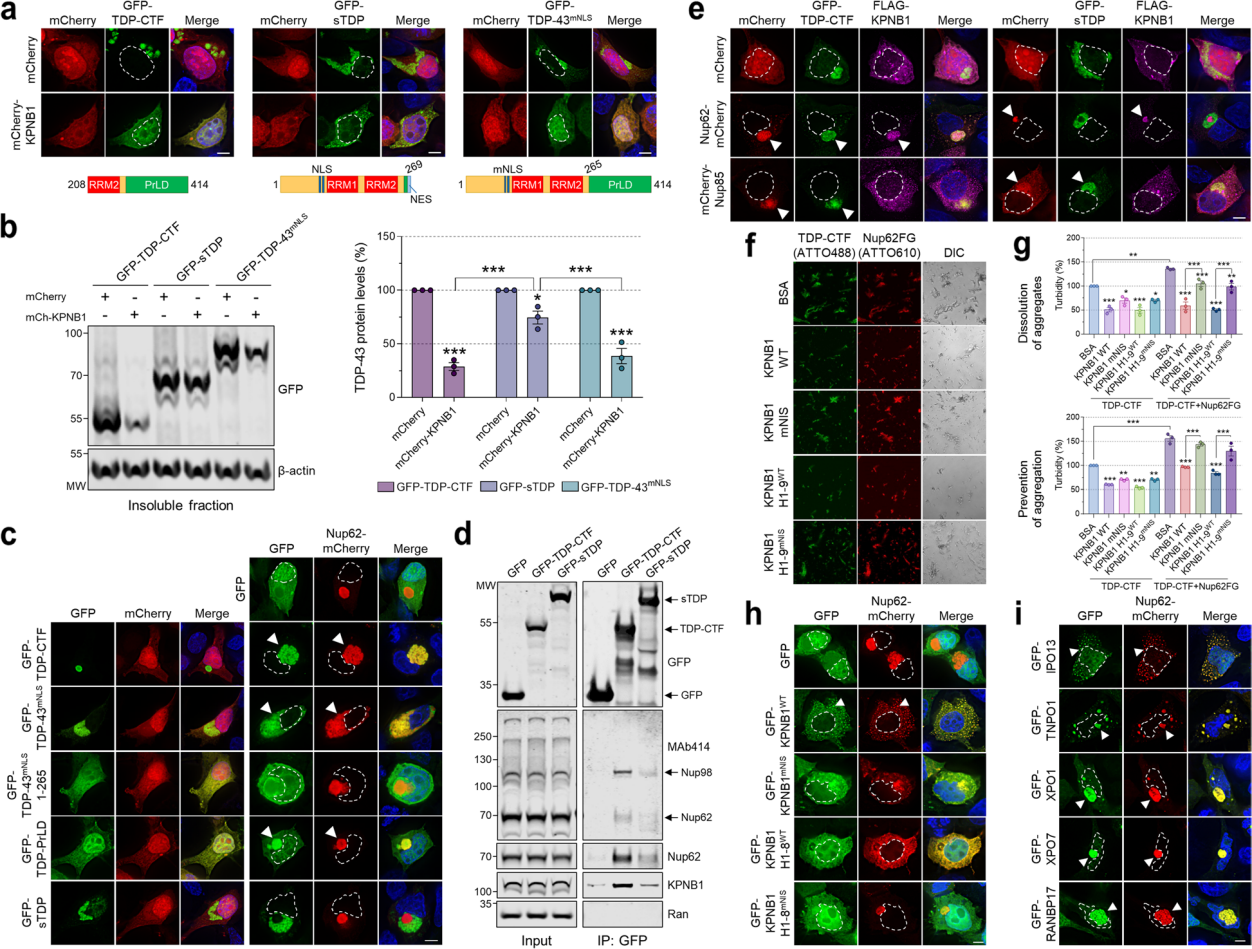
Nup62 co-aggregates with TDP-43 PrLD and recruits KPNB1 to facilitate reduction of aggregation. **a** Immunofluorescence (IF) of HEK293T cells co-expressing GFP-tagged TDP-CTF, sTDP or TDP-43^mNLS^ with mCherry or mCherry-KPNB1. KPNB1 abolished TDP-CTF and TDP-43^mNLS^ aggregates, whereas sTDP aggregates were only reduced in size. Hoechst staining was used to outline nuclei. Scale bar: 5 μm. **b** Western blot analysis and quantification of insoluble GFP-tagged TDP-CTF, sTDP and TDP-43^mNLS^ in HEK293T cells expressing mCherry or mCherry-KPNB1. KPNB1 strongly reduced insoluble TDP-CTF and TDP-43^mNLS^ protein levels, and sTDP levels to a lesser extent. β-actin was used as a loading control. Statistical analysis was performed using two-way ANOVA and Bonferroni’s post hoc test (*p < 0.05, ***p < 0.001, *n* = 3). **c** IF of HEK293T cells co-expressing mCherry or Nup62-mCherry with GFP or GFP-tagged TDP-CTF, TDP-43^mNLS^, TDP-43^mNLS^ 1–265, TDP-PrLD or sTDP. TDP-CTF and TDP-43^mNLS^ strongly colocalize with Nup62 via their PrLD, whereas sTDP lacking this domain does not. Arrowheads indicate co-aggregation. Hoechst staining was used to outline nuclei. Scale bar: 5 μm. **d** Lysates from HEK293T cells expressing GFP, GFP-TDP-CTF or GFP-sTDP were subjected to immunoprecipitation with GFP-Trap magnetic beads. Whole cell lysates (input) and immunoprecipitates (IP) were subjected to western blot analysis using indicated antibodies. Unlike sTDP, TDP-CTF strongly interacts with endogenous Nup62 and KPNB1. **e** IF of HEK293T cells co-expressing mCherry/Nup62-mCherry/mCherry-Nup85, GFP-TDP-CTF/GFP-sTDP and FLAG-KPNB1. Nup62 recruits KPNB1 to TDP-CTF but not sTDP aggregates. Nup85 which lacks FG repeats does not recruit KPNB1 to either TDP-CTF or sTDP, although it colocalizes with both aggregates. Arrowheads indicate co-aggregation. Hoechst staining was used to outline nuclei. Scale bar: 5 μm. **f** Dissolution assays for fluorophore-labeled recombinant His_6_-tagged TDP-CTF (ATTO488) and Nup62FG (ATTO610) co-aggregates in the presence of BSA or GST-tagged KPNB1 WT, KPNB1 mNIS, H1–9^WT^ or H1–9^mNIS^. KPNB1 and H1–9 WT strongly dissolved TDP-CTF–Nup62 aggregates, whereas NIS mutations abolished this activity. **g** Quantification of dissolution of pre-formed aggregates (top) and prevention of aggregation (bottom) assays of TDP-CTF in the presence or absence of Nup62FG and different KPNB1 constructs. Optical density at 395 nm was measured to assess turbidity. Statistical analysis was performed using one-way ANOVA and Bonferroni’s post hoc test (*p < 0.05, **p < 0.005, ***p < 0.001, *n* = 3). **h** IF of HEK293T cells co-expressing Nup62-mCherry with GFP or GFP-tagged KPNB1^WT^, KPNB1^mNIS^, KPNB1 H1–8^WT^ or H1–8^mNIS^. KPNB1^WT^ associated with Nup62 aggregates and strongly reduced their size, whereas KPNB1 H1–8^WT^ rendered Nup62 completely soluble. Arrowheads indicate co-aggregation. Hoechst staining was used to outline nuclei. Scale bar: 5 μm. **i** IF of HEK293T cells co-expressing Nup62-mCherry with GFP or GFP-tagged IPO13, TNPO1, XPO1, XPO7 or RANBP17. IPO13 associated with Nup62 aggregates and strongly reduced their size. TNPO1 and exportins co-aggregated with Nup62 but only TNPO1 slightly reduced individual aggregate size. Arrowheads indicate co-aggregation. Hoechst staining was used to outline nuclei. Scale bar: 5 μm

These findings led us to determine whether the PrLD is required for reducing TDP-43 aggregation, by co-expressing KPNB1 with TDP-CTF, TDP-43^mNLS^, or sTDP (short-TDP), an alternative splicing isoform of TDP-43 where the PrLD is replaced by a nuclear export signal (NES) [43]. KPNB1 expression not only reduced TDP-CTF and TDP-43^mNLS^ aggregates, but also increased the nuclear localization of TDP-43^mNLS^ (Fig. 4a). In contrast, sTDP cytoplasmic aggregates were reduced in size but still present upon KPNB1 expression. Western blot analysis of insoluble TDP-43 protein showed that KPNB1 reduced the levels of all three TDP-43 constructs, but sTDP to a much lesser extent (Fig. 4b), indicating an NLS-independent mode of interaction and an important role for the PrLD in enabling KPNB1 to reduce TDP-43 protein aggregation.

### Nup62 associates with the TDP-43 PrLD and recruits KPNB1 into aggregates

The PrLD of TDP-43 is a highly intrinsically disordered region (IDR) that mediates liquid-liquid phase separation (LLPS) to form transient biomolecular condensates under normal physiological conditions, and adopts a unique double spiral-shaped fold to form pathological amyloid-like filament structures under disease conditions [44]. The TDP-43 PrLD also mediates its multivalent interactions with other proteins including FG-Nups such as Nup98, Nup153 and Nup214 [16]. The TDP-43 PrLD fragment (TDP-PrLD, aa275–414) is mostly soluble in cells but sometimes forms small cytoplasmic granules that are positive for Nup62 and KPNB1 (Supplementary Fig. 6a,b). Since we found that the aggregation-reducing activity of KPNB1 depends on its FG-Nup and Ran^GTP^ interaction domain (Fig. 3), we hypothesized that either FG-Nups or Ran recruit KPNB1 to TDP-43 aggregates. Pull-down experiments with HEK293T cell lysates confirmed that TDP-PrLD interacts with FG-Nups Nup62 and Nup98, and KPNB1, but not with Ran (Supplementary Fig. 6c), suggesting that Ran is not required for the association between TDP-CTF and KPNB1. To investigate the role of FG-Nups in recruiting KPNB1 to TDP-43 aggregates, we tested a battery of GFP-tagged TDP-43 constructs for colocalization with mCherry-tagged Nup62, which by itself forms big cytoplasmic aggregates in cell culture and has been previously shown to colocalize with TDP-43 aggregates [16, 45]. HEK293T cells were selected for these experiments due to their robust protein aggregation and resistance to TDP-43 toxicity compared to neuronal lines. We found that TDP-CTF, TDP-43^mNLS^ and TDP-PrLD strongly colocalize with Nup62-mCherry (Fig. 4c and Supplementary Fig. 7). While the N-terminal half of TDP-43^mNLS^ (TDP-43^mNLS^ 1–265) and TDP-PrLD are soluble when co-expressed with mCherry, only TDP-PrLD co-aggregates with Nup62-mCherry. Strikingly, sTDP aggregates that lack the PrLD do not colocalize with Nup62 but accumulate around Nup62 inclusions (Fig. 4c and Supplementary Fig. 7). Similar to Nup62, Nup98 also strongly colocalized with TDP-CTF, TDP-43^mNLS^ and TDP-PrLD, but not sTDP (Supplementary Fig. 10). sTDP also showed reduced interaction with endogenous Nup62, Nup98, and KPNB1, as compared to TDP-CTF (Fig. 4d). These findings may explain why KPNB1 cannot reduce sTDP aggregation as efficiently as TDP-CTF (Fig. 4a,b). In comparison with TDP-43^mNLS^, sTDP lacks the PrLD but also has an intact NLS and an additional C-terminal NES that could interfere with the TDP-43–Nup62 interaction. We therefore co-expressed a sTDP construct that lacks both sequences (sTDP^mNLS^ ΔNES) with Nup62-mCherry and found it to still form aggregates surrounding Nup62 foci (Supplementary Fig. 8), confirming that it is the TDP-43 PrLD that is required for Nup62 association.

We next tested whether expression of Nup62 can increase the association between TDP-CTF aggregates and KPNB1 in HEK293T cells. KPNB1 strongly reduces the prevalence of TDP-CTF aggregates; the rare remaining TDP-CTF aggregates weakly overlap with KPNB1, and co-expression of Nup62 dramatically increased the recruitment of KPNB1 into TDP-CTF aggregates (Fig. 4e). As expected, Nup62 and KPNB1 did not colocalize with sTDP aggregates. While the non-FG nucleoporin Nup85 showed strong colocalization with TDP-CTF, it did not recruit KPNB1 into aggregates (Fig. 4e). These findings raised the question whether this FG-Nup-dependent recruitment of NIRs into TDP-43 aggregates may contribute to their aggregation-reducing activity.

### The association of TDP-43 PrLD with Nup62 is required for KPNB1-mediated disaggregation

To test the hypothesis that Nup62 directly mediates disaggregation of TDP-43 PrLD by KPNB1, we first performed in vitro protein dissolution assays where we measured the turbidity of solutions containing a mix of recombinant TDP-CTF protein with or without KPNB1 and Nup62FG (aa1–268). For disaggregation assays, TDP-CTF and Nup62FG were incubated together for 30 min to allow the formation of aggregates, followed by KPNB1 addition for 24 h, whereas for prevention experiments all three proteins were mixed and incubated for 30 min. TDP-CTF and Nup62FG strongly co-aggregated in vitro, and KPNB1 WT and H1–9^WT^ strongly dissolved pre-formed aggregates and prevented the formation of these co-aggregates (Fig. 4f,g). Interestingly, NIS mutations in recombinant KPNB1 almost completely abolished its activity towards TDP-CTF and Nup62FG co-aggregates (Fig. 4f,g). In the absence of Nup62FG, all KPNB1 constructs appeared to reduce the solution turbidity at a lower level and independent of the NIS domain (Fig. 4g). This shows that under in vitro conditions high concentrations of recombinant KPNB1 are still able to reduce TDP-43 aggregation in absence of FG-Nups, similarly to studies on recombinant TNPO1 and FUS protein lacking the PY-NLS [18, 19]. Importantly, these data confirm that the chaperone and disaggregation activity of KPNB1 towards TDP-CTF and Nup62FG co-aggregates is KPNB1 NIS-dependent and TDP-43 NLS-independent.

To test whether specific mutations in the PrLD of TDP-43 that hinder its association with Nup62 also inhibit the KPNB1-dependent reduced aggregation, we generated an extensive series of PrLD mutant variants (Supplementary Fig. 9). FUSΔ14 was used as a negative control, as FUS aggregates do not colocalize with Nup62 (Supplementary Fig. 9d). Deleting any of the three segments that make up the PrLD (Δ274–313, Δ314–353 or Δ354–393) did not prevent co-aggregation with Nup62, suggesting a multivalent interaction across the PrLD instead of one specific interaction region (Supplementary Fig. 9a). Since the TDP-43 PrLD harbors six FG motifs, including four GXFG repeats (Supplementary Fig. 9e), we sought to determine whether TDP-CTF and Nup62 can associate via their respective FG repeats. However, TDP-CTF mutants where the phenylalanine residues in the PrLD were replaced by alanine, glycine or tyrosine residues (F-A, F-G or F-Y) still strongly co-aggregated with Nup62 (Supplementary Fig. 9a). Following up on a systematic mutagenesis study of TDP-43 phase separation dynamics [46], we also interrogated the role of charged (KRED), aliphatic (VLIM), and aromatic (FYW) amino-acid residues for PrLD association with Nup62. Only one of these mutations had a strong effect on TDP-CTF colocalization with Nup62: substituting nine aliphatic residues with phenylalanine (VLIM-F, mutations shown in Supplementary Fig. 9e) not only rendered TDP-CTF^VLIM-F^ more aggregation-prone, but also abrogated its colocalization with Nup62 (Supplementary Fig. 9a,b) and Nup98 (Supplementary Fig. 10). Similarly, TDP-43^mNLS^ aggregates with VLIM-F mutations in the PrLD were proximal to Nup62 granules but did not overlap (Supplementary Fig. 9c). This allowed us to investigate whether reduced Nup62 association would impact KPNB1-dependent reduction of aggregation. Indeed, when compared with TDP-CTF^WT^, TDP-CTF^VLIM-F^ interaction with KPNB1 was reduced in co-IP experiments (Supplementary Fig. 9f) and its insoluble protein levels were only slightly affected by KPNB1 (Supplementary Fig. 9g,h). While it is possible that the recruitment of FG-Nups and KPNB1 into TDP-CTF^VLIM-F^ inclusions is prevented by their dense nature, other TDP-CTF mutations promoting its aggregation did not affect association with Nup62. Taken together, our results strongly suggest that FG-Nup interaction plays a crucial role in recruiting KPNB1 to reduce TDP-43 protein aggregation.

### KPNB1 reduces TDP-CTF and Nup62 aggregates in a similar manner

One compelling model for the canonical activity of NIRs in nuclear import is based on their structural flexibility that allows them to form multivalent interactions with FG motifs. Disrupting hydrophobic interactions in the FG-Nup hydrogel facilitates the transport of cargo across the crowded nuclear pore barrier [34]. This suggested to us that KPNB1 may dissolve or prevent Nup62–TDP-43 co-aggregate formation via a similar mechanism. Indeed, co-expression of Nup62 with KPNB1^WT^ leads to the formation of smaller Nup62 aggregates that strongly colocalize with KPNB1, whereas KPNB1^mNIS^ weakly associated with Nup62 aggregates and had almost no effect on their size (Fig. 4h). Similarly, KPNB1 H1–8^WT^ abolished Nup62 aggregates, while KPNB1 H1–8^mNIS^ did not (Fig. 4h and Supplementary Fig. 11).

We had observed that only a subset of β-importins can reduce TDP-CTF aggregates, although importins and exportins interact with Nup62 and other FG-Nups during nuclear transport. IPO13 reduces TDP-CTF aggregation even more strongly that KPNB1, whereas TNPO1 partially disassembles TDP-CTF into smaller aggregates, and exportins XPO1, XPO7, and RANBP17 co-aggregate with TDP-CTF (Fig. 1a). We co-expressed these transport receptors with Nup62-mCherry and found a very similar effect on Nup62 (Fig. 4i). All three exportins strongly associated with Nup62 aggregates, but without affecting their morphology, whereas IPO13 caused strong dissociation and TNPO1 partially disassembled Nup62 into smaller aggregates (Fig. 4i). These results indicate that specific transport receptors have a similar effect on both TDP-43 and Nup62 aggregates, suggesting a common mechanism.

### The fly ortholog of KPNB1 rescues TDP-43 pathology and toxicity in fly models of TDP-43 proteinopathy

**Fig. 5:**
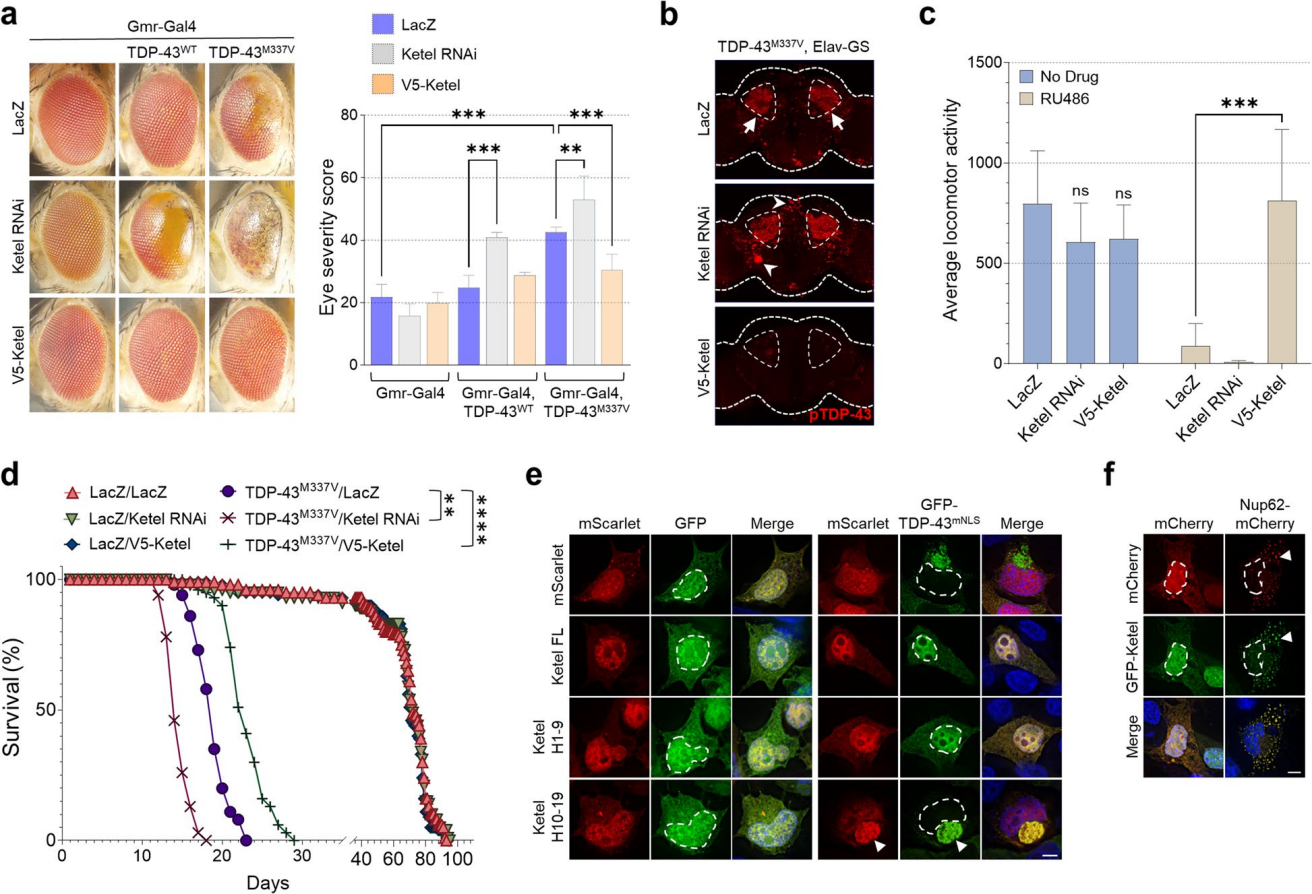
The *Drosophila* KPNB1 ortholog Ketel reduces human TDP-43 pathology and toxicity in fly models of TDP-43 proteinopathy. **a** Eye phenotypes from flies expressing the indicated transgenes via the eye-specific Gmr-Gal4 driver. Ketel knock-down (RNAi) enhances TDP-43-induced neurodegeneration whereas its overexpression (V5-Ketel) rescues it. Statistical analysis was performed using one-way ANOVA and Bonferroni’s post hoc test (**p < 0.01, ***p < 0.001, *n* ≥ 4). **b** Immunostaining of phosphorylated TDP-43^M337V^ (pTDP-43) in adult fly brains treated with RU486 (40 μg/ml). This conditional *Drosophila* model of TDP-43 proteinopathy is based on expression of mutant human TDP-43^M337V^ under the control of the Elav-GS driver in adult flies upon administration of RU486. Ketel RNAi enhances and extends the accumulation of pTDP-43-positive aggregates in the fly brain (arrowheads), compared to LacZ control (arrows), whereas overexpression of V5-Ketel dramatically reduces the pTDP-43 staining. **c** Locomotion assays on flies expressing mutant TDP-43^M337V^ under the control of the Elav-GS driver in the presence or absence of RU486. Ketel downregulation enhances mutant TDP-43-induced locomotor dysfunction in adult flies, whereas Ketel overexpression rescues it. Locomotion was assessed with the DAM2 activity monitor from TriKinetics, Inc. Statistical analysis was performed using two-way ANOVA and Bonferroni’s post hoc test (***p < 0.001, *n* = 13). **d** Survival curves of flies expressing the indicated transgenes under the control of the Elav-GS driver in the presence of the drug RU486. Flies co-expressing TDP-43^M337V^ with the innocuous transgene LacZ have a dramatic lifespan reduction in the presence of RU486 (blue line) compared to flies lacking mutant TDP-43 (gray line). Co-expression of Ketel (green line) improves this phenotype, whereas a Ketel RNAi transgene (red line) exacerbates it. Survival analysis was performed using the OASIS online tool, and *p*-values were calculated using Fisher’s exact test (**p < 0.01 and ****p < 0.0001, *n* = 80). **e** Immunofluorescence (IF) of HEK293T cells co-expressing GFP or GFP-TDP-43^mNLS^ with mScarlet or mScarlet-tagged Ketel full-length (FL), H1–9 or H10–19. Both full-length and the N-terminal half of Ketel reduce human TDP-43^mNLS^ aggregation and cytoplasmic mislocalization, whereas the C-terminal half of Ketel co-aggregates with TDP-43^mNLS^. Arrowheads indicate co-aggregation. Hoechst staining was used to outline nuclei. Scale bar: 5 μm. **f** IF of HEK293T cells co-expressing GFP-Ketel with mCherry or Nup62-mCherry. While Ketel is mostly nuclear with mCherry, it strongly colocalizes with Nup62-mCherry aggregates in the cytoplasm. Arrowheads indicate co-aggregation. Hoechst staining was used to outline nuclei. Scale bar: 5 μm

In a large-scale RNAi screen based on an easy-to-score eye phenotype induced by expression of human TDP-43^M337V^ in transgenic flies [25], we identified *Fs(2)Ket* (*Ketel*), the fly ortholog of human KPNB1, as one of the most robust enhancers of TDP-43 toxicity, thus confirming our findings from mammalian cells (full details of the screen will be published elsewhere). Expression of human TDP-43^WT^ alone causes a very modest degenerative phenotype, whereas mutant TDP-43^M337V^ shows partial cytoplasmic mislocalization and pathological hyperphosphorylation, leading to severe neurodegeneration [25]. While RNAi inhibition of Ketel did not perturb the eye morphology in control flies, it exacerbated retinal degeneration associated with TDP-43^WT^ and TDP-43^M337V^ (Fig. 5a), a result that was confirmed with multiple Ketel RNAi lines (Supplementary Fig. 12). To validate this genetic interaction, we generated flies carrying a UAS-Ketel transgene and demonstrated that overexpression of Ketel rescued the strong TDP-43^M337V^ eye phenotype almost completely (Fig. 5a). Next, we used a conditional system where pan-neuronal transgene expression is induced in adult flies and found that expression of TDP-43^M337V^ resulted in accumulation of phosphorylated TDP-43 in the central brain (Fig. 5b, arrows). Strikingly, downregulation of Ketel promoted phosphorylation of mutant TDP-43 but Ketel overexpression dramatically reduced it (Fig. 5b), without affecting TDP-43 total protein levels (Supplementary Fig. 13). Moreover, pan-neuronal expression of TDP-43^M337V^ induced a very aggressive locomotor dysfunction, while co-expression with Ketel completely rescued this phenotype (Fig. 5c). These results are consistent with lifespan reduction or extension in TDP-43^M337V^ flies when Ketel is down- or upregulated, respectively (Fig. 5d and Supplementary Fig. 14). Of note, an EP-insertion line (EP-Ketel) predicted to overexpress the *Fs(2)Ket* gene also rescued pathological and behavioral phenotypes induced by TDP-43^M337V^ (Supplementary Fig. 15). We verified that Ketel is also protective in pathological human TDP-43^ΔNLS^ expressing flies (Supplementary Fig. 16), which display abnormal accumulation and processing of TDP-43 in the cytoplasm [25]. In support of our finding that other NIRs can also reduce TDP-CTF aggregation (Fig. 1), we found that *cdm*, the fly homologue of IPO13, also alleviates the severe eye degeneration in TDP-43^ΔNLS^ flies (Supplementary Fig. 17).

To confirm whether Ketel expression targets TDP-43 pathology similarly to its human ortholog KPNB1 (~ 60% identity) [47] in mammalian cells, we co-expressed Ketel constructs with TDP-43^mNLS^ in HEK293T cells and found reduction of TDP-43 cytoplasmic aggregation and restored nuclear localization that was dependent on the N-terminal fragment of Ketel (Fig. 5e). Similar to human KPNB1, Ketel was recruited into Nup62-positive aggregates in these cells (Fig. 5f). In summary, *Drosophila* data from the genetic screen and follow-up studies very closely match our results in mammalian systems, strongly supporting a role for KPNB1/Ketel and other NIRs as protective modifiers of TDP-43 proteinopathy that specifically target pathological TDP-43 across diverse in vitro and in vivo models.

### KPNB1 expression restores the nuclear localization of mislocalized TDP-43 independently of its NLS in primary neurons and mouse brain tissue

**Fig. 6:**
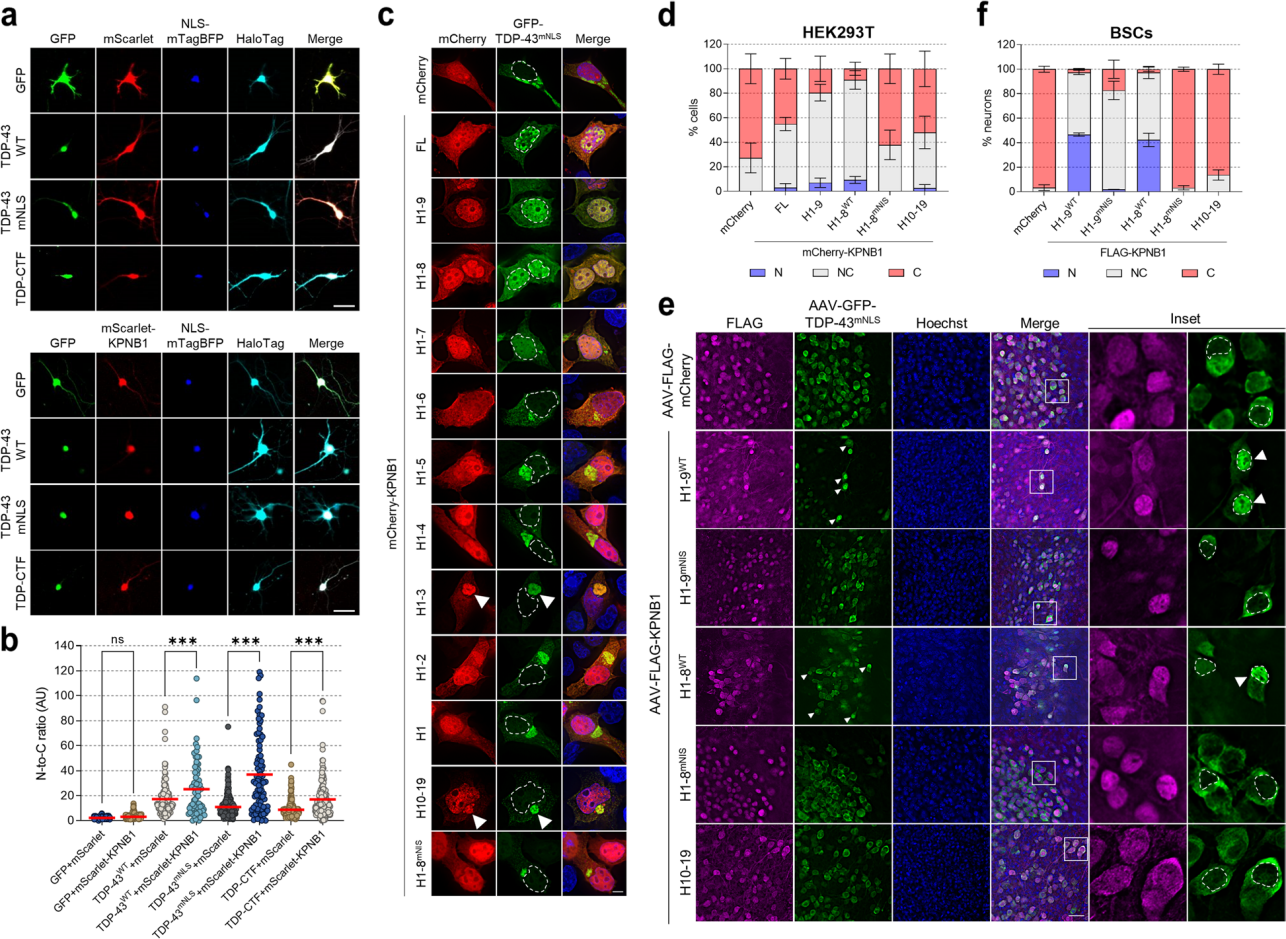
KPNB1 promotes nuclear localization of TDP-43^mNLS^ in primary neurons and mouse brain tissue. **a** Immunofluorescence (IF) of primary cortical neurons co-expressing NLS-mTagBFP (nuclear marker), HaloTag (cell marker), GFP or GFP-tagged TDP-43^WT^/TDP-43^mNLS^/TDP-CTF and mScarlet/mScarlet-KPNB1. KPNB1 increased the nuclear localization of TDP-43^mNLS^ and TDP-CTF. Scale bar: 25 μm. **b** Quantitative analysis of the N-to-C ratio of GFP or GFP-tagged TDP-43^WT^, TDP-43^mNLS^ or TDP-CTF in primary neurons expressing mScarlet or mScarlet-KPNB1 72 h post-transfection. Statistical analysis was performed using one-way ANOVA and Bonferroni’s post hoc test (**p < 0.01, ***p < 0.001, *n* = 52–264 neurons per group). **c** IF of HEK293T cells co-expressing GFP-TDP-43^mNLS^ with mCherry or mCherry-tagged KPNB1 full-length (FL) or its fragments. KPNB1 FL, H1–9 and H1–8 constructs increase the nuclear localization of TDP-43^mNLS^. Arrowheads point to co-aggregation. Hoechst staining was used to outline nuclei. Scale bar: 5 μm. **d** Quantification of the percentage of HEK293T cells exhibiting nuclear (N), nucleocytoplasmic (NC) or cytoplasmic (C) GFP-TDP-43^mNLS^ distribution upon expression of different mCherry-KPNB1 constructs. Statistical analysis was performed using two-way ANOVA and Bonferroni’s post hoc test (three independent experiments; *n* = 66–81 cells per group; statistics are summarized in Supplementary Table 2). **e** IF of mouse brain slice cultures (DIV12) co-expressing AAV-GFP-TDP-43^mNLS^ with AAV-FLAG-mCherry or AAV-FLAG-KPNB1 H1–9^WT^, H1–9^mNIS^, H1–8^WT^, H1–8^mNIS^ or H10–19. KPNB1 H1–9^WT^ and H1–8^WT^ increase the nuclear localization of TDP-43^mNLS^, whereas NIS mutations abolished this effect. Hoechst staining was used to outline nuclei. Scale bar: 50 μm. **f** Quantification of the percentage of neurons exhibiting nuclear (N), nucleocytoplasmic (NC) or cytoplasmic (C) GFP-TDP-43^mNLS^ distribution upon expression of different AAV-KPNB1 constructs. Statistical analysis was performed using two-way ANOVA and Bonferroni’s post hoc test (three independent experiments; *n* = 150–154 neurons per group; statistics are summarized in Supplementary Table 2)

Following up on the unexpected observation that KPNB1 expression increases the nuclear localization of TDP-43^mNLS^ (Fig. 4a), we measured the nucleocytoplasmic (N-to-C) ratio of TDP-43 constructs over time upon KPNB1 expression in primary neuron cultures. As expected, KPNB1 further increased the N-to-C ratio of TDP-43^WT^ as soon as 24 h after transfection, despite KPNB1 being mostly nuclear (Fig. 6a,b and Supplementary Fig. 18). KPNB1 expression significantly increased the nuclear localization of cytoplasmic TDP-43^mNLS^ and TDP-CTF 48 and 72 h post-transfection, demonstrating that KPNB1 can increase the nuclear import of TDP-43 independently of its NLS (Fig. 6a,b and Supplementary Fig. 18).

To assess whether both aggregation-reducing and nuclear translocation activities reside in the same domains of KPNB1, we tested the effect of truncated KPNB1 constructs on the localization of TDP-43^mNLS^ in HEK293T cells. Full-length KPNB1, as well as KPNB1 H1–9 and H1–8 strongly increased the levels of nuclear TDP-43^mNLS^, whereas KPNB1 H1–7 had a weak nuclear import activity (Fig. 6c,d). In addition, KPNB1 H1–8^mNIS^ did not change the localization of TDP-43^mNLS^ in comparison with KPNB1 H1–8^WT^, indicating that the nuclear translocation activity of KPNB1 also depends on the FG-Nup interaction domain.

To determine the effect of KPNB1 on TDP-43 localization in the context of intact central nervous system (CNS) tissue, we employed AAV-transduction of organotypic mouse brain slice cultures (BSCs) (Fig. 6e) [30, 48]. BSCs were co-transduced with AAV-GFP-TDP-43^mNLS^ and various AAV-FLAG-KPNB1 constructs, or AAV-FLAG-mCherry as a control. Twelve days after transduction, TDP-43^mNLS^ was cytoplasmic without any obvious signs of aggregation (Fig. 6e). Expression of KPNB1 H1–9^WT^ and H1–8^WT^ strongly increased the nuclear localization of TDP-43^mNLS^ (arrowheads), whereas KPNB1 constructs carrying NIS mutations to prevent FG-Nup-binding had no effect (Fig. 6e,f). We also tested the effect of these constructs on AAV-mScarlet-TDP-CTF, which forms small cytoplasmic aggregates in this model (Supplementary Fig. 19a, arrows). Strikingly, TDP-CTF became very nuclear in presence of KPNB1 H1–9^WT^ and H1–8^WT^. This effect was less pronounced with KPNB1 H1–9^mNIS^ and completely absent with KPNB1 H1–8^mNIS^ (Supplementary Fig. 19). KPNB1 H10–19 had no effect on the localization of TDP-43^mNLS^ and TDP-CTF (Fig. 6e,f and Supplementary Fig. 19). Taken together, these data demonstrate that KPNB1 reduces TDP-43 cytoplasmic aggregation and mislocalization in an FG-Nup-dependent but NLS-independent manner, both in primary neuron and BSC models of TDP-43 proteinopathy.

### Nup62 and KPNB1 are sequestered into cytoplasmic pTDP-43 aggregates in ALS/FTD postmortem CNS tissue

**Fig. 7:**
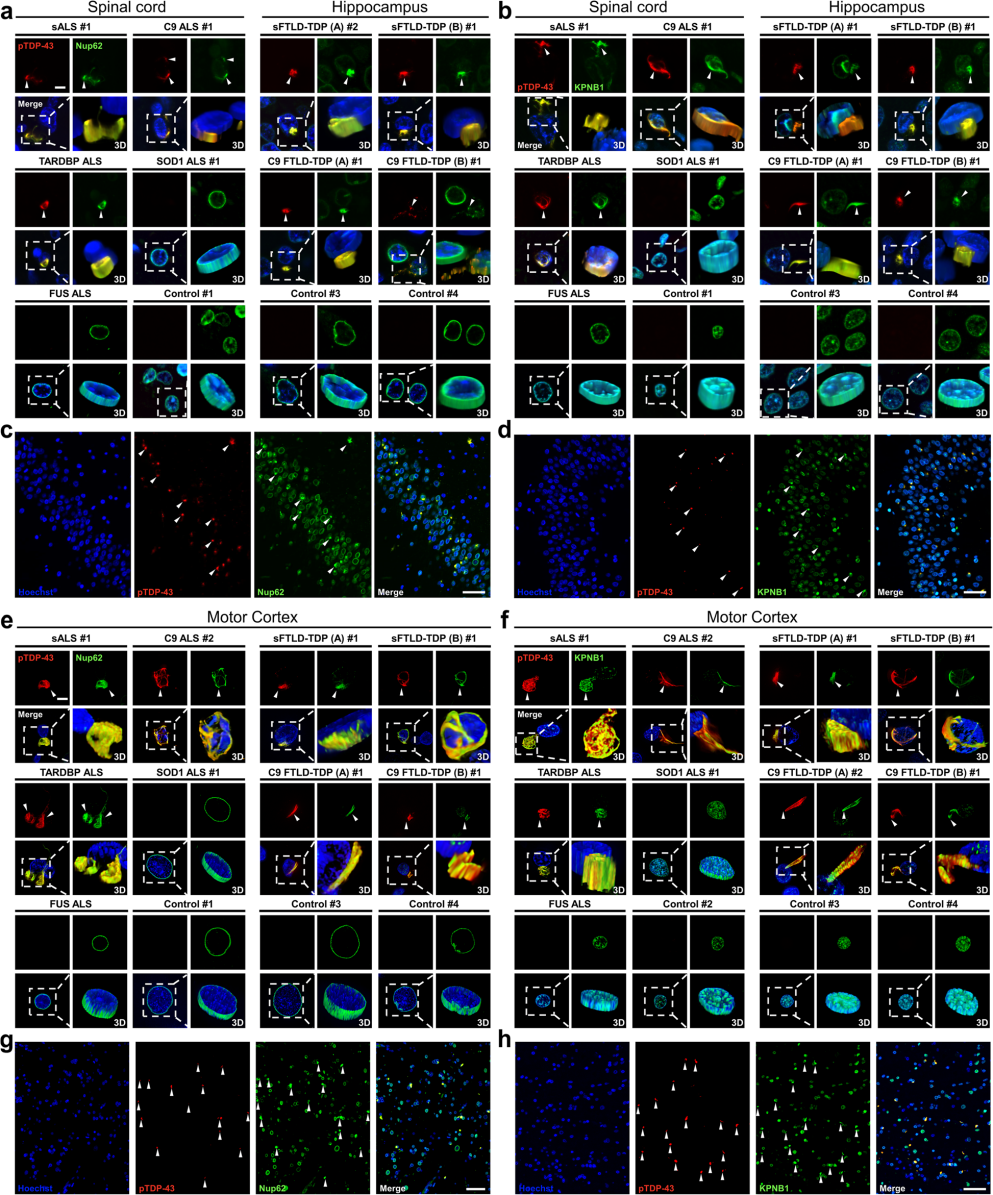
Nup62 and KPNB1 colocalize with pathological pTDP-43 aggregates in human post-mortem brain and spinal cord tissue. Co-immunofluorescence (IF) staining was conducted in fixed spinal cord, posterior hippocampus, and motor cortex of neuropathologically diagnosed ALS and FTLD cases, respectively, and including unaffected control tissue. **a,b** High resolution co-IF staining of pTDP-43 (red) and Nup62 or KPNB1 (green) with Hoechst (blue) in spinal cord (ALS) and hippocampus (FTLD). Each image is shown as a panel of projected optical sections from each z-series for red and green channels and merged with Hoechst DNA staining. Volume rendered z-series of all channels are shown as the 3D inset. Scale bar: 5 μm. **c,d** Hippocampal tile scan images of Nup62 or KPNB1 co-IF with pTDP-43 aggregates in the sFTLD-B#1 case. Scale bar: 50 μm. **e,f** High resolution co-IF staining of pTDP-43 (red) and Nup62 or KPNB1 (green) with Hoechst (blue) in the motor cortex. Scale bar: 5 μm. **g,h** Cortical tile scan images of Nup62 or KPNB1 co-IF with pTDP-43 aggregates in TARDBP ALS case. Scale bar: 50 μm

Previous studies have reported cytoplasmic mislocalization of KPNB1 in ALS cases [49–53] and Nup62 was found to colocalize with pTDP-43 aggregates in a *Drosophila* model of traumatic brain injury and human autopsy brain tissue from patients with chronic traumatic encephalopathy (CTE) and ALS/FTLD [54, 55]. To determine whether both proteins are mislocalized in pTDP-43 inclusions in ALS/FTLD, we performed Nup62 and KPNB1 co-immunostainings in autopsy tissue with pTDP-43 pathology (case demographics summarized in Supplementary Table 1). In ALS and FTLD, several CNS areas are considered to have an early predilection in the development of pTDP-43 pathological spread - such as the lower motor neurons of the spinal cord in ALS and anteromedial cortical areas including the hippocampus in FTLD [56, 57]. Although a defining and early-stage feature of ALS pathogenesis is the degeneration of primary motor cortex associated with pTDP-43 inclusions [57], this brain area is also affected in the mid-to-late disease stages of FTLD [56]. Thus, spinal cord and hippocampus were used to investigate the distribution of Nup62 or KPNB1 with pTDP-43 in ALS and FTLD, respectively, whereas motor cortex was assessed in all cases, including unaffected controls. pTDP-43 inclusions were positive for both Nup62 and KPNB1 in the spinal cord of subjects diagnosed with sporadic, C9- and mutant TARDBP-ALS (Fig. 7a,b and Supplementary Fig. 20). Nup62 was almost completely absent from the nuclear rim in these cells, while KPNB1 was still partially present in the nucleus. Similar observations were made in the hippocampus and motor cortex of sporadic and C9-FTLD-TDP cases, including FTLD-TDP pathological subtypes A and B, although Nup62 was still weakly detected on the nuclear envelope (Fig. 7). Notably, spinal cord tissue and motor cortex from subjects diagnosed with SOD1-ALS or FUS-ALS, which do not exhibit TDP-43 pathology, were devoid of cytoplasmic Nup62 and KPNB1 aggregates (Fig. 7). Neuropathologically normal control spinal cord, hippocampus, and motor cortex showed robust localization of Nup62 on the nuclear rim of the majority of cells and nucleoplasmic localization of KPNB1, in the absence of pTDP-43 pathology. Our finding that both Nup62 and KPNB1 are recruited to all pTDP-43 inclusions in ALS/FTD patients, strongly supports the relevance of our data from disease models for human TDP-43 proteinopathies.

## Discussion

The loss of nuclear TDP-43 together with its cytoplasmic accumulation and phase transition into insoluble aggregates plays a central role in the pathogenesis of the ALS/FTD disease spectrum and other TDP-43 proteinopathies [10, 58, 59]. In this study, we describe a novel non-canonical role of KPNB1 and other NIRs to restore TDP-43 solubility and nuclear localization, and thus confer neuroprotection across different in vitro, ex vivo and in vivo models of TDP-43 proteinopathy. We found that a specific subset of importins but not exportins show activity towards TDP-43, perhaps due to differences between the distribution of charged residues and the spatial organization of cargo and FG-Nup binding sites [60]. We show that mechanistically this activity depends on the NLS-independent association of KPNB1 with the PrLD of TDP-43 that is mediated by FG-Nups co-aggregating with TDP-43 in disease models and patient tissue, with both proteins serving as substrates for the molecular chaperone activity of KPNB1.

Previously, we discovered that TDP-43 pathology causes an abnormal accumulation of NPC proteins in the cytoplasm, leading to functional defects in NCT [16]. Nuclear pore defects, nucleoporin aggregation, altered nuclear morphology, and impaired nuclear transport have now been identified as a common hallmark of various age-related neurodegenerative diseases [61–67]. There is growing evidence that NIRs can regulate aberrant phase transition of RNA-binding proteins (RBPs) in ALS/FTD and other neurodegenerative diseases (reviewed by [60, 68, 69]). The first mechanistic explanation for a role of NIRs in ALS/FTD was reported when TNPO1 was found to inhibit phase separation and fibrillization of recombinant FUS by binding its PY-NLS, which is abrogated by ALS-associated FUS-NLS mutations and loss of FUS arginine methylation, as seen in FTD-FUS [18–21]. Similarly, KPNB1 together with importin-α binds the NLS of TDP-43 and reduces the fibrillization of TDP-43^WT^, but not TDP-43^mNLS^ or TDP-CTF in vitro [18, 70]. These data led to a model where the NLS acts as a key initiation signal for nuclear import, chaperoning and disaggregation of FUS and other RBPs [18–21]. However, this differs fundamentally from our present studies in cell-based assays that demonstrate that KPNB1 can reduce the cytoplasmic aggregation of TDP-43 lacking a functional NLS [16] (Fig. 1, Fig. 4 and Supplementary Fig. 1). While a recent study reported that binding of the importin-α/KPNB1 complex to the NLS prevents TDP-43 dimerization as the foundation of disaggregation, this in vitro structural analysis was limited to a recombinant soluble fragment of TDP-43 spanning residues aa1–177 that lacked the C-terminal PrLD, which was identified in the present study as the key domain for KPNB1-mediated disaggregation in the context of living cells [71, 72]. The existence of a potential additional NLS-independent mechanism to suppress phase transition of RBPs is also supported by the observation that under certain conditions TNPO1 can dissolve truncated FUS lacking the PY-NLS [19, 73] and that other NIRs, including KPNB1, can bind the RGG motifs of FUS and reduce its fibrillization [74]. Based on our findings in cellular models of TDP-43 proteinopathy, we proposed that 1) there are additional KPNB1 interaction domains present in TDP-43 and 2) additional factors missing in in vitro fibrillization studies but present in cellular TDP-43 aggregates can facilitate this process. To test this hypothesis, we undertook a comprehensive interaction mapping analysis and while we identified a small interaction domain for KPNB1 in the RRM2 (Supplementary Fig. 5), it appears that weak multivalent associations throughout the TDP-43 PrLD are required and sufficient for reducing TDP-CTF and TDP-43^mNLS^ aggregation, thus identifying the relevant KPNB1 interaction domain (Fig. 4 and Supplementary Fig. 9).

In our search for the unknown factor connecting KPNB1 with TDP-CTF aggregates, we were guided by our KPNB1 domain mapping experiments that showed an overlap between the minimal region required and sufficient for its chaperone activity towards TDP-43 aggregates and the minimal FG-Nup high-affinity binding region from HEAT repeats 1–8 (Fig. 3). While smaller KPNB1 fragments displayed some residual activity (Fig. 2d,e), they did not strongly associate with FG-Nups (Fig. 3b). We mainly focused on Nup62, which strongly co-aggregates with TDP-CTF [16] and is recruited into TDP-43 aggregates induced in a seeding model of TDP-43 proteinopathy [45]. Moreover, Nup62 is lost from the nucleus in spinal motor neurons of sporadic ALS cases [50–52] and colocalizes with pTDP-43-positive inclusions in patients with CTE and ALS/FTD [54, 55]. The finding that Nup62 can promote TDP-43 cytoplasmic mislocalization and aggregation in a fly model of traumatic brain injury and in mammalian cells [54], suggests a potential role of Nup62 as a driver in the disease process. We also found that Nup62 expression strongly increases insoluble levels of TDP-CTF and that both proteins form amyloid staining-positive insoluble aggregates (Fig. 4g and Supplementary Fig. 23). While FUS pathology has been linked to NCT defects [75], we and others have not observed any co-aggregation of pathological FUS with FG-Nups in cellular models (Supplementary Fig. 9d) or ALS/FTD patient tissue (Fig. 7) [55]. While these studies show that TDP-43 pathology can be initiated by both seeding/overexpressing cytoplasmic TDP-43 or by causing NCT defects via FG-Nups, we propose that these processes are tightly linked in a positive feedback loop, where TDP-43 mislocalization and NCT defects contribute to a vicious circle of increasing TDP-43 pathology [16].

A role for FG-Nups in this process was likely, since we demonstrated that KPNB1 mNIS constructs that only weakly bind to Nup62 and other FG-Nups, failed to reduce aggregation of cytoplasmic TDP-43 in cells (Fig. 3c-i and Fig. 6c) and brain slices (Fig. 6e and Supplementary Fig. 19a). Importantly, this was also true for recombinant TDP-CTF and Nup62FG co-aggregates in vitro (Fig. 4f,g), where WT but not mNIS KPNB1 dissolved pre-formed TDP-CTF–Nup62 co-aggregates and prevented their formation, demonstrating its molecular chaperone and disaggregation activities. Moreover, we found that KPNB1 associates specifically with the PrLD of TDP-43 via Nup62 and Nup98, and that this interaction is necessary for its aggregation-reducing activity. KPNB1 expression weakly reduced insoluble levels of sTDP, which lacks the PrLD (Fig. 4a,b), and TDP-CTF^VLIM-F^, which harbors additional phenylalanine residues in its PrLD (Supplementary Fig. 9g,h). Both TDP-43 constructs were negative for Nup62 and KPNB1 colocalization and did not show reduced aggregation, in contrast to TDP-CTF^WT^ (Fig. 4a-d and Supplementary Fig. 9a-c,f-h). While we and others speculated that FG-Nups might associate with the FG-like repeats that are present in the TDP-43 PrLD [71], here we show that TDP-CTF variants where phenylalanine residues in the PrLD were replaced with glycine, alanine, or tyrosine residues still co-aggregate with Nup62 (Supplementary Fig. 9a). In contrast, increasing the number of phenylalanine residues in the PrLD of TDP-CTF^VLIM-F^ abrogated binding to Nup62 and Nup98 (Supplementary Fig. 9a-c and Supplementary Fig. 10).

**Fig. 8:**
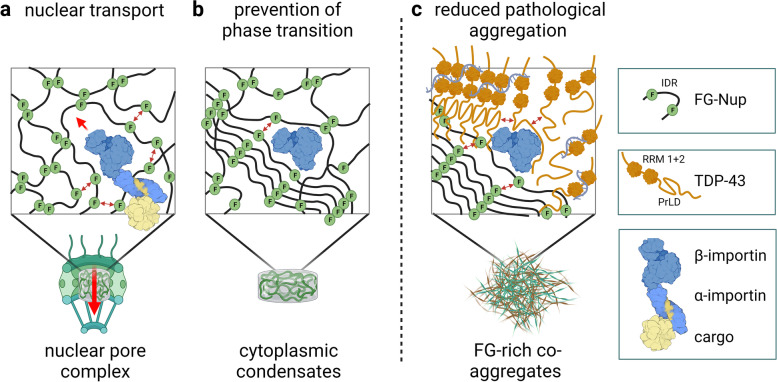
A model for a novel role of KPNB1 and other NIRs as molecular chaperones for FG-Nup-containing assemblies. **a** KPNB1 facilitates nuclear transport by disengaging hydrophobic interactions between FG-repeats that form the hydrogel barrier within nuclear pore complexes. **b** KPNB1 prevents detrimental phase transition of FG-Nups and other aggregation-prone proteins that form cytoplasmic foci under cellular crowding conditions. **c**, KPNB1 is recruited into pathological FG-Nups and TDP-43 co-aggregates in an NLS-independent manner, counters their aberrant phase transition, and relocates TDP-43 into the nucleus. Similar mechanisms may apply to other NIRs. IDR: intrinsically disordered region; RRM: RNA-recognition motif; PrLD: prion-like domain. (Created with BioRender.com)

Our findings suggest a model where KPNB1 performs its canonical role in facilitating nuclear import of cargo proteins by locally disengaging weak hydrophobic interactions between IDRs of FG-Nups that form the diffusion-blocking hydrogel in the nuclear pore [34, 76] (Fig. 8a). In addition to the transport function, NIRs may act as chaperones that prevent harmful phase transitions of aggregation-prone proteins based on charged residues or hydrophobic interactions under molecular crowding conditions in the cytoplasm (Fig. 8b). KPNB1 and other NIRs have been shown to act as chaperones that prevent the cytoplasmic aggregation of RNA/DNA-binding proteins with exposed basic domains [77, 78]. Although Nup62 is mainly present in the central channel of NPCs, it has also been detected in mobile foci with KPNB1 [79]. Under in vitro crowding conditions, FG-Nups can aggregate into amyloid fibrils. This aggregation can be inhibited by KPNB1 in vitro [76, 80], providing a biological mechanism of how cells may block and control amyloid formation of highly aggregation-prone FG-Nups in the crowded intracellular environment. Under disease conditions, TDP-43 co-aggregates in the cytoplasm with FG-Nups including Nup62, mediated via the PrLD (Fig. 8c). Our data suggest that KPNB1 is recruited by FG-Nups present in TDP-43 aggregates and makes secondary contacts with the TDP-PrLD. KPNB1 then either disengages or prevents intermolecular interactions in FG-Nups and TDP-43 PrLD fibrils, leading to soluble TDP-43 in complex with KPNB1 being imported into the nucleus, reversing all major hallmarks of TDP-43 proteinopathy. These findings are in contrast to FUS, where the PY-NLS plays a key role in TNPO1-mediated disaggregation, while here we show that the TDP-43 PrLD is essential for its KPNB1-mediated disaggregation. Of note, this difference between FUS and TDP-43 is also supported by ALS-causing mutations being clustered in the PY-NLS of FUS and the PrLD of TDP-43 [22]. While one variant in the TDP-43 NLS (A90V) has been described as a potential ALS-associated mutation [72, 81], its pathogenic potential in ALS and FTD remains unclear since it does not appear to cause pathology in cellular models and has also been found in healthy controls [82].

Several studies have reported that arginine-rich DPRs (R-DPRs) can promote TDP-43 phase separation and aggregate formation [41, 70]. These DPRs not only sequester TDP-43, but also several Nups and NIRs including Nup62 and KPNB1 [41, 70, 83]. This reinforces our hypothesis that both aggregation-prone proteins, TDP-43 and Nup62, can co-aggregate and recruit KPNB1. We found that expression of KPNB1 H1–8^WT^ abolishes the incorporation of TDP-43 and Nup62 into cytoplasmic poly(GR) aggregates in an FG-Nup-dependent fashion (Fig. 3f-i). R-DPRs have been shown to reduce the solubility of multiple NIRs and TDP-43 and impair nuclear import of TDP-43 and other NLS-bearing cargoes in vitro; in line with our findings, KPNB1 and TNPO1, but not XPO1, can prevent poly(GR)-induced precipitation of recombinant TDP-43 [70]. Thus, although R-DPRs can impede the ability of NIRs to regulate phase transition of TDP-43, this can be reversed by increased NIR levels.

KPNB1 is known to actively import TDP-43 into the nucleus via importin-α, with KPNB1 downregulation causing cytoplasmic accumulation of TDP-43 in neuronal cells [17]. Several groups have reported that KPNB1 accumulates in the cytoplasm of spinal motor neurons of sporadic and C9-ALS patients [49–53], although the relationship to TDP-43 inclusions was not addressed. Here, we show robust evidence from several CNS areas that KPNB1 accumulates in pTDP-43 inclusions consistently in various sporadic and genetically linked ALS and FTLD autopsy tissues (Fig. 7). Inversely, RNA-seq data analysis of sporadic ALS spinal cords revealed a significant downregulation of KPNB1 [84], suggesting that cytoplasmic mislocalization or reduced levels of NIRs in ALS/FTD can impede TDP-43 nuclear import and thus facilitate its pathological mislocalization. Accordingly, we found that overexpression of Ketel, the fly homologue of KPNB1, alleviates toxicity of mutant TDP-43 in *Drosophila* models (Fig. 5a-d, Supplementary Fig. 15 and Supplementary Fig. 16). To rule out a toxic effect of KPNB1 overexpression in our experiments due to increased import of NLS-containing proteins or sequestration of Ran, we examined the effect of KPNB1 constructs on nuclear import of the fluorescent reporter construct NES-tdTomato-NLS and on the localization of endogenous Ran in SH-SY5Y cells. Full-length and N-terminal KPNB1 did not alter the N-to-C ratio of the reporter construct, nor affect the localization of Ran (Supplementary Fig. 21). Importantly, KPNB1 overexpression did not appear to affect expression, solubility, or localization of physiologically normal TDP-43^WT^ and endogenous TDP-43 in mammalian cells and mouse brain neurons, indicating a specific effect on pathological TDP-43 variants (Supplementary Fig. 1 and Supplementary Fig. 22). These findings suggest that enhancing expression or activity of KPNB1 (or other NIRs) may be a valid therapeutic intervention to target TDP-43 proteinopathies [85].

## Conclusions

Here we demonstrate that KPNB1 can rescue TDP-43 proteinopathy and neurotoxicity across different models of TDP-43 pathology. Similar to its canonical role in disengaging hydrophobic interactions between hydrogel-forming FG-Nups in the NPC to facilitate nuclear import, KPNB1 has a non-canonical role in chaperoning cytoplasmic FG-Nups and RNA/DNA-binding proteins. We propose that under disease conditions, KPNB1 is recruited to pathological TDP-43/FG-Nup co-aggregates, where it can dissociate multivalent interactions between FG-Nups and TDP-43 PrLD fibrils, thus allowing TDP-43 to be imported back to the nucleus where it can resume its physiological functions in splicing regulation. Unlike previous studies with recombinant TDP-43 and FUS, this activity is NLS-independent in living cells. Since we found that KPNB1 is abnormally sequestered into pTDP-43 inclusions in ALS/FTD patient tissue, we conclude that increasing expression or activity of KPNB1 may be therapeutical in restoring normal localization and function of TDP-43, thus mitigating neurodegeneration.

## Supplementary Information

**Additional file 1: Supplementary Fig. 1.** KPNB1 specifically reduces insoluble protein levels of TDP-CTF. a, (Top) Immunofluorescence (IF) of SH-SY5Y cells co-expressing mCherry-TDP-CTF with GFP or GFP-KPNB1. mCherry-TDP-CTF forms cytoplasmic aggregates but shows diffuse localization in the presence of GFP-KPNB1. Hoechst staining was used to outline nuclei. Scale bar: 5 μm. (Bottom) Western blot analysis and quantification of insoluble mCherry-TDP-CTF and endogenous TDP-43 protein levels in SH-SY5Y cells expressing GFP or GFP-KPNB1. KPNB1 significantly reduced insoluble TDP-CTF levels, while endogenous insoluble TDP-43 levels were unaffected. β-tubulin was used as a loading control. Statistical analysis was performed using two-sided Student’s *t*-test (***p < 0.001, *n* = 10). b, Western blot analysis of soluble and insoluble GFP-TDP-CTF co-expressed with mCherry, mCherry-KPNB1 or untagged KPNB1 in HEK293T cells. KPNB1 only reduces insoluble TDP-CTF levels. β-tubulin was used as a loading control. c, IF of HEK293T cells co-expressing GFP-TDP-CTF or GFP-TDP-43^mNLS^ with mCherry or mCherry-KPNB1. TDP-CTF and TDP-43^mNLS^ form cytoplasmic aggregates positive for phospho-TDP-43^S409/410^ but show diffuse localization with no pathological hyperphosphorylation in the presence of mCherry-KPNB1. Arrowheads point to colocalization. Scale bar: 5 μm. d, Western blot analysis and quantification of insoluble GFP-tagged TDP-CTF^WT^, TDP-CTF^Q331K^, TDP-CTF^M337V^ and TDP-CTF^A382T^ in HEK293T cells expressing mCherry or mCherry-KPNB1. KPNB1 similarly reduced insoluble wild-type and ALS-derived mutant TDP-CTF protein levels. β-tubulin was used as a loading control. Statistical analysis was performed using two-sided Student’s *t*-test (***p < 0.001, *n* = 3). e, Western blot analysis of total protein levels of GFP-tagged TDP-CTF or TDP-43 in HEK293T cells expressing mCherry, mCherry-KPNB1 or untagged KPNB1. Cells were lysed in 7 M urea buffer to collect total protein. KPNB1 reduces total protein levels of TDP-CTF, but not full-length TDP-43. β-tubulin was used as a loading control. Statistical analysis was performed using one-way ANOVA and Bonferroni’s post hoc test (*p < 0.05, ***p < 0.001, *n* = 4). f, *TDP-CTF* and *TDP-43* transcript levels were quantified in HEK293T cells co-expressing GFP-tagged TDP-CTF or TDP-43 with mCherry, mCherry-KPNB1, or untagged KPNB1. *GAPDH* was used as a reference to normalize the transcript levels. Statistical analysis was performed using one-way ANOVA and Bonferroni’s post hoc test (*n* = 3). **Supplementary Fig. 2.** Expression of full-length KPNB1 and its N-terminal half do not reduce endogenous insoluble TDP-43 levels. Quantification of endogenous insoluble TDP-43 protein levels in SH-SY5Y cells co-expressing mCherry-TDP-CTF with GFP, GFP-KPNB1 full-length (FL), H1–9 or H10–19. β-tubulin was used as a loading control. Statistical analysis was performed using one-way ANOVA and Bonferroni’s post hoc test (n = 4). **Supplementary Fig. 3.** Cytoplasmic GFP-(GR)_100_ aggregates sequester endogenous TDP-43 and Nup62. a, Immunofluorescence of HEK293T cells expressing GFP-(GR)_100_ and stained for endogenous TDP-43 and Nup62. Only cytoplasmic GFP-(GR)_100_ aggregates are immunopositive for TDP-43 and Nup62. White arrowheads point to cytoplasmic poly(GR) aggregates. Nuclear GFP-(GR)_100_ aggregates render TDP-43 staining diffuse (purple arrowhead), while Nup62 accumulates as puncta in the cytoplasm (red arrowhead). Hoechst staining was used to outline nuclei. Scale bar: 5 μm. b, Quantification of the percentage of cytoplasmic GFP-(GR)_100_ aggregates positive for TDP-43 or Nup62 in HEK293T cells. Almost all cells are positive for both proteins. Statistical analysis was performed using two-sided Student’s *t*-test (three independent experiments; *n* = 239–249 cells per group). **Supplementary Fig. 4.** Untagged KPNB1 interacts with both the RRM2 and PrLD of TDP-CTF. a, (Left) Lysates from HEK293T cells expressing GFP alone, or untagged KPNB1 with GFP, GFP-TDP-CTF or GFP-TDP-43 were subjected to immunoprecipitation with GFP-Trap magnetic beads. Whole cell lysates (input) and immunoprecipitates (IP) were subjected to western blot analysis using indicated antibodies. (Right) Quantification of relative KPNB1 levels in IP normalized by KPNB1 levels in input. Statistical analysis was performed using one-way ANOVA and Bonferroni’s post hoc test (**p < 0.01, ***p < 0.001, *n* = 3). b, Schematic domain structure of TDP-CTF full-length (208–414). TDP-CTF contains part of the RRM2 (non-PrLD, 208–274) and the C-terminal PrLD (275–414). c, (Left) Lysates from HEK293T cells expressing GFP alone, or untagged KPNB1 with GFP, GFP-TDP-CTF, GFP-TDP-CTF^non-PrLD^ or GFP-TDP-PrLD were subjected to immunoprecipitation with GFP-Trap magnetic beads. Whole cell lysates (input) and immunoprecipitates (IP) were subjected to western blot analysis using indicated antibodies. (Right) Quantification of relative KPNB1 levels in IP normalized by KPNB1 levels in input. Statistical analysis was performed using one-way ANOVA and Bonferroni’s post hoc test (*p < 0.05, **p < 0.01, n = 3). *cleavage products. **Supplementary Fig. 5.** KPNB1 reduces TDP-CTF aggregation independent of its interaction with the RRM2 region. a, Schematic domain structures of full-length TDP-CTF (208–414), and its C-terminal and N-terminal deletion constructs used for co-IP experiments. b, Lysates from HEK293T cells co-expressing mCherry-KPNB1 with GFP, GFP-TDP-CTF or GFP-TDP-CTF C-terminal deletion constructs were subjected to immunoprecipitation with RFP-Trap magnetic beads. Whole cell lysates (input) and immunoprecipitates (IP) were subjected to western blot analysis using indicated antibodies. Deletion of amino-acids 230–240 in TDP-CTF reduced its binding to KPNB1. c, Lysates from HEK293T cells co-expressing mCherry-KPNB1 with GFP, GFP-TDP-CTF or GFP-TDP-CTF N-terminal deletion constructs were subjected to immunoprecipitation with RFP-Trap magnetic beads. Whole cell lysates (input) and immunoprecipitates (IP) were subjected to western blot analysis using indicated antibodies. Deletion of amino-acids 230–240 in TDP-CTF reduces its binding to KPNB1. TDP-PrLD weakly interacts with KPNB1. d, (Left) Lysates from HEK293T cells co-expressing mCherry-KPNB1 with GFP, GFP-TDP-CTF^WT^ or GFP-TDP-CTF^Δ230–240^ were subjected to immunoprecipitation with RFP-Trap magnetic beads. Whole cell lysates (input) and immunoprecipitates (IP) were subjected to western blot analysis using indicated antibodies. KPNB1 interacts less with TDP-CTF^Δ230–240^. (Right) Quantification of relative GFP levels in IP normalized by GFP levels in input confirm that GFP-TDP-CTF^Δ230–240^ interacts less with mCherry-KPNB1. Statistical analysis was performed using two-sided Student’s *t*-test (**p < 0.01, n = 3). e, Immunofluorescence of HEK293T cells co-expressing GFP-tagged TDP-CTF^WT^ or TDP-CTF^Δ230–240^ with mCherry or mCherry-KPNB1. TDP-CTF^Δ230–240^ forms small nuclear and big cytoplasmic aggregates. KPNB1 reduces both TDP-CTF^WT^ and TDP-CTF^Δ230–240^ aggregates and makes TDP-CTF^Δ230–240^ more nuclear. Hoechst staining was used to outline nuclei. Scale bar: 5 μm. f, Western blot analysis and quantification of insoluble GFP-tagged TDP-CTF^WT^ or TDP-CTF^Δ230–240^ in HEK293T cells expressing mCherry or mCherry-KPNB1. KPNB1 equally reduced insoluble TDP-CTF^WT^ and TDP-CTF^Δ230–240^ protein levels. β-tubulin was used as a loading control. Statistical analysis was performed using two-way ANOVA and Bonferroni’s post hoc test (***p < 0.001, n = 3). **Supplementary Fig. 6.** The PrLD of TDP-43 interacts with FG-Nups and KPNB1. a, Immunofluorescence (IF) of HEK293T cells expressing GFP or GFP-TDP-PrLD and stained for endogenous Nup98 and Nup62. TDP-PrLD forms small cytoplasmic foci positive for Nup62. Hoechst staining was used to outline nuclei. Scale bar: 5 μm. b, IF of HEK293T cells expressing GFP or GFP-TDP-PrLD and stained for endogenous KPNB1. KPNB1 colocalizes with TDP-PrLD foci in the cytoplasm. Hoechst staining was used to outline nuclei. Scale bar: 5 μm. c, Lysates from HEK293T cells expressing GFP or GFP-TDP-PrLD were subjected to immunoprecipitation with GFP-Trap magnetic beads. Whole cell lysates (input) and immunoprecipitates (IP) were subjected to western blot analysis using indicated antibodies. The PrLD of TDP-43 interacts with Nup62, Nup98 and KPNB1, but not with Ran. **Supplementary Fig. 7.** Colocalization of Nup62 with TDP-43 constructs depends on the PrLD. Colocalization between Nup62-mCherry and GFP or GFP-tagged TDP-43 constructs was measured using the Mander’s overlap coefficient. Values close to 0 and 1 indicate a weak and strong colocalization, respectively. Statistical analysis was performed using one-way ANOVA and Bonferroni’s post hoc test (***p < 0.001, *n* = 21–23 cells per group). **Supplementary Fig. 8.** The NES of sTDP does not prevent it from binding to Nup62 aggregates. Immunofluorescence of HEK293T cells co-expressing Nup62-mCherry with either sTDP or sTDP^mNLS^ ΔNES. Nup62 aggregates do not colocalize with sTDP even after mutating its NLS and NES. Hoechst staining was used to outline nuclei. Scale bar: 5 μm. **Supplementary Fig. 9.** TDP-CTF PrLD mutations that reduce its association with Nup62 abrogate its interaction and reduced aggregation by KPNB1. a, Immunofluorescence (IF) of HEK293T cells co-expressing Nup62-mCherry with either GFP-tagged TDP-CTF^WT^ or TDP-CTF constructs harboring different mutations in the PrLD. All TDP-CTF mutant variants colocalize with Nup62 aggregates, except for TDP-CTF^VLIM-F^ (green arrowhead) which accumulates in close proximity to Nup62 (red arrowhead). Scale bar: 5 μm. b, Colocalization between Nup62-mCherry and GFP-tagged TDP-CTF^WT^ or TDP-CTF^VLIM-F^ was measured using the Mander’s overlap coefficient. Values close to 0 and 1 indicate a weak and strong colocalization, respectively. Statistical analysis was performed using two-sided Student’s *t*-test (***p < 0.001, n = 21–22 cells per group). c, IF of HEK293T cells co-expressing Nup62-mCherry with either GFP-tagged wild-type or mutant TDP-43^mNLS^ constructs. RNA-binding deficient TDP-43^mNLS^ (TDP-43^mNLS^ 5F-L) can still colocalize with Nup62. Only TDP-43^mNLS^ VLIM-F aggregates (green arrowhead) do not contain Nup62 (red arrowhead). Scale bar: 5 μm. d, IF of HEK293T cells co-expressing Nup62-mCherry with GFP-FUSΔ14. Cytoplasmic FUS aggregates (green arrowhead) do not colocalize with Nup62 (red arrowhead). Scale bar: 5 μm. e, Protein sequences of TDP-CTF^WT^ and TDP-CTF^VLIM-F^ showing FG repeats (underlined) and VLIM-F mutations (in red) in the PrLD. f, (Left) Lysates from HEK293T cells co-expressing mCherry-KPNB1 with GFP, GFP-tagged TDP-CTF^WT^ or TDP-CTF^VLIM-F^ were subjected to immunoprecipitation with RFP-Trap magnetic beads. Whole cell lysates (input) and immunoprecipitates (IP) were subjected to western blot analysis using indicated antibodies. Introducing VLIM-F mutations in TDP-CTF significantly reduces its binding to KPNB1. (Right) Relative GFP levels in IP normalized by GFP levels in input confirm that GFP-TDP-CTF^VLIM-F^ interacts less with mCherry-KPNB1. Statistical analysis was performed using two-sided Student’s *t*-test (*p < 0.05, *n* = 3). g, IF of HEK293T cells co-expressing GFP-tagged TDP-CTF^WT^ or TDP-CTF^VLIM-F^ with mCherry or mCherry-KPNB1. KPNB1 only reduces the size of TDP-CTF^VLIM-F^ aggregates. Hoechst staining was used to outline nuclei. Scale bar: 5 μm. h, Western blot analysis and quantification of insoluble GFP-tagged TDP-CTF^WT^ or TDP-CTF^VLIM-F^ in HEK293T cells expressing mCherry or mCherry-KPNB1. KPNB1 strongly decreased protein levels of insoluble TDP-CTF^WT^ but not TDP-CTF^VLIM-F^. β-tubulin was used as a loading control. Statistical analysis was performed using two-way ANOVA and Bonferroni’s post hoc test (***p < 0.001, *n* = 4). **Supplementary Fig. 10.** Nup98 strongly associates with TDP-CTF^WT^, but not TDP-CTF^VLIM-F^ and sTDP. Immunofluorescence of HEK293T cells co-expressing GFP-Nup98 with mCherry or mCherry-tagged TDP-43^mNLS^, TDP-43^mNLS^ 1–265, TDP-PrLD, sTDP, TDP-CTF^WT^ or TDP-CTF^VLIM-F^. Arrowheads point to co-aggregation. Hoechst staining was used to outline nuclei. Scale bar: 5 μm. **Supplementary Fig. 11.** KPNB1 H1–8 reduces cytoplasmic Nup62 aggregates. Immunofluorescence of HEK293T cells co-expressing Nup62-GFP with mCherry, mCherry-tagged KPNB1 H1–8^WT^ or H1–8^mNIS^. KPNB1 H1–8^WT^ suppresses Nup62 aggregates, whereas NIS mutations abolished this activity. Hoechst staining was used to outline nuclei. Scale bar: 5 μm. **Supplementary Fig. 12.** Multiple Ketel RNAi lines enhance hTDP-43^M337V^ toxicity in the fly eye when expressed under the control of the Gmr-Gal4 driver. **Supplementary Fig. 13.** Ketel overexpression does not change total levels of human TDP-43 protein in flies. Western blot analysis of hTDP-43 expression in fly brains. hTDP-43 is expressed upon administration of RU486, and Ketel overexpression had no effect on total protein levels of TDP-43. β-actin was used as a loading control. Statistical analysis was performed using one-way ANOVA and Sidak’s post hoc test (n = 3). **Supplementary Fig. 14.** Survival *Kaplan-Meier* curves of Elav-GS flies carrying the indicated transgenes in the absence of RU486. Note that all the negative controls yield similar results. **Supplementary Fig. 15.** The EP-Ketel insertion P{EPgy2}Fs(2)Ket^EY06666^ rescues mutant hTDP-43 toxicity in flies. a, EP-Ketel suppresses TDP-43^M337V^ toxicity in the *Drosophila* eye. b, EP-Ketel reduces phospho-TDP-43 staining in the adult brain when co-expressed using the Elav-GS driver and exposed to RU486. c, RU486-treated flies carrying the Elav-GS driver, mutant TDP-43 and EP-Ketel (green line) live longer compared to flies carrying the control LacZ transgene (red line). The blue line corresponds to LacZ; Elav-GS Gal4 flies without drug (solvent only) as a negative control. Survival analysis was performed using the OASIS online tool, and *p*-values were calculated using Fisher’s exact test (*n* = 100, ****p < 0.0001 for all the genotypes). **Supplementary Fig. 16.** Downregulation of Ketel exacerbates toxicity of hTDP-43^ΔNLS^ in the fly eye, but Ketel overexpression alleviates it. a, Eye phenotypes from flies expressing the indicated transgenes via the eye-specific Gmr-Gal4 driver. Arrow points to necrotic areas. b, Quantitative analysis of eye severity in flies co-expressing hTDP-43^ΔNLS^ and the indicated transgenes via the eye-specific Gmr-Gal4 driver. Statistical analysis was performed using one-way ANOVA and Bonferroni’s post hoc test (***p < 0.001, *n* = 5). **Supplementary Fig. 17.** The NIR cdm/IPO13 rescues severe eye degeneration in hTDP-43^ΔNLS^ flies. a, Eye phenotypes from flies expressing the indicated transgenes via the eye-specific Gmr-Gal4 driver. Cdm overexpression (OE) rescues hTDP-43-induced neurodegeneration. b, Quantitative analysis of eye severity in flies co-expressing hTDP-43^ΔNLS^ and the indicated transgenes via the eye-specific Gmr-Gal4 driver. Statistical analysis was performed using one-way ANOVA and Bonferroni’s post hoc test (*p < 0.05, ***p < 0.001, *n* = 16–23 flies per group). **Supplementary Fig. 18.** KPNB1 increases the N-to-C ratio of different TDP-43 constructs in rodent primary neurons. Quantitative analysis of the N-to-C ratio of GFP or GFP-tagged TDP-43^WT^, TDP-43^mNLS^ or TDP-CTF in primary neurons expressing mScarlet or mScarlet-KPNB1 24 h (a) or 48 h (b) post-transfection. Statistical analysis was performed using one-way ANOVA and Bonferroni’s post hoc test (***p < 0.001, *n* = 52–264 neurons per group). **Supplementary Fig. 19.** KPNB1 reduces cytoplasmic aggregation of TDP-CTF and promotes nuclear localization in BSCs. a, Immunofluorescence of mouse BSCs (DIV12) co-expressing AAV-mScarlet-TDP-CTF with AAV-GFP, AAV-GFP-KPNB1 H1–9^WT^, AAV-FLAG-KPNB1 H1–9^WT^, H1–9^mNIS^, H1–8^WT^, H1–8^mNIS^ or H10–19. KPNB1 H1–9^WT^ and H1–8^WT^ reduce TDP-CTF cytoplasmic aggregates (arrows) and render it very nuclear (arrowheads), whereas NIS mutations strongly reduce this function. Hoechst staining was used to outline nuclei. Scale bar: 50 μm. b, Quantification of the percentage of neurons exhibiting nuclear (N), nucleocytoplasmic (NC) or cytoplasmic (C) mScarlet-TDP-CTF distribution upon expression of different AAV-KPNB1 constructs. Statistical analysis was performed using two-way ANOVA and Bonferroni’s post hoc test (three independent experiments; *n* = 150–178 neurons per group; statistics are summarized in Supplementary Table 2). **Supplementary Fig. 20.** Nup62 colocalizes with pTDP-43 aggregates in the spinal cord of TARDBP ALS case. High resolution co-immunofluorescence staining of pTDP-43 (magenta) and Nup62 (green) with Hoechst (blue) was conducted in fixed spinal cord of a neuropathologically diagnosed TARDBP ALS case. pTDP-43 was stained with a 647/Cy5 secondary antibody and Nup62 with a 488/FITC antibody to ensure there is no bleed through between channels. The image is shown as a panel of projected optical sections from each z-series for magenta and green channels and merged with Hoechst DNA staining. Volume rendered z-series of all channels are shown as the 3D inset. Scale bar: 5 μm. **Supplementary Fig. 21.** KPNB1 expression does not dysregulate nucleocytoplasmic transport. a, Immunofluorescence (IF) of SH-SY5Y cells co-expressing the transport reporter NES-tdTomato-NLS with GFP or GFP-tagged KPNB1 full-length (FL), H1–9 or H1–8. Hoechst staining was used to outline nuclei. Scale bar: 5 μm. b, Quantification of the N-to-C ratio of the NES-tdTomato-NLS reporter. Statistical analysis was performed using one-way ANOVA and Bonferroni’s post hoc test (*p < 0.05, *n* = 21–29 cells per group). c, IF of SH-SY5Y cells expressing GFP or GFP-tagged full-length KPNB1 (FL), H1–9 or H1–8 and stained for endogenous Ran and Nup98. Both proteins remain mostly nuclear in presence of KPNB1. Hoechst staining was used to outline nuclei. Scale bar: 5 μm. **Supplementary Fig. 22.** Expression of the N-terminal half of KPNB1 does not affect the nuclear localization of endogenous TDP-43 in cells and BSCs. a, Immunofluorescence (IF) of HEK293T cells co-expressing GFP with an empty plasmid (ctrl), FLAG-tagged KPNB1 H1–8^WT^ or H1–8^mNIS^ and stained for endogenous TDP-43. TDP-43 remains nuclear in presence of KPNB1 H1–8. Scale bar: 5 μm. b, IF of mouse BSCs (DIV15) expressing AAV-FLAG-KPNB1 H1–9 and stained for endogenous TDP-43. TDP-43 remains nuclear in presence of KPNB1 H1–9. Scale bar: 25 μm. **Supplementary Fig. 23.** Nup62 and TDP-CTF aggregates are positive for amyloid staining and their co-expression promotes TDP-CTF aggregation. a, Immunofluorescence of HEK293T cells expressing mCherry-TDP-CTF or Nup62-mCherry which form Amylo-Glo-positive aggregates. DRAQ5 staining was used to outline nuclei. Scale bar: 5 μm. b, Western blot analysis of insoluble mCherry-TDP-CTF in HEK293T cells expressing GFP or Nup62-GFP. Nup62 strongly increases insoluble levels of TDP-CTF. **Supplementary Table 1.** Demographic information of human post-mortem cases used for Nup62 and KPNB1 co-immunostaining with pTDP-43^S409/410^. **Supplementary Table 2.** Summary of statistical analyses. **Supplementary Table 3.** Summary of DNA constructs and sources. **Supplementary Table 4.** Summary of *Drosophila* strains and sources. **Supplementary Table 5.** Summary of reagents and sources.

## Data Availability

All data generated or analyzed during this study are included in this published article and its supplementary information.

## Abbreviations

AAV: Adeno-associated virus
AD: Alzheimer’s disease
ALS: Amyotrophic lateral sclerosis
BSA: Bovine serum albumin
BSC: Brain slice culture
CNS: Central nervous system
CTE: Chronic traumatic encephalopathy
FG-Nup: Phenylalanine and glycine-rich nucleoporin
FL: Full-length
FTD: Frontotemporal dementia
FTLD: Frontotemporal lobar degeneration
FUS: Fused in sarcoma
HD: Huntington’s disease
HEAT: Huntingtin, elongation factor 3 (EF3), protein phosphatase 2A (PP2A), TOR1 kinase
IDR: Intrinsically disordered region
IPO: Importin
KPNB1: Karyopherin-β1
NCT: Nucleocytoplasmic transport
NES: Nuclear export signal
NIR: Nuclear import receptor
NIS: Nucleoporin-interacting site
NLS: Nuclear localization signal
NPC: Nuclear pore complex
N-to-C: Nuclear-to-cytoplasmic
PrLD: Prion-like domain
pTDP-43: Phospho-TDP-43^S409/410^
RanBP: Ran-binding protein
RBP: RNA-binding protein
R-DPR: Arginine-rich dipeptide repeat protein
RRM: RNA-recognition motif
sTDP: Short-TDP-43 isoform
TDP-43: TAR DNA-binding protein-43
TDP-CTF: TDP-43 C-terminal fragment
TNPO1: Transportin-1
WT: Wild type
XPO: Exportin

## References

[1] Neumann M, Sampathu DM, Kwong LK, Truax AC, Micsenyi MC, Chou TT (2006). Ubiquitinated TDP-43 in frontotemporal lobar degeneration and amyotrophic lateral sclerosis. Science.

[2] Arai T, Hasegawa M, Akiyama H, Ikeda K, Nonaka T, Mori H (2006). TDP-43 is a component of ubiquitin-positive tau-negative inclusions in frontotemporal lobar degeneration and amyotrophic lateral sclerosis. Biochem Biophys Res Commun.

[3] Prasad A, Bharathi V, Sivalingam V, Girdhar A, Patel BK (2019). Molecular mechanisms of TDP-43 Misfolding and pathology in amyotrophic lateral sclerosis. Front Mol Neurosci.

[4] Ling SC, Polymenidou M, Cleveland DW (2013). Converging mechanisms in ALS and FTD: disrupted RNA and protein homeostasis. Neuron.

[5] Nelson PT, Dickson DW, Trojanowski JQ, Jack CR, Boyle PA, Arfanakis K (2019). Limbic-predominant age-related TDP-43 encephalopathy (LATE): consensus working group report. Brain.

[6] Amador-Ortiz C, Lin WL, Ahmed Z, Personett D, Davies P, Duara R (2007). TDP-43 immunoreactivity in hippocampal sclerosis and Alzheimer’s disease. Ann Neurol.

[7] Fallini C, Bassell GJ, Rossoll W (2012). The ALS disease protein TDP-43 is actively transported in motor neuron axons and regulates axon outgrowth. Hum Mol Genet.

[8] Colombrita C, Zennaro E, Fallini C, Weber M, Sommacal A, Buratti E (2009). TDP-43 is recruited to stress granules in conditions of oxidative insult. J Neurochem.

[9] Khalil B, Morderer D, Price PL, Liu F, Rossoll W (2018). mRNP assembly, axonal transport, and local translation in neurodegenerative diseases. Brain Res.

[10] Suk TR, Rousseaux MWC (2020). The role of TDP-43 mislocalization in amyotrophic lateral sclerosis. Mol Neurodegener.

[11] Melamed Z, Lopez-Erauskin J, Baughn MW, Zhang O, Drenner K, Sun Y (2019). Premature polyadenylation-mediated loss of stathmin-2 is a hallmark of TDP-43-dependent neurodegeneration. Nat Neurosci.

[12] Klim JR, Williams LA, Limone F, Guerra San Juan I, Davis-Dusenbery BN, Mordes DA (2019). ALS-implicated protein TDP-43 sustains levels of STMN2, a mediator of motor neuron growth and repair. Nat Neurosci.

[13] Brown AL, Wilkins OG, Keuss MJ, Hill SE, Zanovello M, Lee WC (2022). TDP-43 loss and ALS-risk SNPs drive mis-splicing and depletion of UNC13A. Nature.

[14] Ma XR, Prudencio M, Koike Y, Vatsavayai SC, Kim G, Harbinski F (2022). TDP-43 represses cryptic exon inclusion in the FTD-ALS gene UNC13A. Nature.

[15] Fallini C, Khalil B, Smith CL, Rossoll W (2020). Traffic jam at the nuclear pore: all roads lead to nucleocytoplasmic transport defects in ALS/FTD. Neurobiol Dis.

[16] Chou CC, Zhang Y, Umoh ME, Vaughan SW, Lorenzini I, Liu F (2018). TDP-43 pathology disrupts nuclear pore complexes and nucleocytoplasmic transport in ALS/FTD. Nat Neurosci.

[17] Nishimura AL, Zupunski V, Troakes C, Kathe C, Fratta P, Howell M (2010). Nuclear import impairment causes cytoplasmic trans-activation response DNA-binding protein accumulation and is associated with frontotemporal lobar degeneration. Brain.

[18] Guo L, Kim HJ, Wang H, Monaghan J, Freyermuth F, Sung JC (2018). Nuclear-import receptors reverse aberrant phase transitions of RNA-binding proteins with prion-like domains. Cell.

[19] Yoshizawa T, Ali R, Jiou J, Fung HYJ, Burke KA, Kim SJ (2018). Nuclear import receptor inhibits phase separation of FUS through binding to multiple sites. Cell.

[20] Hofweber M, Hutten S, Bourgeois B, Spreitzer E, Niedner-Boblenz A, Schifferer M (2018). Phase separation of FUS is suppressed by its nuclear import receptor and arginine methylation. Cell.

[21] Qamar S, Wang G, Randle SJ, Ruggeri FS, Varela JA, Lin JQ (2018). FUS phase separation is modulated by a molecular chaperone and methylation of arginine Cation-pi interactions. Cell.

[22] Lagier-Tourenne C, Cleveland DW (2009). Rethinking ALS: the FUS about TDP-43. Cell.

[23] Bolte S, Cordelieres FP (2006). A guided tour into subcellular colocalization analysis in light microscopy. J Microsc.

[24] Konishi HA, Yoshimura SH (2020). Interactions between non-structured domains of FG- and non-FG-nucleoporins coordinate the ordered assembly of the nuclear pore complex in mitosis. FASEB J.

[25] Ritson GP, Custer SK, Freibaum BD, Guinto JB, Geffel D, Moore J (2010). TDP-43 mediates degeneration in a novel Drosophila model of disease caused by mutations in VCP/p97. J Neurosci.

[26] Fernandez-Funez P, Sanchez-Garcia J, de Mena L, Zhang Y, Levites Y, Khare S (2016). Holdase activity of secreted Hsp70 masks amyloid-beta42 neurotoxicity in Drosophila. Proc Natl Acad Sci U S A.

[27] Singh MD, Jensen M, Lasser M, Huber E, Yusuff T, Pizzo L (2020). NCBP2 modulates neurodevelopmental defects of the 3q29 deletion in Drosophila and Xenopus laevis models. PLoS Genet.

[28] Yang JS, Nam HJ, Seo M, Han SK, Choi Y, Nam HG (2011). OASIS: online application for the survival analysis of lifespan assays performed in aging research. PLoS One.

[29] Xu F, Kula-Eversole E, Iwanaszko M, Hutchison AL, Dinner A, Allada R (2019). Circadian clocks function in concert with heat shock organizing protein to modulate mutant Huntingtin aggregation and toxicity. Cell Rep.

[30] Croft CL, Cruz PE, Ryu DH, Ceballos-Diaz C, Strang KH, Woody BM (2019). rAAV-based brain slice culture models of Alzheimer's and Parkinson's disease inclusion pathologies. J Exp Med.

[31] Igaz LM, Kwong LK, Xu Y, Truax AC, Uryu K, Neumann M (2008). Enrichment of C-terminal fragments in TAR DNA-binding protein-43 cytoplasmic inclusions in brain but not in spinal cord of frontotemporal lobar degeneration and amyotrophic lateral sclerosis. Am J Pathol.

[32] Igaz LM, Kwong LK, Chen-Plotkin A, Winton MJ, Unger TL, Xu Y (2009). Expression of TDP-43 C-terminal fragments in vitro recapitulates pathological features of TDP-43 Proteinopathies. J Biol Chem.

[33] Chou CC, Alexeeva OM, Yamada S, Pribadi A, Zhang Y, Mo B (2015). PABPN1 suppresses TDP-43 toxicity in ALS disease models. Hum Mol Genet.

[34] Yoshimura SH, Hirano T (2016). HEAT repeats - versatile arrays of amphiphilic helices working in crowded environments?. J Cell Sci.

[35] Chi NC, Adam SA (1997). Functional domains in nuclear import factor p97 for binding the nuclear localization sequence receptor and the nuclear pore. Mol Biol Cell.

[36] Percipalle P, Clarkson WD, Kent HM, Rhodes D, Stewart M (1997). Molecular interactions between the importin alpha/beta heterodimer and proteins involved in vertebrate nuclear protein import. J Mol Biol.

[37] Cingolani G, Petosa C, Weis K, Muller CW (1999). Structure of importin-beta bound to the IBB domain of importin-alpha. Nature.

[38] Bednenko J, Cingolani G, Gerace L (2003). Importin beta contains a COOH-terminal nucleoporin binding region important for nuclear transport. J Cell Biol.

[39] Otsuka S, Iwasaka S, Yoneda Y, Takeyasu K, Yoshimura SH (2008). Individual binding pockets of importin-beta for FG-nucleoporins have different binding properties and different sensitivities to RanGTP. Proc Natl Acad Sci U S A.

[40] Chew J, Cook C, Gendron TF, Jansen-West K, Del Rosso G, Daughrity LM (2019). Aberrant deposition of stress granule-resident proteins linked to C9orf72-associated TDP-43 proteinopathy. Mol Neurodegener.

[41] Cook CN, Wu Y, Odeh HM, Gendron TF, Jansen-West K, Del Rosso G (2020). C9orf72 poly(GR) aggregation induces TDP-43 proteinopathy. Sci Transl Med.

[42] Saberi S, Stauffer JE, Jiang J, Garcia SD, Taylor AE, Schulte D, et al. Sense-encoded poly-GR dipeptide repeat proteins correlate to neurodegeneration and uniquely co-localize with TDP-43 in dendrites of repeat-expanded C9orf72 amyotrophic lateral sclerosis. Acta Neuropathol 2018;135:459–74. 10.1007/s00401-017-1793-8 PMC5935138 29196813

[43] Weskamp K, Tank EM, Miguez R, McBride JP, Gomez NB, White M (2020). Shortened TDP43 isoforms upregulated by neuronal hyperactivity drive TDP43 pathology in ALS. J Clin Invest.

[44] Arseni D, Hasegawa M, Murzin AG, Kametani F, Arai M, Yoshida M (2022). Structure of pathological TDP-43 filaments from ALS with FTLD. Nature.

[45] Gasset-Rosa F, Lu S, Yu H, Chen C, Melamed Z, Guo L (2019). Cytoplasmic TDP-43 De-mixing independent of stress granules drives inhibition of nuclear import, loss of nuclear TDP-43, and cell death. Neuron.

[46] Schmidt HB, Barreau A, Rohatgi R (2019). Phase separation-deficient TDP43 remains functional in splicing. Nat Commun.

[47] Lippai M, Tirian L, Boros I, Mihaly J, Erdelyi M, Belecz I (2000). The Ketel gene encodes a Drosophila homologue of importin-beta. Genetics.

[48] Goodwin MS, Croft CL, Futch HS, Ryu D, Ceballos-Diaz C, Liu X (2020). Utilizing minimally purified secreted rAAV for rapid and cost-effective manipulation of gene expression in the CNS. Mol Neurodegener.

[49] Kinoshita Y, Ito H, Hirano A, Fujita K, Wate R, Nakamura M (2009). Nuclear contour irregularity and abnormal transporter protein distribution in anterior horn cells in amyotrophic lateral sclerosis. J Neuropathol Exp Neurol.

[50] Nagara Y, Tateishi T, Yamasaki R, Hayashi S, Kawamura M, Kikuchi H (2013). Impaired cytoplasmic-nuclear transport of hypoxia-inducible factor-1alpha in amyotrophic lateral sclerosis. Brain Pathol.

[51] Aizawa H, Yamashita T, Kato H, Kimura T, Kwak S (2019). Impaired Nucleoporins are present in sporadic amyotrophic lateral sclerosis motor neurons that exhibit Mislocalization of the 43-kDa TAR DNA-binding protein. J Clin Neurol.

[52] Yamashita T, Aizawa H, Teramoto S, Akamatsu M, Kwak S (2017). Calpain-dependent disruption of nucleo-cytoplasmic transport in ALS motor neurons. Sci Rep.

[53] Xiao S, MacNair L, McGoldrick P, McKeever PM, McLean JR, Zhang M (2015). Isoform-specific antibodies reveal distinct subcellular localizations of C9orf72 in amyotrophic lateral sclerosis. Ann Neurol.

[54] Anderson EN, Morera AA, Kour S, Cherry JD, Ramesh N, Gleixner A (2021). Traumatic injury compromises nucleocytoplasmic transport and leads to TDP-43 pathology. Elife.

[55] Gleixner AM, Verdone BM, Otte CG, Anderson EN, Ramesh N, Shapiro OR (2022). NUP62 localizes to ALS/FTLD pathological assemblies and contributes to TDP-43 insolubility. Nat Commun.

[56] Brettschneider J, Del Tredici K, Irwin DJ, Grossman M, Robinson JL, Toledo JB (2014). Sequential distribution of pTDP-43 pathology in behavioral variant frontotemporal dementia (bvFTD). Acta Neuropathol.

[57] Brettschneider J, Tredici KD, Toledo JB, Robinson JL, Irwin DJ, Grossman M (2013). Stages of pTDP-43 pathology in amyotrophic lateral sclerosis. Ann Neurol.

[58] Keating SS, San Gil R, Swanson MEV, Scotter EL, Walker AK. TDP-43 pathology: from noxious assembly to therapeutic removal. Prog Neurobiol. 2022;211:102229. 10.1016/j.pneurobio.2022.102229 35101542

[59] Carey JL, Guo L (2022). Liquid-liquid phase separation of TDP-43 and FUS in physiology and pathology of neurodegenerative diseases. Front Mol Biosci.

[60] Springhower CE, Rosen MK, Chook YM (2020). Karyopherins and condensates. Curr Opin Cell Biol.

[61] Fernandez-Nogales M, Lucas JJ (2019). Altered levels and isoforms of tau and nuclear membrane invaginations in Huntington's disease. Front Cell Neurosci.

[62] Eftekharzadeh B, Daigle JG, Kapinos LE, Coyne A, Schiantarelli J, Carlomagno Y (2019). Tau protein disrupts Nucleocytoplasmic transport in Alzheimer's disease. Neuron.

[63] Paonessa F, Evans LD, Solanki R, Larrieu D, Wray S, Hardy J (2019). Microtubules deform the nuclear membrane and disrupt Nucleocytoplasmic transport in tau-mediated Frontotemporal dementia. Cell Rep.

[64] Cornelison GL, Levy SA, Jenson T, Frost B (2019). Tau-induced nuclear envelope invagination causes a toxic accumulation of mRNA in Drosophila. Aging Cell.

[65] Eftekharzadeh B, Daigle JG, Kapinos LE, Coyne A, Schiantarelli J, Carlomagno Y (2018). Tau protein disrupts Nucleocytoplasmic transport in Alzheimer's disease. Neuron.

[66] Gasset-Rosa F, Chillon-Marinas C, Goginashvili A, Atwal RS, Artates JW, Tabet R (2017). Polyglutamine-expanded Huntingtin exacerbates age-related disruption of nuclear integrity and Nucleocytoplasmic transport. Neuron.

[67] Shani V, Safory H, Szargel R, Wang N, Cohen T, Elghani FA (2019). Physiological and pathological roles of LRRK2 in the nuclear envelope integrity. Hum Mol Genet.

[68] Darling AL, Shorter J (2021). Combating deleterious phase transitions in neurodegenerative disease. Biochim Biophys Acta, Mol Cell Res.

[69] Guo L, Fare CM, Shorter J (2019). Therapeutic dissolution of aberrant phases by nuclear-import receptors. Trends Cell Biol.

[70] Hutten S, Usluer S, Bourgeois B, Simonetti F, Odeh HM, Fare CM (2020). Nuclear import receptors directly bind to arginine-rich dipeptide repeat proteins and suppress their pathological interactions. Cell Rep.

[71] Doll SG, Cingolani G. Importin alpha/beta and the tug of war to keep TDP-43 in solution: quo vadis? Bioessays. 2022;44:e2200181. 10.1002/bies.202200181 PMC9969346 36253101

[72] Doll SG, Meshkin H, Bryer AJ, Li F, Ko YH, Lokareddy RK (2022). Recognition of the TDP-43 nuclear localization signal by importin alpha1/beta. Cell Rep.

[73] Gonzalez A, Mannen T, Cagatay T, Fujiwara A, Matsumura H, Niesman AB (2021). Mechanism of karyopherin-beta2 binding and nuclear import of ALS variants FUS(P525L) and FUS(R495X). Sci Rep.

[74] Baade I, Hutten S, Sternburg EL, Porschke M, Hofweber M, Dormann D (2021). The RNA-binding protein FUS is chaperoned and imported into the nucleus by a network of import receptors. J Biol Chem.

[75] Lin YC, Kumar MS, Ramesh N, Anderson EN, Nguyen AT, Kim B (2021). Interactions between ALS-linked FUS and nucleoporins are associated with defects in the nucleocytoplasmic transport pathway. Nat Neurosci.

[76] Schmidt HB, Gorlich D (2016). Transport selectivity of nuclear pores, phase separation, and Membraneless organelles. Trends Biochem Sci.

[77] Jakel S, Mingot JM, Schwarzmaier P, Hartmann E, Gorlich D (2002). Importins fulfil a dual function as nuclear import receptors and cytoplasmic chaperones for exposed basic domains. EMBO J.

[78] Padavannil A, Sarkar P, Kim SJ, Cagatay T, Jiou J, Brautigam CA (2019). Importin-9 wraps around the H2A-H2B core to act as nuclear importer and histone chaperone. Elife.

[79] Lang A, Eriksson J, Schink KO, Lang E, Blicher P, Polec A (2017). Visualization of PML nuclear import complexes reveals FG-repeat nucleoporins at cargo retrieval sites. Nucleus.

[80] Milles S, Huy Bui K, Koehler C, Eltsov M, Beck M, Lemke EA (2013). Facilitated aggregation of FG nucleoporins under molecular crowding conditions. EMBO Rep.

[81] Winton MJ, Van Deerlin VM, Kwong LK, Yuan W, Wood EM, Yu CE (2008). A90V TDP-43 variant results in the aberrant localization of TDP-43 in vitro. FEBS Lett.

[82] Wobst HJ, Wesolowski SS, Chadchankar J, Delsing L, Jacobsen S, Mukherjee J (2017). Cytoplasmic Relocalization of TAR DNA-binding protein 43 is not sufficient to reproduce cellular pathologies associated with ALS in vitro. Front Mol Neurosci.

[83] Hayes LR, Duan L, Bowen K, Kalab P, Rothstein JD (2020). C9orf72 arginine-rich dipeptide repeat proteins disrupt karyopherin-mediated nuclear import. Elife.

[84] Krach F, Batra R, Wheeler EC, Vu AQ, Wang R, Hutt K (2018). Transcriptome-pathology correlation identifies interplay between TDP-43 and the expression of its kinase CK1E in sporadic ALS. Acta Neuropathol.

[85] Odeh HM, Fare CM, Shorter J (2022). Nuclear-import receptors counter deleterious phase transitions in neurodegenerative disease. J Mol Biol.

