# Supplementary material for: Nuclear import receptors are recruited by FG-nucleoporins to rescue hallmarks of TDP-43 proteinopathy
Source: Mol Neurodegener. 2022 Dec 8;17:80. doi: 10.1186/s13024-022-00585-1 (PMC9733332; doi:10.1186/s13024-022-00585-1)
Supplement: Supplementary file 1 — Additional file 1: Supplementary Fig. 1. KPNB1 specifically reduces insoluble protein levels of TDP-CTF. a, (Top) Immunofluorescence (IF) of SH-SY5Y cells co-expressing mCherry-TDP-CTF with GFP or GFP-KPNB1. mCherry-TDP-CTF forms cytoplasmic aggregates but shows diffuse localization in the presence of GFP-KPNB1. Hoechst staining was used to outline nuclei. Scale bar: 5 μm. (Bottom) Western blot analysis and quantification of insoluble mCherry-TDP-CTF and endogenous TDP-43 protein levels in SH-SY5Y cells expressing GFP or GFP-KPNB1. KPNB1 significantly reduced insoluble TDP-CTF levels, while endogenous insoluble TDP-43 levels were unaffected. β-tubulin was used as a loading control. Statistical analysis was performed using two-sided Student’s t-test (***p < 0.001, n = 10). b, Western blot analysis of soluble and insoluble GFP-TDP-CTF co-expressed with mCherry, mCherry-KPNB1 or untagged KPNB1 in HEK293T cells. KPNB1 only reduces insoluble TDP-CTF levels. β-tubulin was used as a loading control. c, IF of HEK293T cells co-expressing GFP-TDP-CTF or GFP-TDP-43mNLS with mCherry or mCherry-KPNB1. TDP-CTF and TDP-43mNLS form cytoplasmic aggregates positive for phospho-TDP-43S409/410 but show diffuse localization with no pathological hyperphosphorylation in the presence of mCherry-KPNB1. Arrowheads point to colocalization. Scale bar: 5 μm. d, Western blot analysis and quantification of insoluble GFP-tagged TDP-CTFWT, TDP-CTFQ331K, TDP-CTFM337V and TDP-CTFA382T in HEK293T cells expressing mCherry or mCherry-KPNB1. KPNB1 similarly reduced insoluble wild-type and ALS-derived mutant TDP-CTF protein levels. β-tubulin was used as a loading control. Statistical analysis was performed using two-sided Student’s t-test (***p < 0.001, n = 3). e, Western blot analysis of total protein levels of GFP-tagged TDP-CTF or TDP-43 in HEK293T cells expressing mCherry, mCherry-KPNB1 or untagged KPNB1. Cells were lysed in 7 M urea buffer to collect total protein. KPNB1 reduces total protein levels [file 13024_2022_585_MOESM1_ESM.docx]

**Supplementary figures**


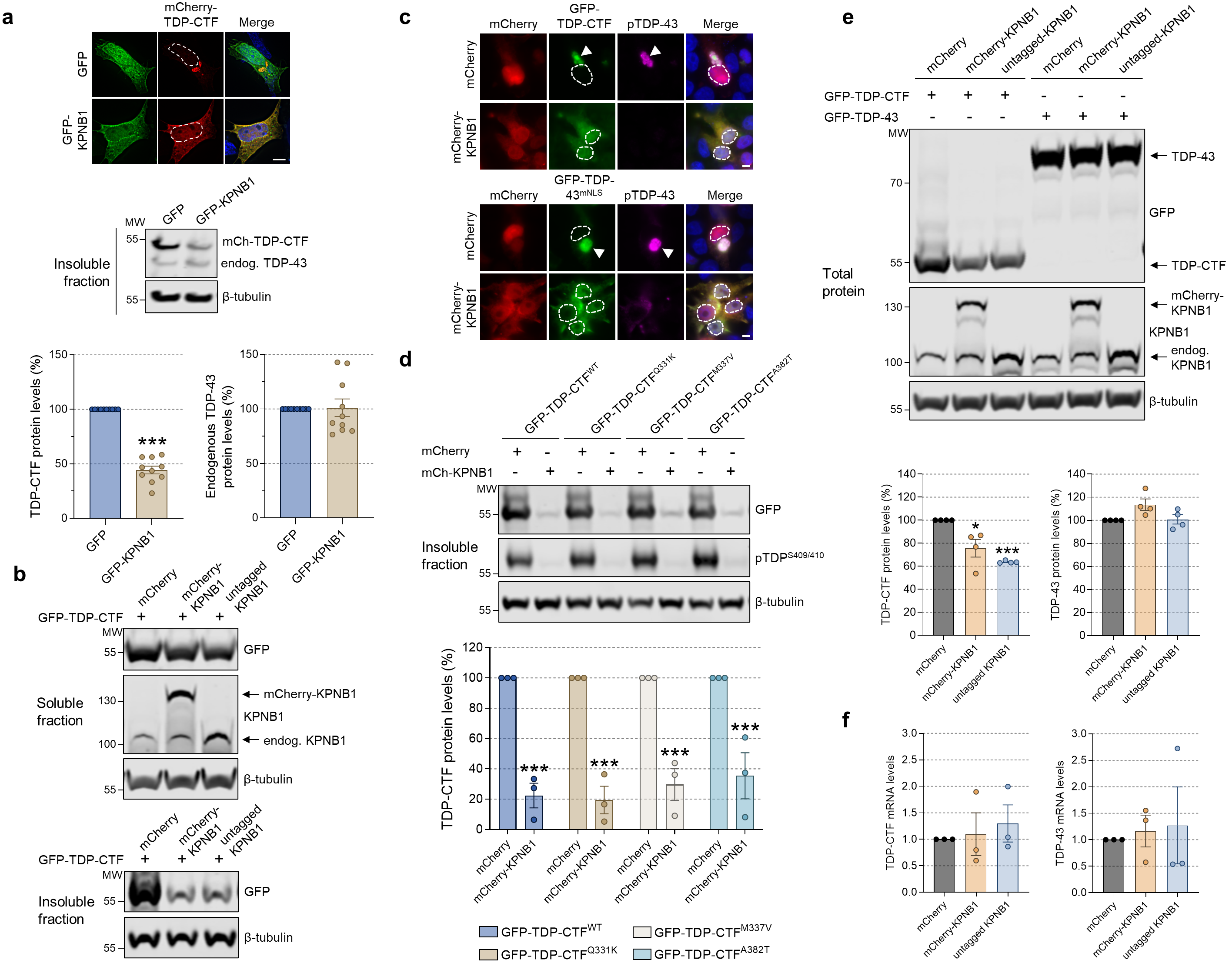


**Supplementary Fig. 1** KPNB1 specifically reduces insoluble protein levels of TDP-CTF. **a**, (Top) Immunofluorescence (IF) of SH-SY5Y cells co-expressing mCherry-TDP-CTF with GFP or GFP-KPNB1. mCherry-TDP-CTF forms cytoplasmic aggregates but shows diffuse localization in the presence of GFP-KPNB1. Hoechst staining was used to outline nuclei. Scale bar: 5 μm. (Bottom) Western blot analysis and quantification of insoluble mCherry-TDP-CTF and endogenous TDP-43 protein levels in SH-SY5Y cells expressing GFP or GFP-KPNB1. KPNB1 significantly reduced insoluble TDP-CTF levels, while endogenous insoluble TDP-43 levels were unaffected. β-tubulin was used as a loading control. Statistical analysis was performed using two-sided Student’s *t*-test (***p<0.001, n=10). **b**, Western blot analysis of soluble and insoluble GFP-TDP-CTF co-expressed with mCherry, mCherry-KPNB1 or untagged KPNB1 in HEK293T cells. KPNB1 only reduces insoluble TDP-CTF levels. β-tubulin was used as a loading control. **c,** IF of HEK293T cells co-expressing GFP-TDP-CTF or GFP-TDP-43^mNLS^ with mCherry or mCherry-KPNB1. TDP-CTF and TDP-43^mNLS^ form cytoplasmic aggregates positive for phospho-TDP-43^S409/410^ but show diffuse localization with no pathological hyperphosphorylation in the presence of mCherry-KPNB1. Arrowheads point to colocalization. Scale bar: 5 μm. **d,** Western blot analysis and quantification of insoluble GFP-tagged TDP-CTF^WT^, TDP-CTF^Q331K^, TDP-CTF^M337V^ and TDP-CTF^A382T^ in HEK293T cells expressing mCherry or mCherry-KPNB1. KPNB1 similarly reduced insoluble wild-type and ALS-derived mutant TDP-CTF protein levels. β-tubulin was used as a loading control. Statistical analysis was performed using two-sided Student’s *t*-test (***p<0.001, n=3). **e,** Western blot analysis of total protein levels of GFP-tagged TDP-CTF or TDP-43 in HEK293T cells expressing mCherry, mCherry-KPNB1 or untagged KPNB1. Cells were lysed in 7 M urea buffer to collect total protein. KPNB1 reduces total protein levels of TDP-CTF, but not full-length TDP-43. β-tubulin was used as a loading control. Statistical analysis was performed using one-way ANOVA and Bonferroni’s post hoc test (*p<0.05, ***p<0.001, n=4). **f,** *TDP-CTF* and *TDP-43* transcript levels were quantified in HEK293T cells co-expressing GFP-tagged TDP-CTF or TDP-43 with mCherry, mCherry-KPNB1, or untagged KPNB1. *GAPDH* was used as a reference to normalize the transcript levels. Statistical analysis was performed using one-way ANOVA and Bonferroni’s post hoc test (n=3).


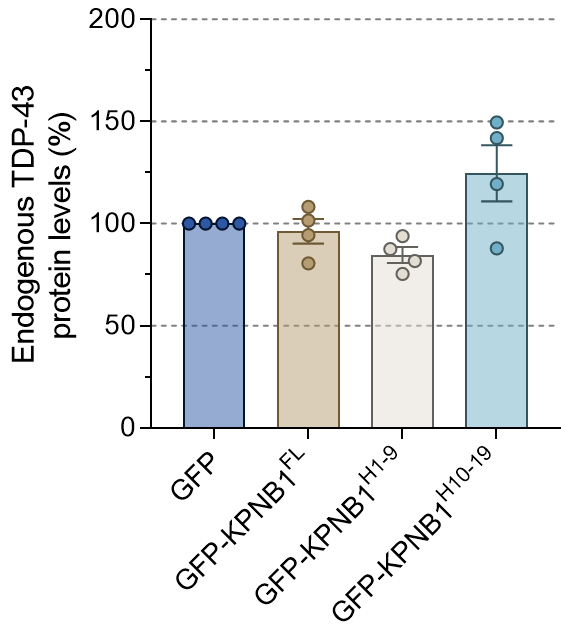


**Supplementary Fig. 2** Expression of full-length KPNB1 and its N-terminal half do not reduce endogenous insoluble TDP-43 levels. Quantification of endogenous insoluble TDP-43 protein levels in SH-SY5Y cells co-expressing mCherry-TDP-CTF with GFP, GFP-KPNB1 full-length (FL), H1-9 or H10-19. β-tubulin was used as a loading control. Statistical analysis was performed using one-way ANOVA and Bonferroni’s post hoc test (n=4).


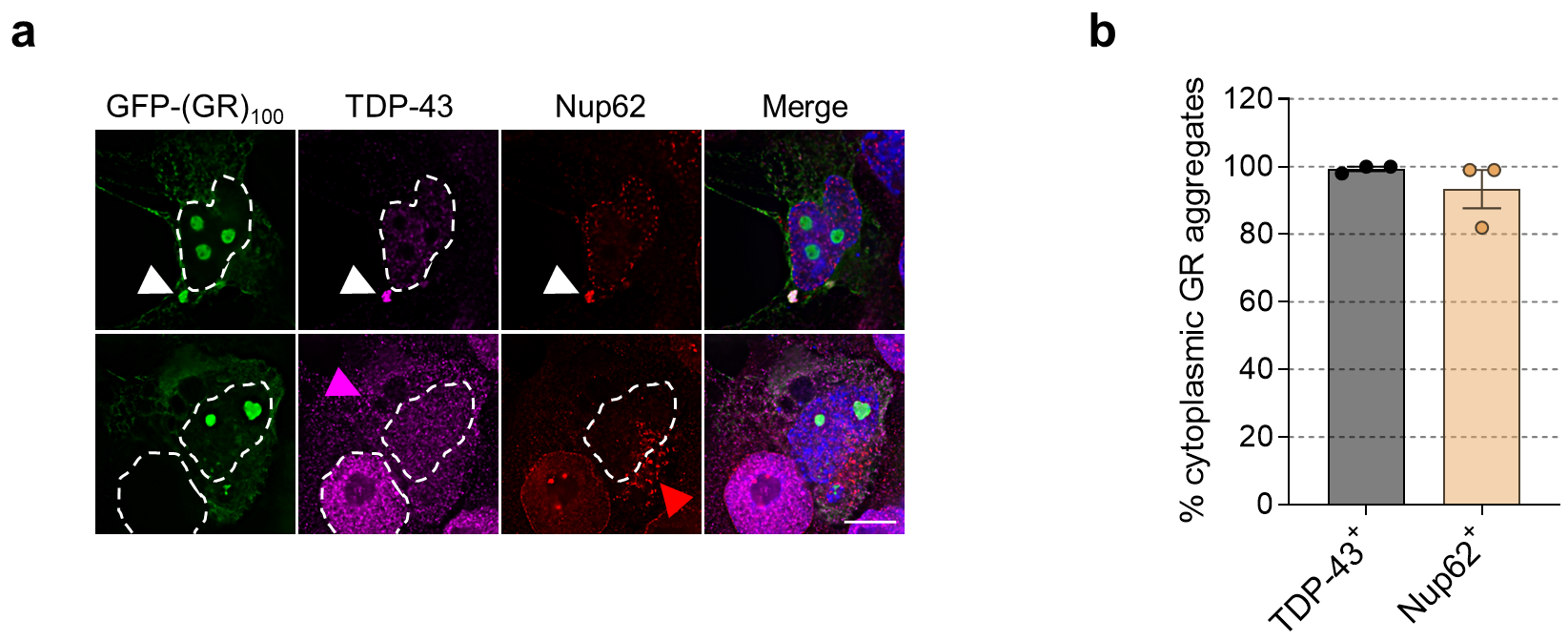


**Supplementary Fig. 3** Cytoplasmic GFP-(GR)_100_ aggregates sequester endogenous TDP-43 and Nup62. **a,** Immunofluorescence of HEK293T cells expressing GFP-(GR)_100_ and stained for endogenous TDP-43 and Nup62. Only cytoplasmic GFP-(GR)_100_ aggregates are immunopositive for TDP-43 and Nup62. White arrowheads point to cytoplasmic poly(GR) aggregates. Nuclear GFP-(GR)_100_ aggregates render TDP-43 staining diffuse (purple arrowhead), while Nup62 accumulates as puncta in the cytoplasm (red arrowhead). Hoechst staining was used to outline nuclei. Scale bar: 5 μm. **b,** Quantification of the percentage of cytoplasmic GFP-(GR)_100_ aggregates positive for TDP-43 or Nup62 in HEK293T cells. Almost all cells are positive for both proteins. Statistical analysis was performed using two-sided Student’s *t*-test (three independent experiments; n=239-249 cells per group).


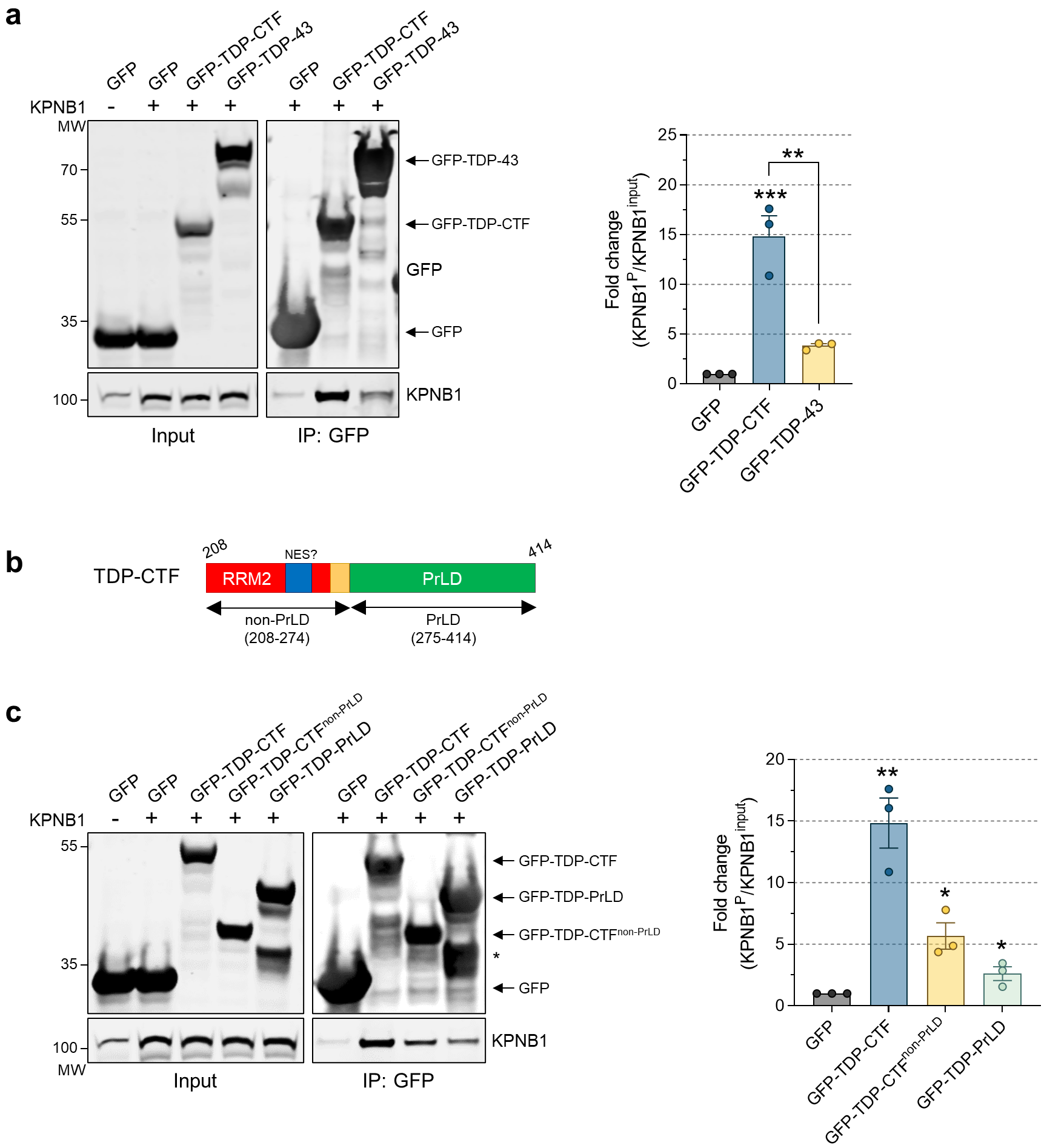


**Supplementary Fig. 4** Untagged KPNB1 interacts with both the RRM2 and PrLD of TDP-CTF. **a**, (Left) Lysates from HEK293T cells expressing GFP alone, or untagged KPNB1 with GFP, GFP-TDP-CTF or GFP-TDP-43 were subjected to immunoprecipitation with GFP-Trap magnetic beads. Whole cell lysates (input) and immunoprecipitates (IP) were subjected to western blot analysis using indicated antibodies. (Right) Quantification of relative KPNB1 levels in IP normalized by KPNB1 levels in input. Statistical analysis was performed using one-way ANOVA and Bonferroni’s post hoc test (**p<0.01, ***p<0.001, n=3). **b**, Schematic domain structure of TDP-CTF full-length (208-414). TDP-CTF contains part of the RRM2 (non-PrLD, 208-274) and the C-terminal PrLD (275-414). **c,** (Left) Lysates from HEK293T cells expressing GFP alone, or untagged KPNB1 with GFP, GFP-TDP-CTF, GFP-TDP-CTF^non-PrLD^ or GFP-TDP-PrLD were subjected to immunoprecipitation with GFP-Trap magnetic beads. Whole cell lysates (input) and immunoprecipitates (IP) were subjected to western blot analysis using indicated antibodies. (Right) Quantification of relative KPNB1 levels in IP normalized by KPNB1 levels in input. Statistical analysis was performed using one-way ANOVA and Bonferroni’s post hoc test (*p<0.05, **p<0.01, n=3). *cleavage products.

**
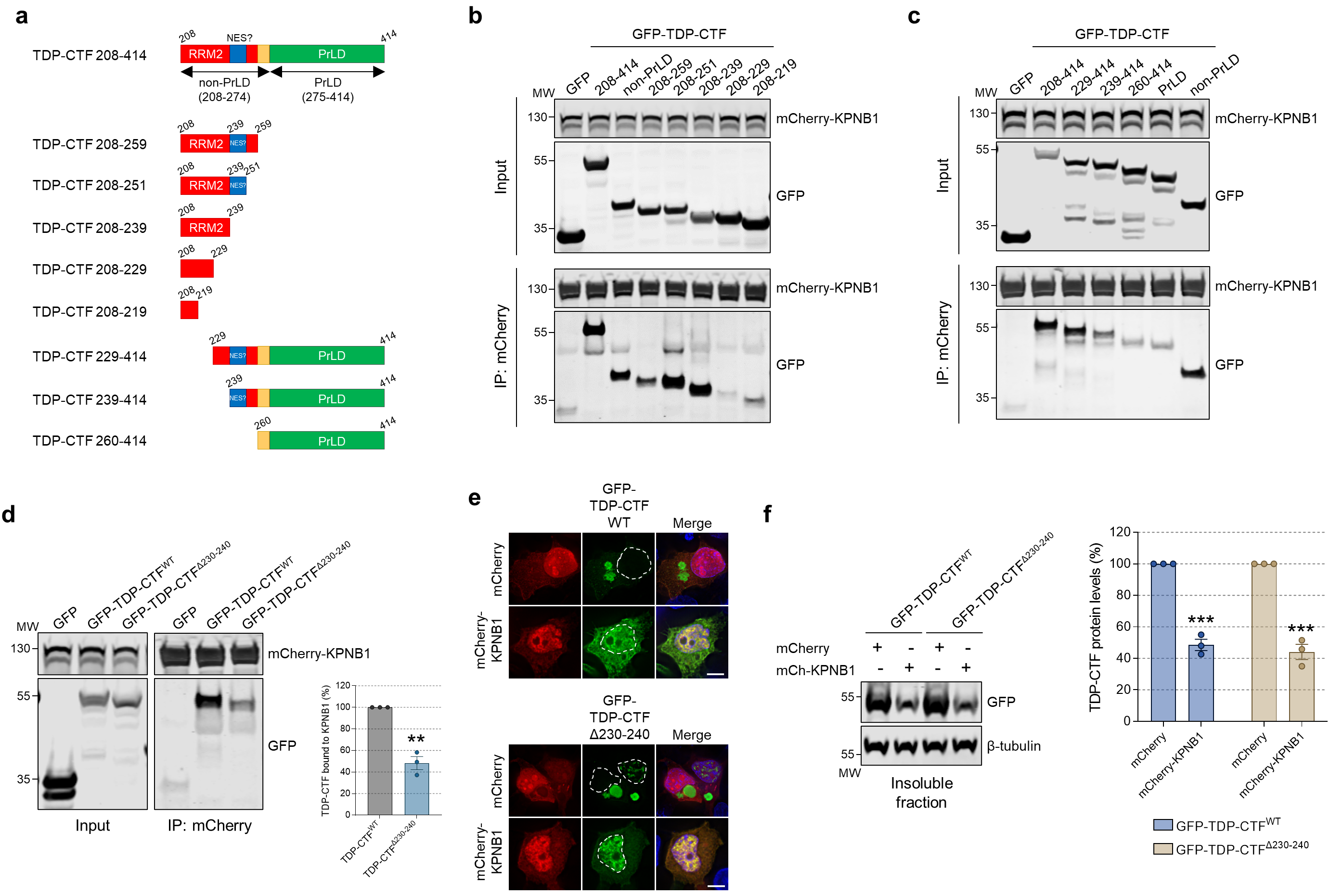
**

**Supplementary Fig. 5** KPNB1 reduces TDP-CTF aggregation independent of its interaction with the RRM2 region. **a**, Schematic domain structures of full-length TDP-CTF (208-414), and its C-terminal and N-terminal deletion constructs used for co-IP experiments. **b**, Lysates from HEK293T cells co-expressing mCherry-KPNB1 with GFP, GFP-TDP-CTF or GFP-TDP-CTF C-terminal deletion constructs were subjected to immunoprecipitation with RFP-Trap magnetic beads. Whole cell lysates (input) and immunoprecipitates (IP) were subjected to western blot analysis using indicated antibodies. Deletion of amino-acids 230-240 in TDP-CTF reduced its binding to KPNB1. **c**, Lysates from HEK293T cells co-expressing mCherry-KPNB1 with GFP, GFP-TDP-CTF or GFP-TDP-CTF N-terminal deletion constructs were subjected to immunoprecipitation with RFP-Trap magnetic beads. Whole cell lysates (input) and immunoprecipitates (IP) were subjected to western blot analysis using indicated antibodies. Deletion of amino-acids 230-240 in TDP-CTF reduces its binding to KPNB1. TDP-PrLD weakly interacts with KPNB1. **d**, (Left) Lysates from HEK293T cells co-expressing mCherry-KPNB1 with GFP, GFP-TDP-CTF^WT^ or GFP-TDP-CTF^Δ230-240^ were subjected to immunoprecipitation with RFP-Trap magnetic beads. Whole cell lysates (input) and immunoprecipitates (IP) were subjected to western blot analysis using indicated antibodies. KPNB1 interacts less with TDP-CTF^Δ230-240^. (Right) Quantification of relative GFP levels in IP normalized by GFP levels in input confirm that GFP-TDP-CTF^Δ230-240^ interacts less with mCherry-KPNB1. Statistical analysis was performed using two-sided Student’s *t*-test (**p<0.01, n=3). **e**, Immunofluorescence of HEK293T cells co-expressing GFP-tagged TDP-CTF^WT^ or TDP-CTF^Δ230-240^ with mCherry or mCherry-KPNB1. TDP-CTF^Δ230-240^ forms small nuclear and big cytoplasmic aggregates. KPNB1 reduces both TDP-CTF^WT^ and TDP-CTF^Δ230-240^ aggregates and makes TDP-CTF^Δ230-240^ more nuclear. Hoechst staining was used to outline nuclei. Scale bar: 5 μm. **f**, Western blot analysis and quantification of insoluble GFP-tagged TDP-CTF^WT^ or TDP-CTF^Δ230-240^ in HEK293T cells expressing mCherry or mCherry-KPNB1. KPNB1 equally reduced insoluble TDP-CTF^WT^ and TDP-CTF^Δ230-240^ protein levels. β-tubulin was used as a loading control. Statistical analysis was performed using two-way ANOVA and Bonferroni’s post hoc test (***p<0.001, n=3).


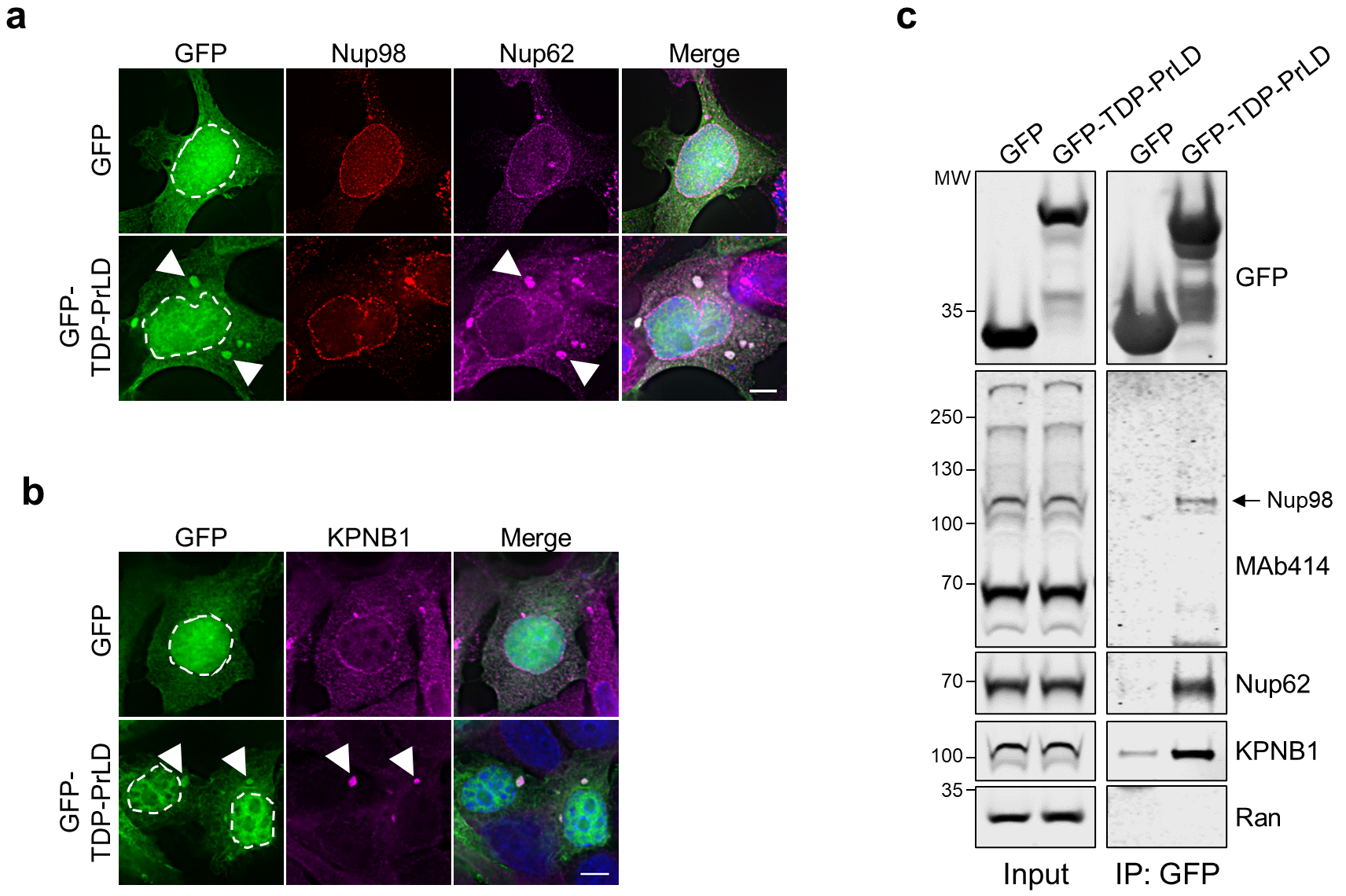


**Supplementary Fig. 6** The PrLD of TDP-43 interacts with FG-Nups and KPNB1. **a,** Immunofluorescence (IF) of HEK293T cells expressing GFP or GFP-TDP-PrLD and stained for endogenous Nup98 and Nup62. TDP-PrLD forms small cytoplasmic foci positive for Nup62. Hoechst staining was used to outline nuclei. Scale bar: 5 μm. **b,** IF of HEK293T cells expressing GFP or GFP-TDP-PrLD and stained for endogenous KPNB1. KPNB1 colocalizes with TDP-PrLD foci in the cytoplasm. Hoechst staining was used to outline nuclei. Scale bar: 5 μm. **c,** Lysates from HEK293T cells expressing GFP or GFP-TDP-PrLD were subjected to immunoprecipitation with GFP-Trap magnetic beads. Whole cell lysates (input) and immunoprecipitates (IP) were subjected to western blot analysis using indicated antibodies. The PrLD of TDP-43 interacts with Nup62, Nup98 and KPNB1, but not with Ran.

**
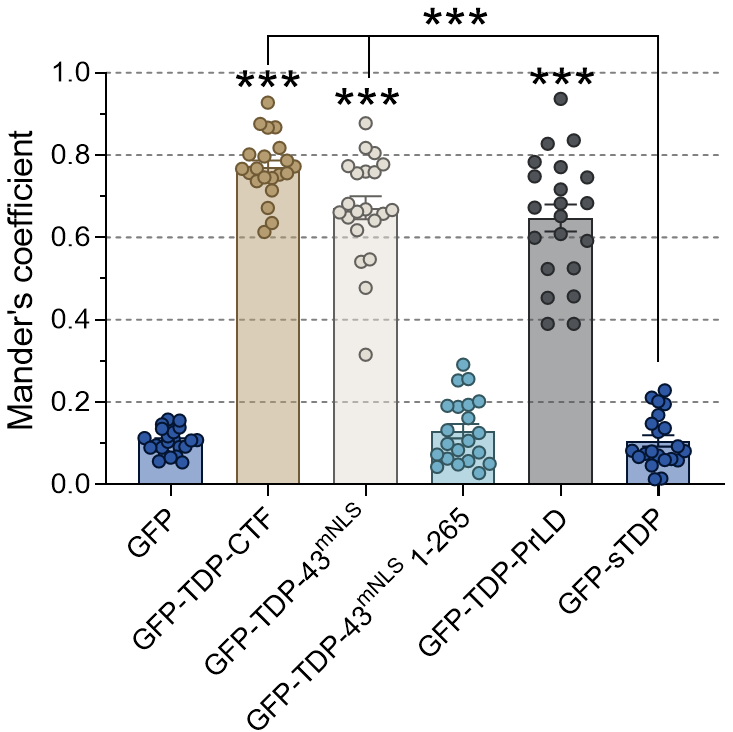
**

**Supplementary Fig. 7** Colocalization of Nup62 with TDP-43 constructs depends on the PrLD. Colocalization between Nup62-mCherry and GFP or GFP-tagged TDP-43 constructs was measured using the Mander’s overlap coefficient. Values close to 0 and 1 indicate a weak and strong colocalization, respectively. Statistical analysis was performed using one-way ANOVA and Bonferroni’s post hoc test (***p<0.001, n=21-23 cells per group).


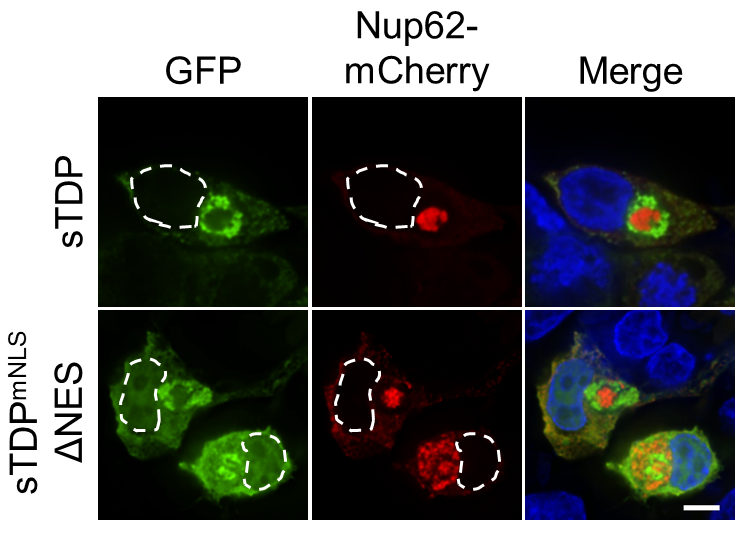


**Supplementary Fig. 8** The NES of sTDP does not prevent it from binding to Nup62 aggregates. Immunofluorescence of HEK293T cells co-expressing Nup62-mCherry with either sTDP or sTDP^mNLS^ ΔNES. Nup62 aggregates do not colocalize with sTDP even after mutating its NLS and NES. Hoechst staining was used to outline nuclei. Scale bar: 5 μm.

**
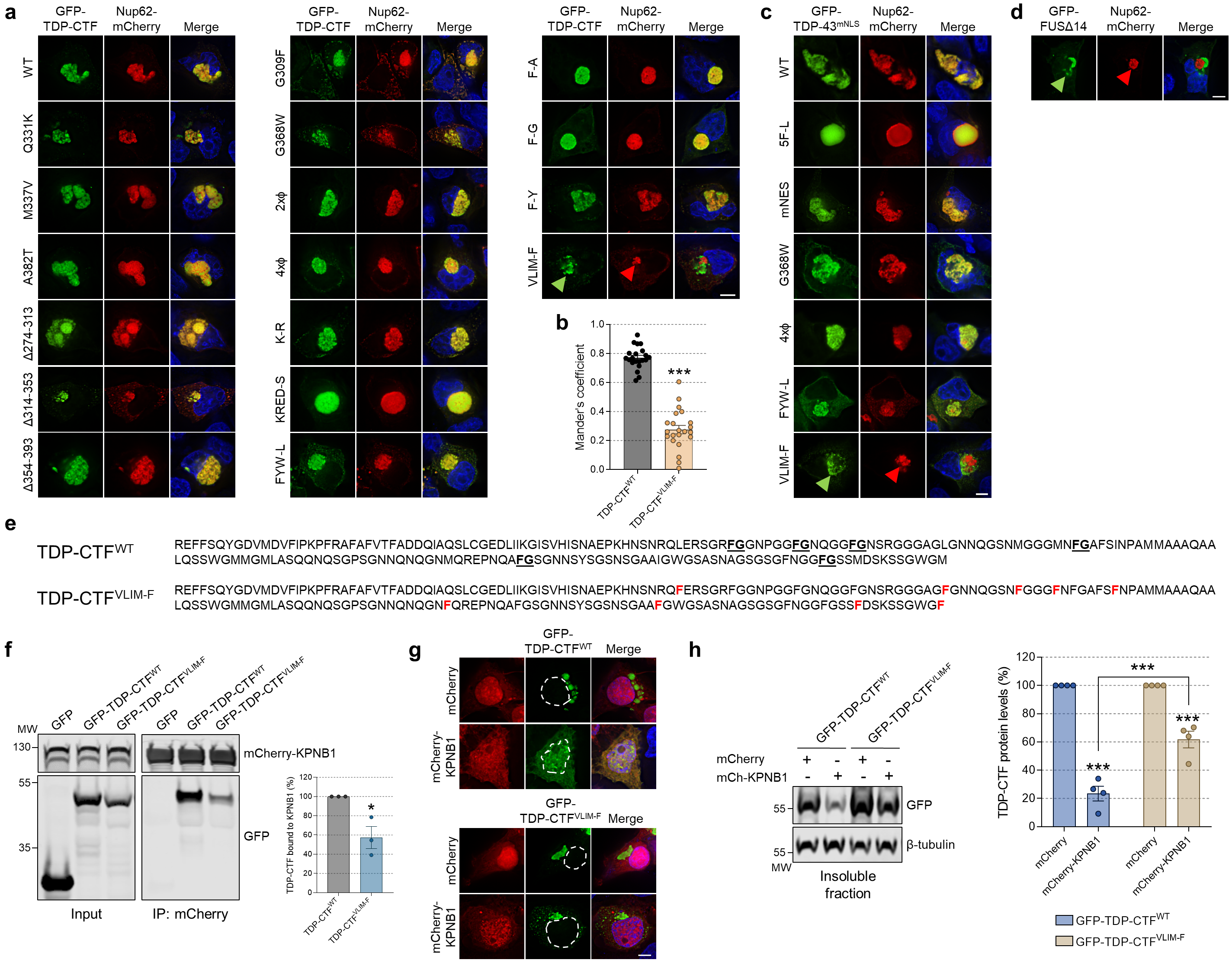
**

**Supplementary Fig. 9** TDP-CTF PrLD mutations that reduce its association with Nup62 abrogate its interaction and reduced aggregation by KPNB1. **a,** Immunofluorescence (IF) of HEK293T cells co-expressing Nup62-mCherry with either GFP-tagged TDP-CTF^WT^ or TDP-CTF constructs harboring different mutations in the PrLD. All TDP-CTF mutant variants colocalize with Nup62 aggregates, except for TDP-CTF^VLIM-F^ (green arrowhead) which accumulates in close proximity to Nup62 (red arrowhead). Scale bar: 5 μm. **b**, Colocalization between Nup62-mCherry and GFP-tagged TDP-CTF^WT^ or TDP-CTF^VLIM-F^ was measured using the Mander’s overlap coefficient. Values close to 0 and 1 indicate a weak and strong colocalization, respectively. Statistical analysis was performed using two-sided Student’s *t*-test (***p<0.001, n=21-22 cells per group). **c,** IF of HEK293T cells co-expressing Nup62-mCherry with either GFP-tagged wild-type or mutant TDP-43^mNLS^ constructs. RNA-binding deficient TDP-43^mNLS^ (TDP-43^mNLS^ 5F-L) can still colocalize with Nup62. Only TDP-43^mNLS^ VLIM-F aggregates (green arrowhead) do not contain Nup62 (red arrowhead). Scale bar: 5 μm. **d,** IF of HEK293T cells co-expressing Nup62-mCherry with GFP-FUSΔ14. Cytoplasmic FUS aggregates (green arrowhead) do not colocalize with Nup62 (red arrowhead). Scale bar: 5 μm. **e,** Protein sequences of TDP-CTF^WT^ and TDP-CTF^VLIM-F^ showing FG repeats (underlined) and VLIM-F mutations (in red) in the PrLD. **f,** (Left) Lysates from HEK293T cells co-expressing mCherry-KPNB1 with GFP, GFP-tagged TDP-CTF^WT^ or TDP-CTF^VLIM-F^ were subjected to immunoprecipitation with RFP-Trap magnetic beads. Whole cell lysates (input) and immunoprecipitates (IP) were subjected to western blot analysis using indicated antibodies. Introducing VLIM-F mutations in TDP-CTF significantly reduces its binding to KPNB1. (Right) Relative GFP levels in IP normalized by GFP levels in input confirm that GFP-TDP-CTF^VLIM-F^ interacts less with mCherry-KPNB1. Statistical analysis was performed using two-sided Student’s *t*-test (*p<0.05, n=3). **g,** IF of HEK293T cells co-expressing GFP-tagged TDP-CTF^WT^ or TDP-CTF^VLIM-F^ with mCherry or mCherry-KPNB1. KPNB1 only reduces the size of TDP-CTF^VLIM-F^ aggregates. Hoechst staining was used to outline nuclei. Scale bar: 5 μm. **h**, Western blot analysis and quantification of insoluble GFP-tagged TDP-CTF^WT^ or TDP-CTF^VLIM-F^ in HEK293T cells expressing mCherry or mCherry-KPNB1. KPNB1 strongly decreased protein levels of insoluble TDP-CTF^WT^ but not TDP-CTF^VLIM-F^. β-tubulin was used as a loading control. Statistical analysis was performed using two-way ANOVA and Bonferroni’s post hoc test (***p<0.001, n=4).


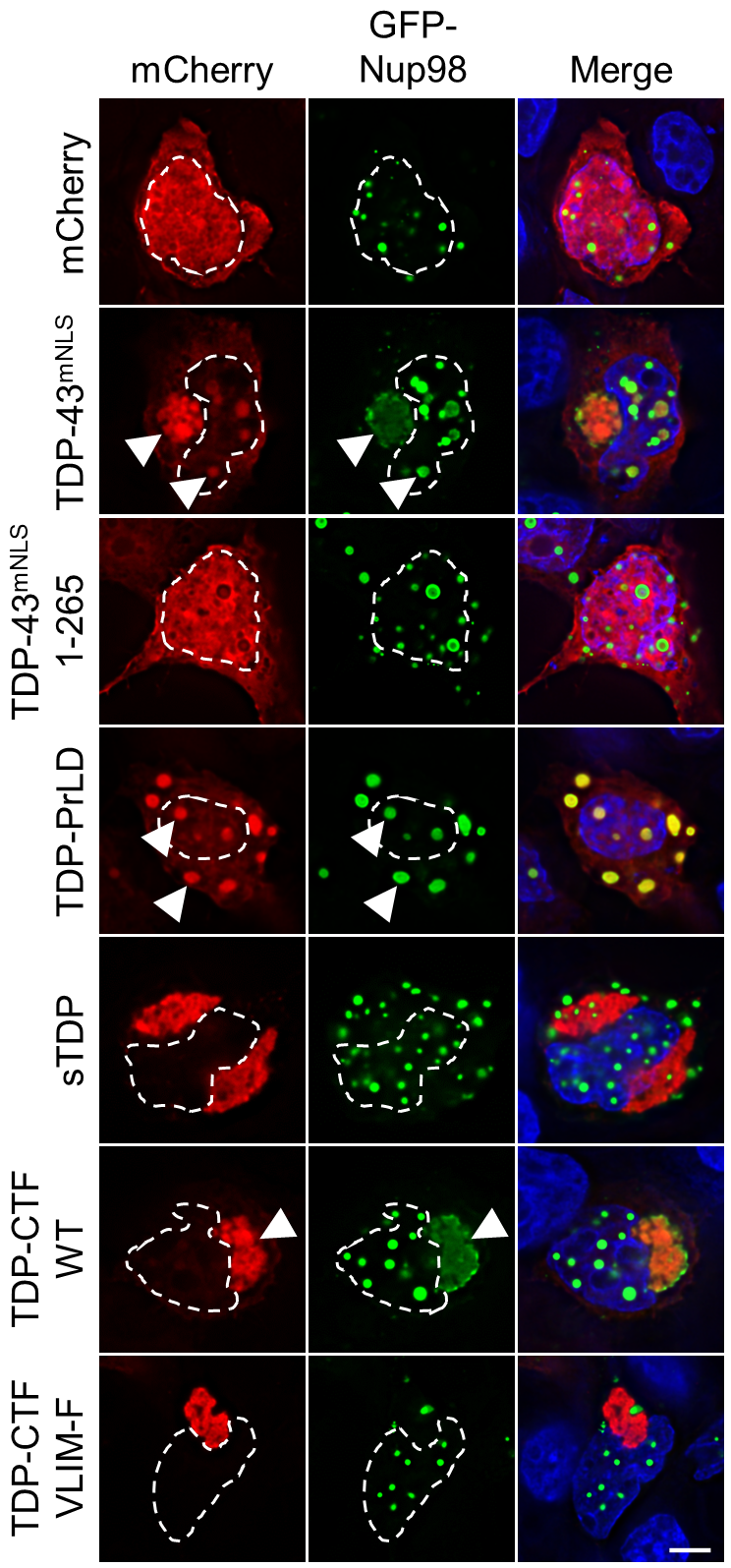


**Supplementary Fig. 10** Nup98 strongly associates with TDP-CTF^WT^, but not TDP-CTF^VLIM-F^ and sTDP. Immunofluorescence of HEK293T cells co-expressing GFP-Nup98 with mCherry or mCherry-tagged TDP-43^mNLS^, TDP-43^mNLS^ 1-265, TDP-PrLD, sTDP, TDP-CTF^WT^ or TDP-CTF^VLIM-F^. Arrowheads point to co-aggregation. Hoechst staining was used to outline nuclei. Scale bar: 5 μm.


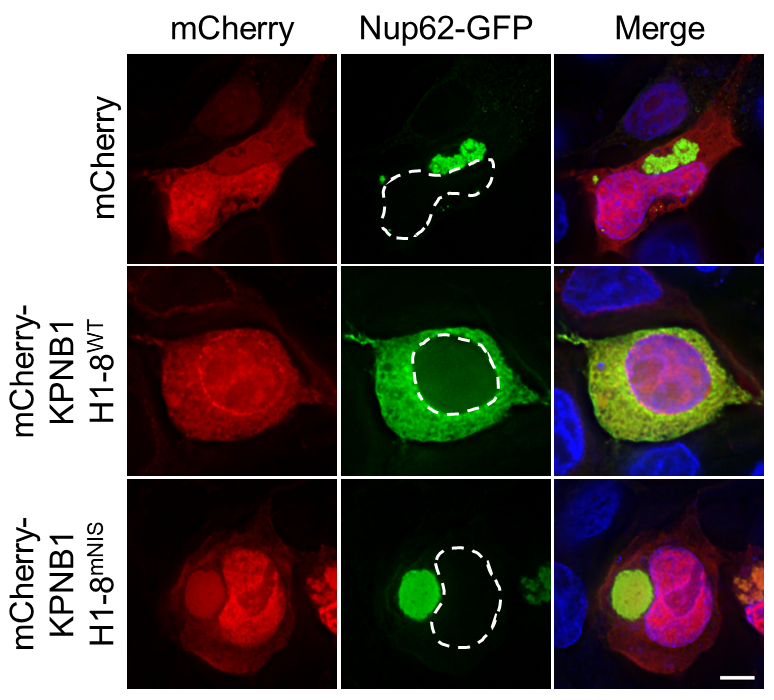


**Supplementary Fig. 11** KPNB1 H1-8 reduces cytoplasmic Nup62 aggregates. Immunofluorescence of HEK293T cells co-expressing Nup62-GFP with mCherry, mCherry-tagged KPNB1 H1-8^WT^ or H1-8^mNIS^. KPNB1 H1-8^WT^ suppresses Nup62 aggregates, whereas NIS mutations abolished this activity. Hoechst staining was used to outline nuclei. Scale bar: 5 μm.


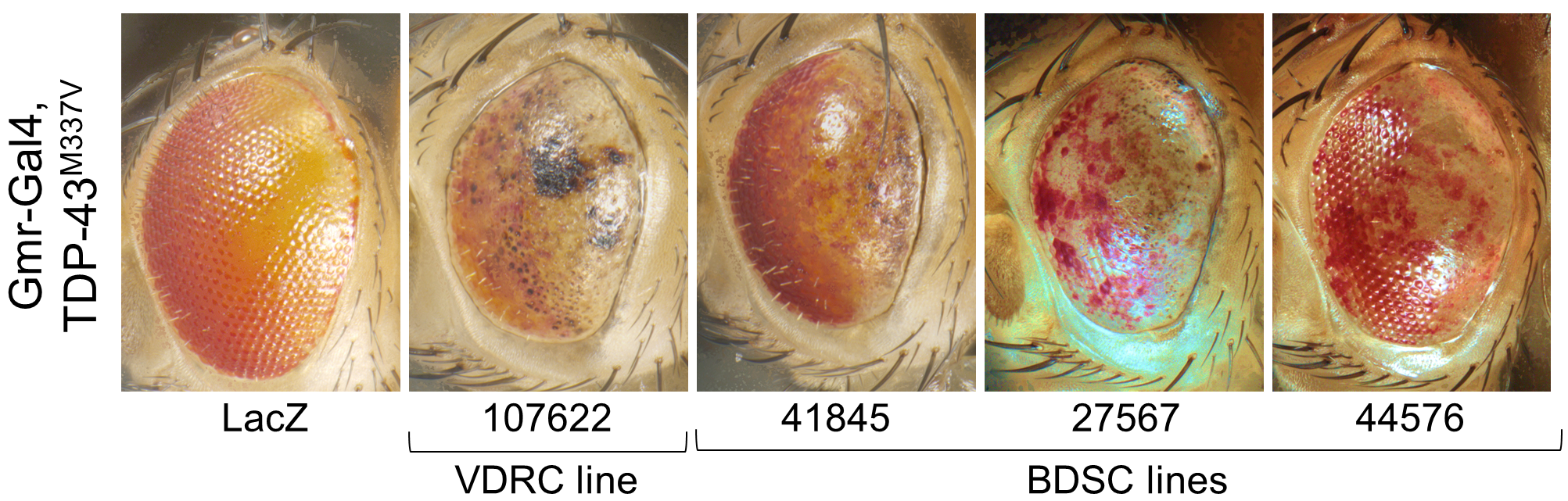


**Supplementary Fig. 12** Multiple Ketel RNAi lines enhance hTDP-43^M337V^ toxicity in the fly eye when expressed under the control of the Gmr-Gal4 driver.


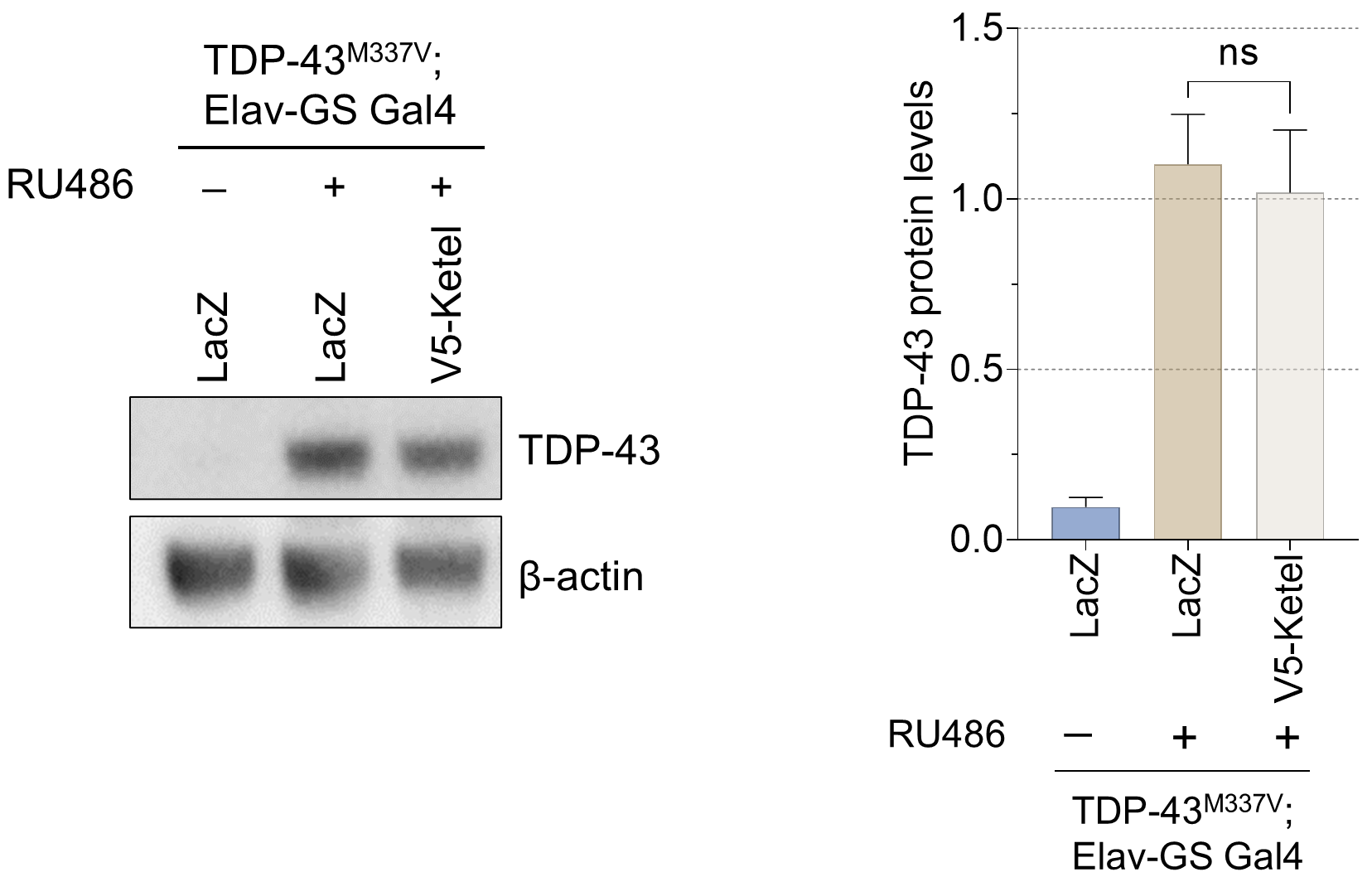


**Supplementary Fig. 13** Ketel overexpression does not change total levels of human TDP-43 protein in flies. Western blot analysis of hTDP-43 expression in fly brains. hTDP-43 is expressed upon administration of RU486, and Ketel overexpression had no effect on total protein levels of TDP-43. β-actin was used as a loading control. Statistical analysis was performed using one-way ANOVA and Sidak’s post hoc test (n=3).


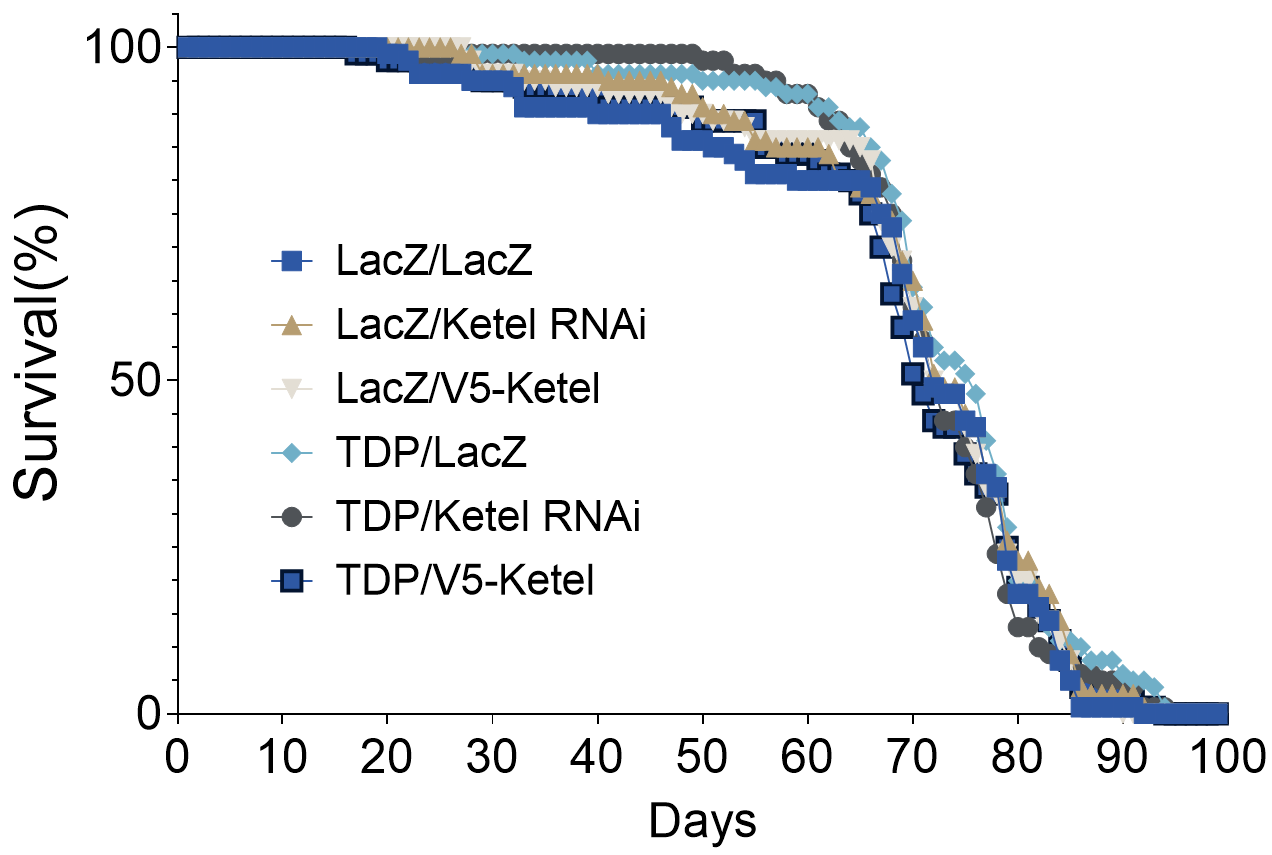


**Supplementary Fig. 14** Survival *Kaplan-Meier* curves of Elav-GS flies carrying the indicated transgenes in the absence of RU486. Note that all the negative controls yield similar results.

**
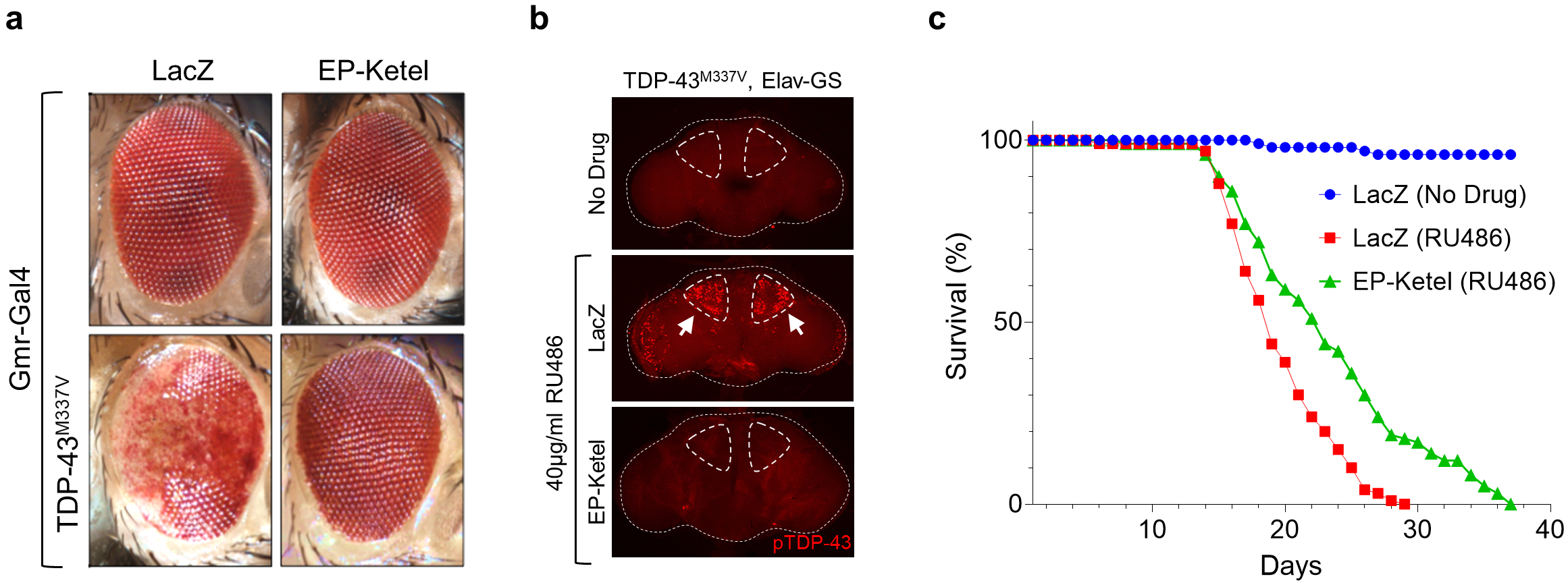
**

**Supplementary Fig. 15** The EP-Ketel insertion P{EPgy2}Fs(2)Ket^EY06666^ rescues mutant hTDP-43 toxicity in flies. **a,** EP-Ketel suppresses TDP-43^M337V^ toxicity in the *Drosophila* eye. **b,** EP-Ketel reduces phospho-TDP-43 staining in the adult brain when co-expressed using the Elav-GS driver and exposed to RU486. **c,** RU486-treated flies carrying the Elav-GS driver, mutant TDP-43 and EP-Ketel (green line) live longer compared to flies carrying the control LacZ transgene (red line). The blue line corresponds to LacZ; Elav-GS Gal4 flies without drug (solvent only) as a negative control. Survival analysis was performed using the OASIS online tool, and p-values were calculated using Fisher’s exact test (n=100, ****p<0.0001 for all the genotypes).

**
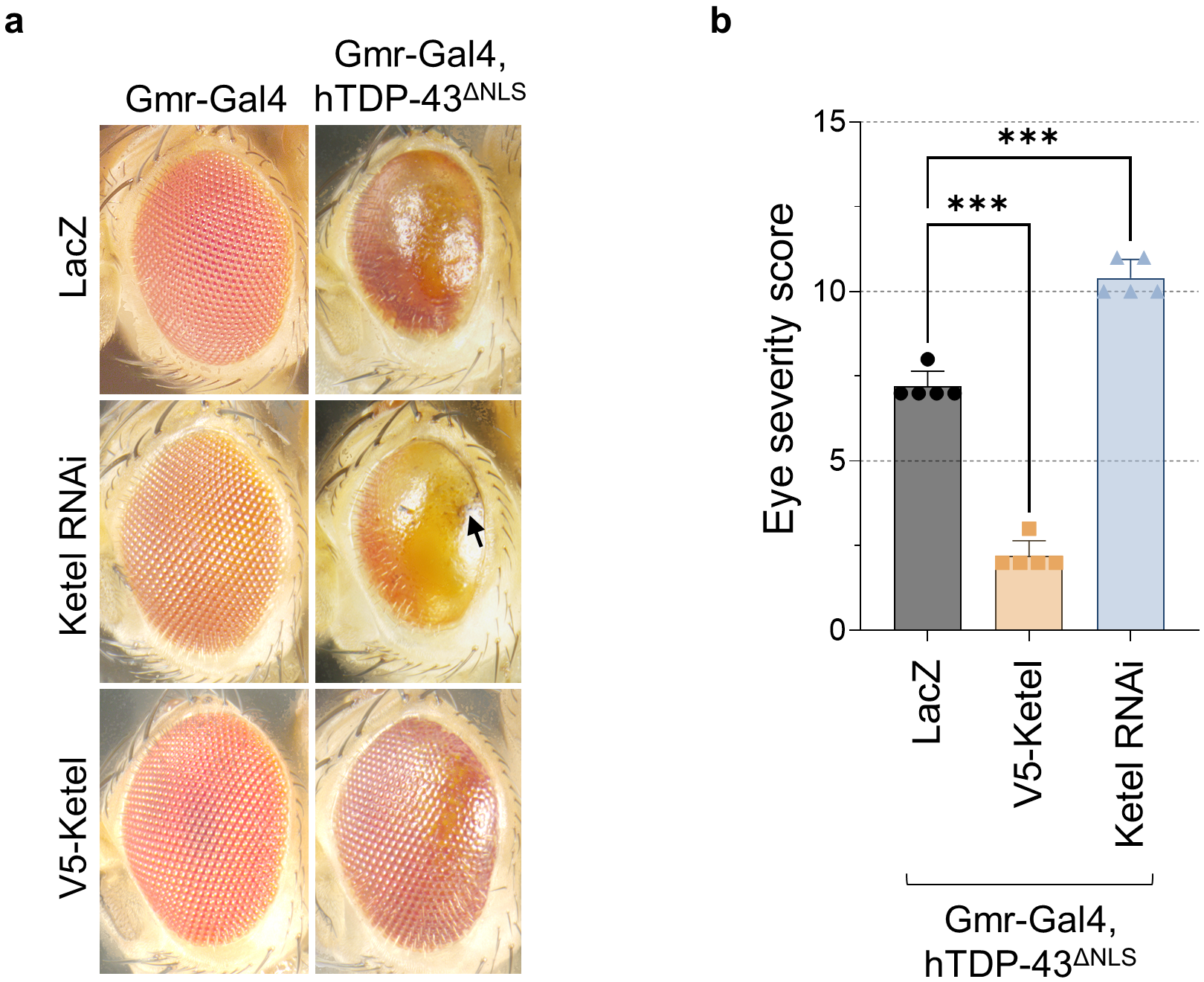
**

**Supplementary Fig. 16** Downregulation of Ketel exacerbates toxicity of hTDP-43^ΔNLS^ in the fly eye, but Ketel overexpression alleviates it. **a,** Eye phenotypes from flies expressing the indicated transgenes via the eye-specific Gmr-Gal4 driver. Arrow points to necrotic areas. **b,** Quantitative analysis of eye severity in flies co-expressing hTDP-43^ΔNLS^ and the indicated transgenes via the eye-specific Gmr-Gal4 driver. Statistical analysis was performed using one-way ANOVA and Bonferroni’s post hoc test (***p<0.001, n=5).


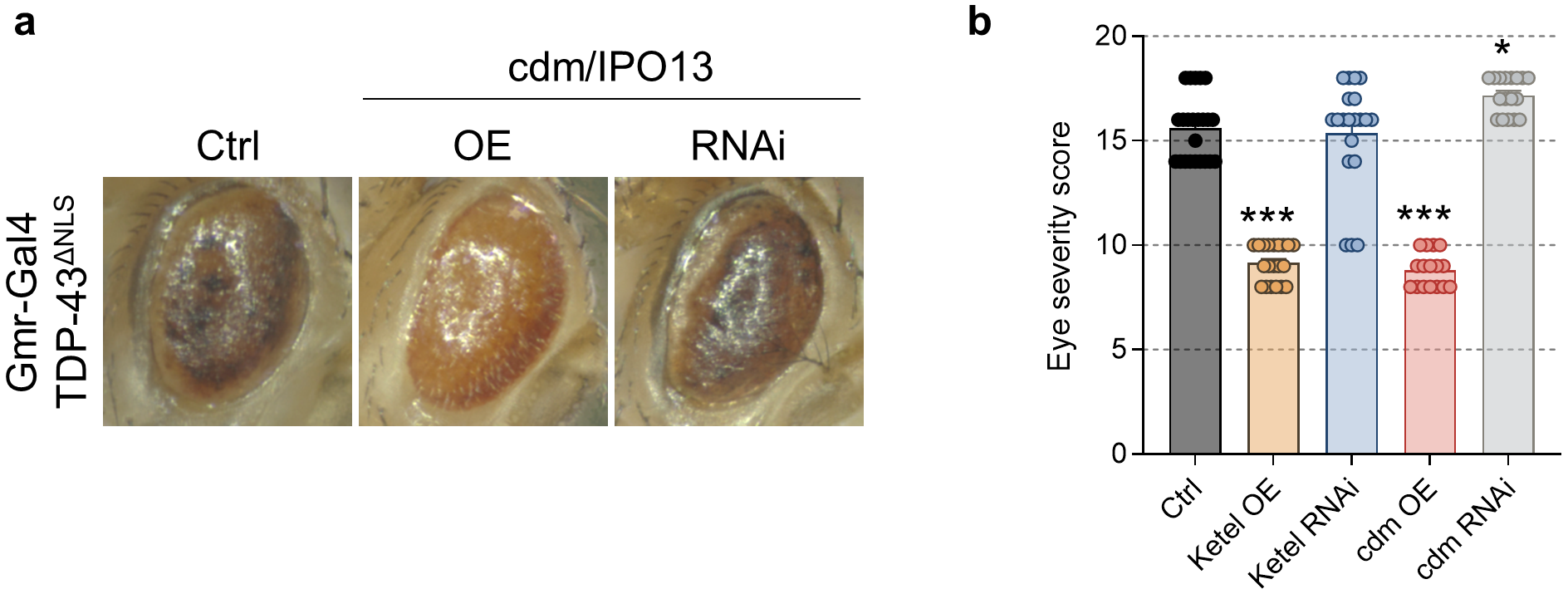


**Supplementary Fig. 17** The NIR cdm/IPO13 rescues severe eye degeneration in hTDP-43^ΔNLS^ flies. **a,** Eye phenotypes from flies expressing the indicated transgenes via the eye-specific Gmr-Gal4 driver. cdm overexpression (OE) rescues hTDP-43-induced neurodegeneration. **b,** Quantitative analysis of eye severity in flies co-expressing hTDP-43^ΔNLS^ and the indicated transgenes via the eye-specific Gmr-Gal4 driver. Statistical analysis was performed using one-way ANOVA and Bonferroni’s post hoc test (*p<0.05, ***p<0.001, n=16-23 flies per group).


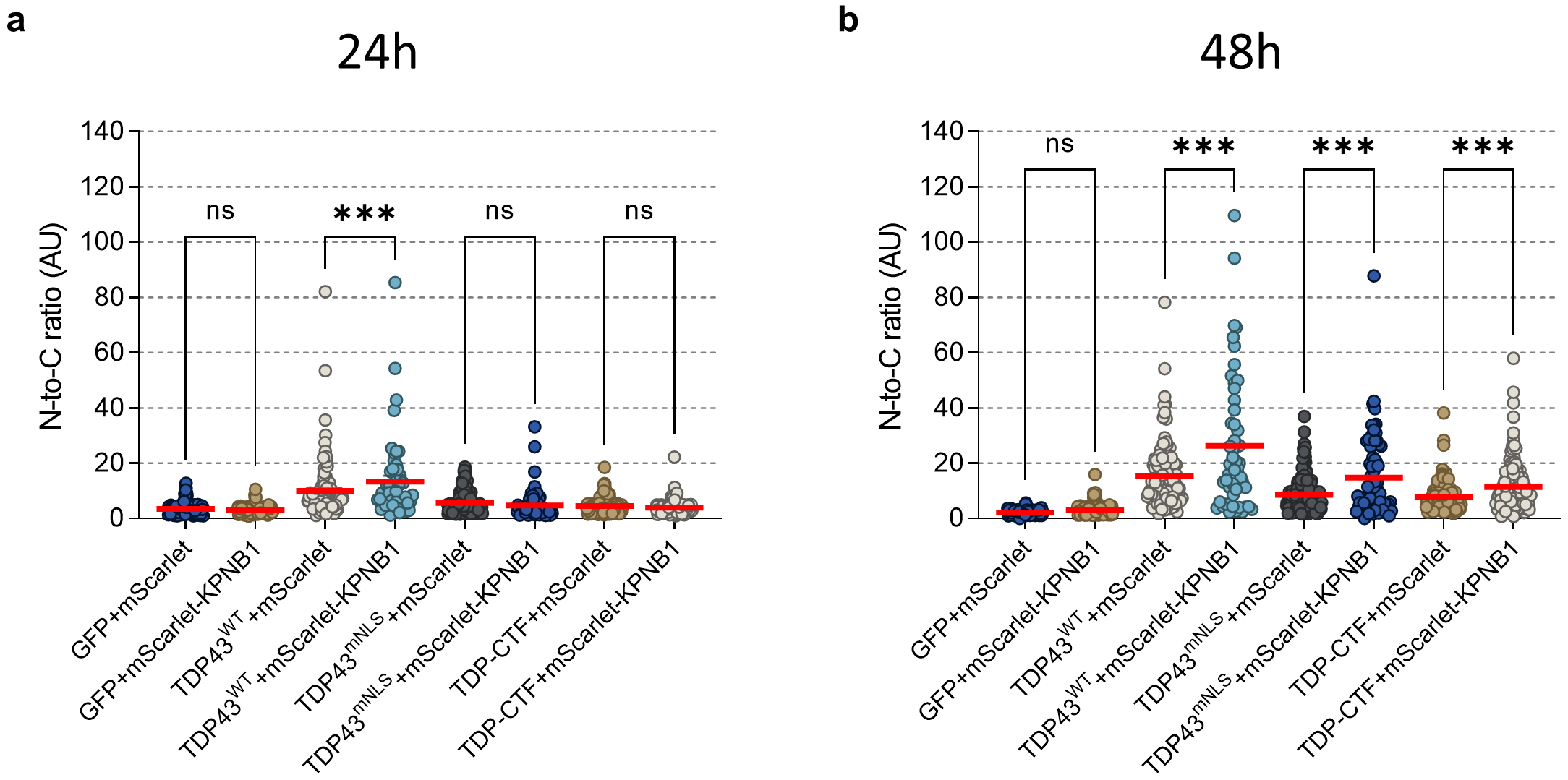


**Supplementary Fig. 18** KPNB1 increases the N-to-C ratio of different TDP-43 constructs in rodent primary neurons. Quantitative analysis of the N-to-C ratio of GFP or GFP-tagged TDP-43^WT^, TDP-43^mNLS^ or TDP-CTF in primary neurons expressing mScarlet or mScarlet-KPNB1 24 h **(a)** or 48 h **(b)** post-transfection. Statistical analysis was performed using one-way ANOVA and Bonferroni’s post hoc test (***p<0.001, n=52-264 neurons per group).


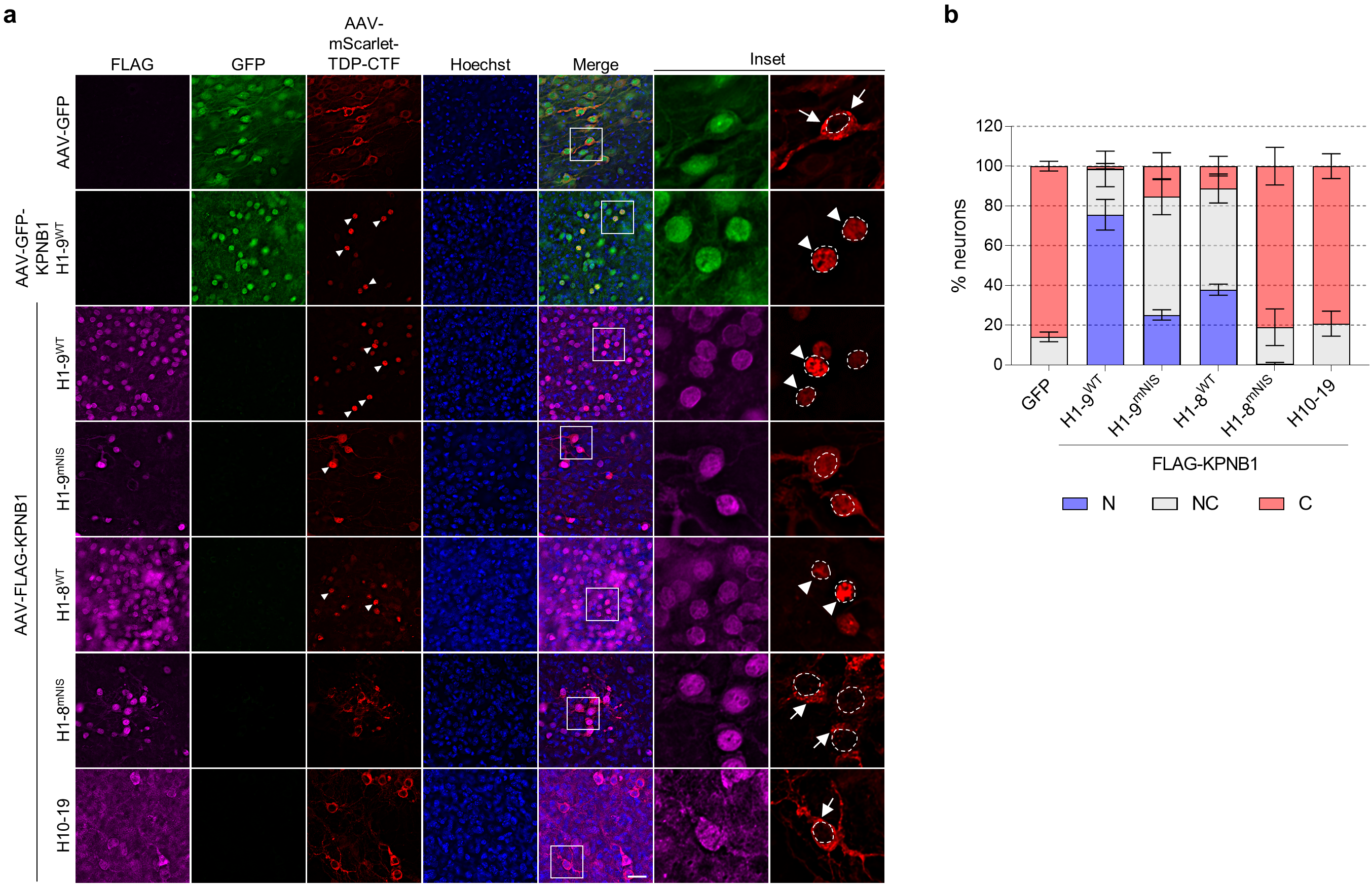


**Supplementary Fig. 19** KPNB1 reduces cytoplasmic aggregation of TDP-CTF and promotes nuclear localization in BSCs. **a**, Immunofluorescence of mouse BSCs (DIV12) co-expressing AAV-mScarlet-TDP-CTF with AAV-GFP, AAV-GFP-KPNB1 H1-9^WT^, AAV-FLAG-KPNB1 H1-9^WT^, H1-9^mNIS^, H1-8^WT^, H1-8^mNIS^ or H10-19. KPNB1 H1-9^WT^ and H1-8^WT^ reduce TDP-CTF cytoplasmic aggregates (arrows) and render it very nuclear (arrowheads), whereas NIS mutations strongly reduce this function. Hoechst staining was used to outline nuclei. Scale bar: 50 μm. **b,** Quantification of the percentage of neurons exhibiting nuclear (N), nucleocytoplasmic (NC) or cytoplasmic (C) mScarlet-TDP-CTF distribution upon expression of different AAV-KPNB1 constructs. Statistical analysis was performed using two-way ANOVA and Bonferroni’s post hoc test (three independent experiments; n=150-178 neurons per group; statistics are summarized in Supplementary Table 2).

**
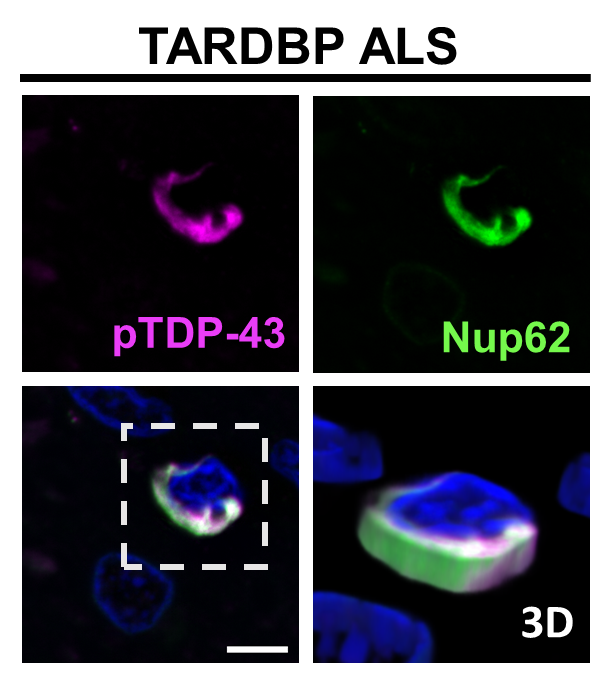
**

**Supplementary Fig. 20** Nup62 colocalizes with pTDP-43 aggregates in the spinal cord of TARDBP ALS case. High resolution co-immunofluorescence staining of pTDP-43 (magenta) and Nup62 (green) with Hoechst (blue) was conducted in fixed spinal cord of a neuropathologically diagnosed TARDBP ALS case. pTDP-43 was stained with a 647/Cy5 secondary antibody and Nup62 with a 488/FITC antibody to ensure there is no bleed through between channels. The image is shown as a panel of projected optical sections from each z-series for magenta and green channels and merged with Hoechst DNA staining. Volume rendered z-series of all channels are shown as the 3D inset. Scale bar: 5 µm.


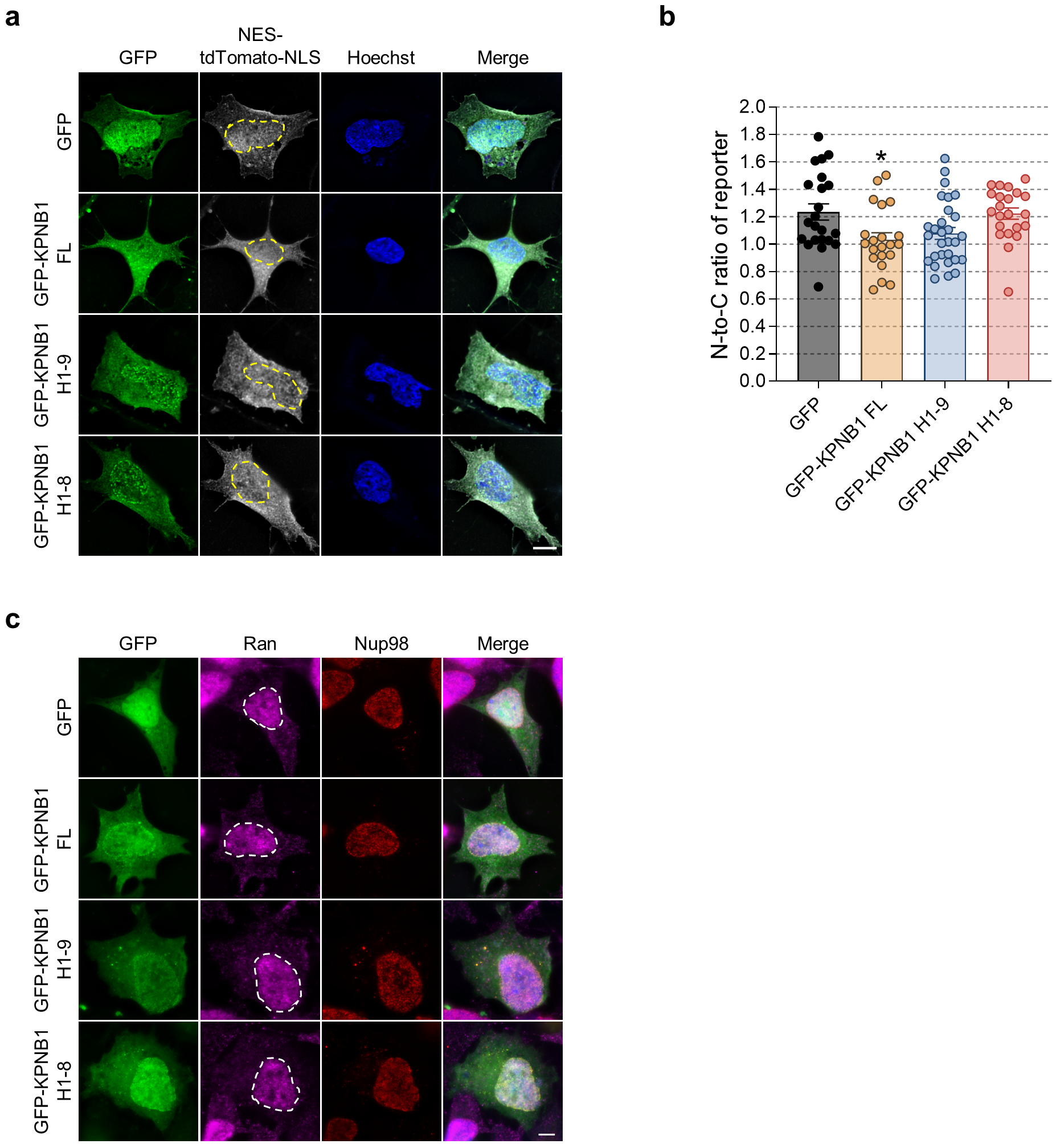


**Supplementary Fig. 21** KPNB1 expression does not dysregulate nucleocytoplasmic transport. **a,** Immunofluorescence (IF) of SH-SY5Y cells co-expressing the transport reporter NES-tdTomato-NLS with GFP or GFP-tagged KPNB1 full-length (FL), H1-9 or H1-8. Hoechst staining was used to outline nuclei. Scale bar: 5 μm. **b,** Quantification of the N-to-C ratio of the NES-tdTomato-NLS reporter. Statistical analysis was performed using one-way ANOVA and Bonferroni’s post hoc test (*p<0.05, n=21-29 cells per group). **c,** IF of SH-SY5Y cells expressing GFP or GFP-tagged full-length KPNB1 (FL), H1-9 or H1-8 and stained for endogenous Ran and Nup98. Both proteins remain mostly nuclear in presence of KPNB1. Hoechst staining was used to outline nuclei. Scale bar: 5 μm.

**
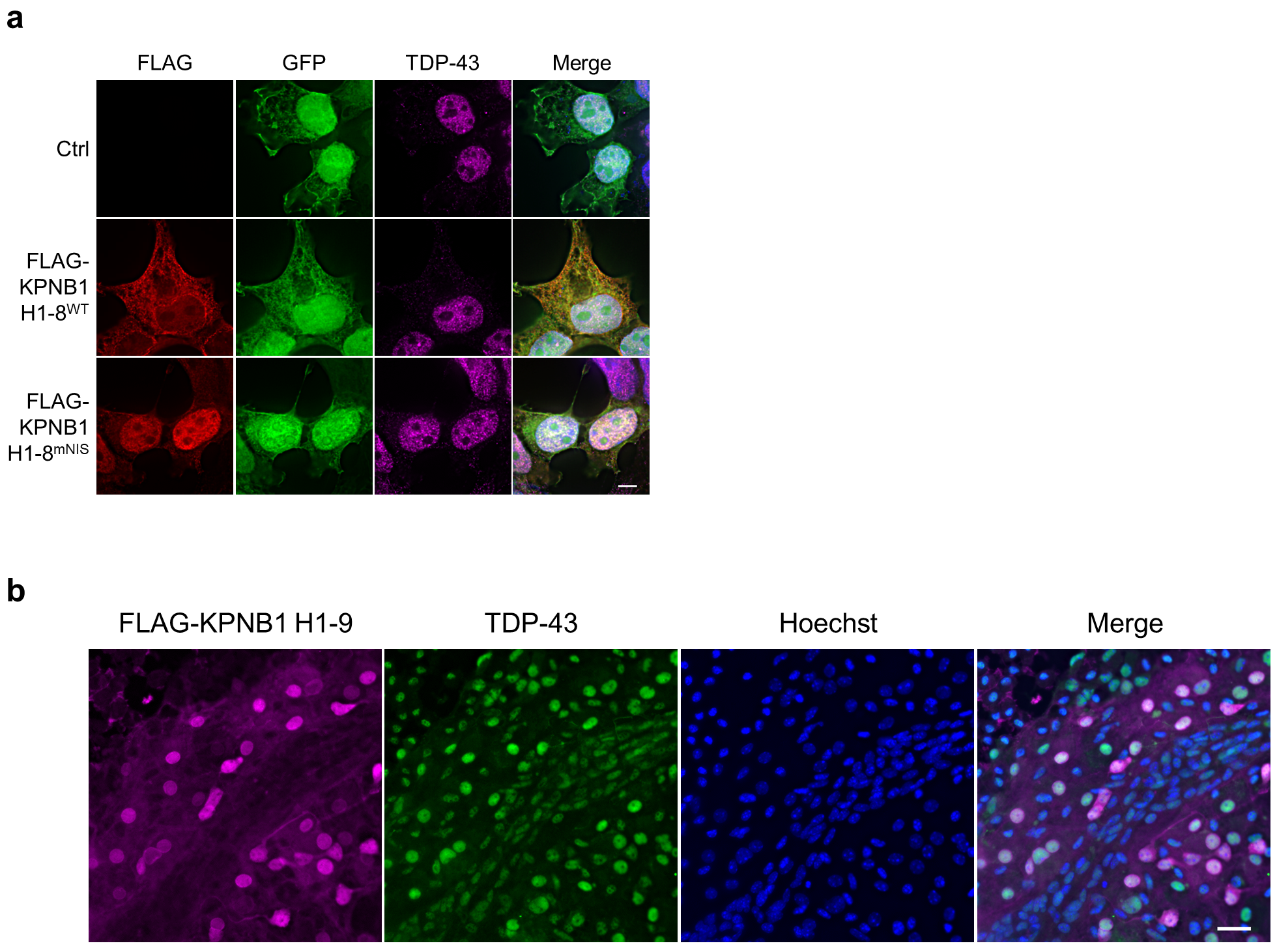
**

**Supplementary Fig. 22** Expression of the N-terminal half of KPNB1 does not affect the nuclear localization of endogenous TDP-43 in cells and BSCs. **a,** Immunofluorescence (IF) of HEK293T cells co-expressing GFP with an empty plasmid (ctrl), FLAG-tagged KPNB1 H1-8^WT^ or H1-8^mNIS^ and stained for endogenous TDP-43. TDP-43 remains nuclear in presence of KPNB1 H1-8. Scale bar: 5 μm. **b**, IF of mouse BSCs (DIV15) expressing AAV-FLAG-KPNB1 H1-9 and stained for endogenous TDP-43. TDP-43 remains nuclear in presence of KPNB1 H1-9. Scale bar: 25 μm.

**
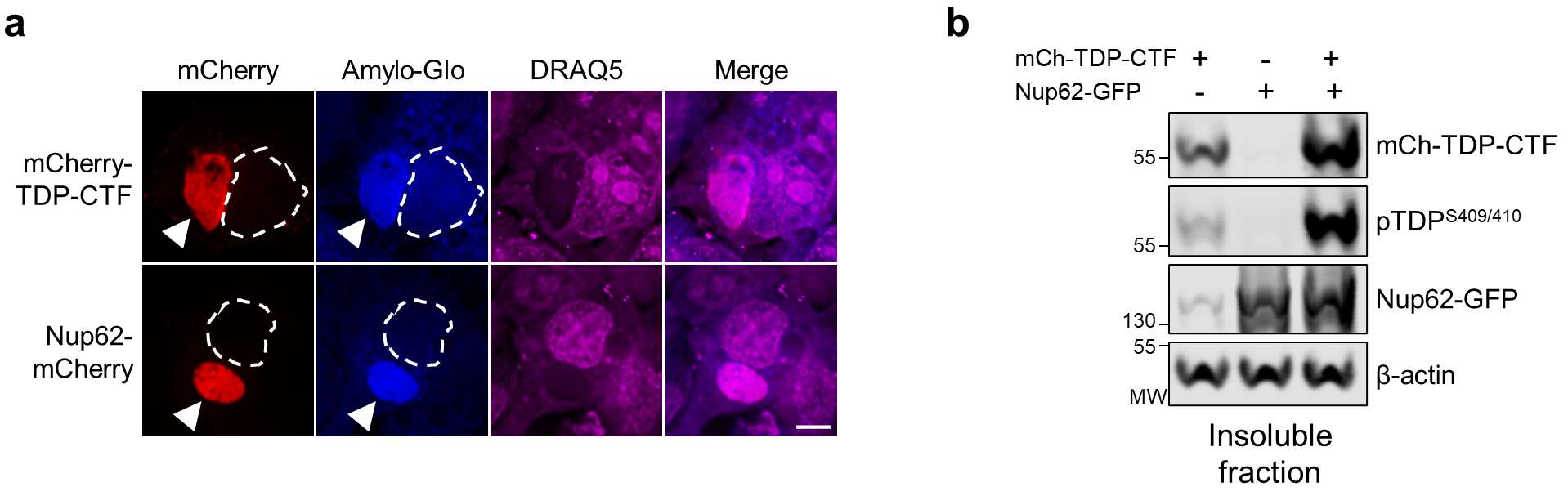
**

**Supplementary Fig. 23** Nup62 and TDP-CTF aggregates are positive for amyloid staining and their co-expression promotes TDP-CTF aggregation. **a,** Immunofluorescence of HEK293T cells expressing mCherry-TDP-CTF or Nup62-mCherry which form Amylo-Glo-positive aggregates. DRAQ5 staining was used to outline nuclei. Scale bar: 5 μm. **b,** Western blot analysis of insoluble mCherry-TDP-CTF in HEK293T cells expressing GFP or Nup62-GFP. Nup62 strongly increases insoluble levels of TDP-CTF.

**Supplementary Tables**

**Supplementary Table 1.** Demographic information of human post-mortem cases used for Nup62 and KPNB1 co-immunostaining with pTDP-43^S409/410^.

| Case ID | Clinical Dx | Prim.path  Dx | Age | Sex | Genetics | TDP-43 subtype | Braak  stage | Thal phase | Brain weight (g) | CNS region |
| --- | --- | --- | --- | --- | --- | --- | --- | --- | --- | --- |
| C9/ALS #1 | ALS | ALS-C9 | 66 | M | C9orf72 | B | II | 0 | 1320 | SC + Mot. ctx |
| C9/ALS #2 | ALS | ALS-C9 | 67 | F | C9orf72 | B | II | 0 | 1120 | SC + Mot. ctx |
| sALS #1 | ALS | ALS | 76 | M | - | B | III | 0 | 1340 | SC + Mot. ctx |
| sALS #2 | ALS | ALS | 58 | F | - | B | 0 | 0 | 1260 | SC + Mot. ctx |
| SOD1/ALS #1 | ALS | ALS-SOD1 | 66 | F | SOD1**^#^** | - | 0 | 0 | 1360 | SC + Mot. ctx |
| SOD1/ALS #2 | ALS | ALS-SOD1 | 48 | M | SOD* | - | 0 | 0 | 1260 | SC + Mot. ctx |
| FUS/ALS | ALS | ALS-FUS | 68 | F | undetermined | - | II | 0 | 1410 | SC + Mot. ctx |
| TARDBP/ALS | ALS | ALS | 67 | M | TARDBP‡ | - | I | 0 | 1080 | SC + Mot. ctx |
| Control #1 | MSA | Normal | 77 | M | - | - | II | 0 | 1000 | SC + Mot. ctx |
| Control #2 | Normal | Normal | 64 | M | - | - | II | 0 | 1420 | SC + Mot. ctx |
| C9/FTLD-A #1 | FTD | FTLD-C9 | 69 | F | C9orf72 | A | III | 0 | 880 | HP+ Mot. ctx |
| C9/FTLD-A #2 | FTD | FTLD-C9 | 69 | M | C9orf72 | A | IV | 0 | 1320 | HP+ Mot. ctx |
| C9/FTLD-B #1 | FTD | FTLD-C9 | 77 | M | C9orf72 | B | II | 0 | 1300 | HP+ Mot. ctx |
| C9/FTLD-B#2 | FTD | FTLD-C9 | 62 | M | C9orf72 | B | II | 0 | 1060 | HP+ Mot. ctx s |
| sFTLD-A #1 | FTD | FTLD | 70 | F | - | A | II | 3 | 1040 | HP+ Mot. ctx |
| sFTLD-A #2 | FTD | FTLD | 76 | F | - | A | 0 | 0 | 1040 | HP+ Mot. ctx |
| sFTLD-B #1 | DLB | FTLD | 70 | M | - | B | III | 0 | 1200 | HP+ Mot. ctx |
| sFTLD B#2 | PSP | FTLD | 62 | F | - | B | III | 0 | 1260 | HP+ Mot. ctx |
| Control #3 | Normal | Normal | 58 | M | - | - | II | 0 | 1280 | HP+ Mot. ctx |
| Control #4 | Depression | Normal | 59 | M | - | - | 0 | 0 | 1540 | HP+ Mot. ctx |

Abbreviations: *C9orf72/C9*, chromosome 9 open reading frame 72; ALS, amyotrophic lateral sclerosis; sALS, sporadic ALS; FTD, frontotemporal dementia; FTLD, frontotemporal lobar degeneration; sFTLD, sporadic FTLD; MSA, multisystems atrophy;TDP-43, transactive response DNA-binding protein-43 (*TARDBP*); SOD1, superoxide dismutase 1; FUS, fused in sarcoma; DLB, dementia with Lewy bodies; PSP, progressive supranuclear palsy; Prim., primary; path, pathological; Dx, diagnosis; CNS, central nervous system; SC, spinal cord; Mot. Ctx, primary motor cortex; HP, hippocampus; M, male; F, female. **^#^** SOD1c.341 T>C; * SOD1p.A4V; p.I113T; ‡ TARDBPc.892G>A; p.Gly298Ser

**Supplementary Table 2.** Summary of statistical analyses.

| Fig. 1b - α-importin screen | | |
| --- | --- | --- |
| Test used | One-way ANOVA; Bonferroni’s post hoc test | Three independent experiments |
| F (DFn, DFd) | F (8, 18) = 5.73 |  |
| P Value | p = 0.001 |  |
| Post hoc test | GFP vs GFP-KPNB1  GFP vs GFP-KPNA1  GFP vs GFP-KPNA2  GFP vs GFP-KPNA3  GFP vs GFP-KPNA4  GFP vs GFP-KPNA5  GFP vs GFP-KPNA6  GFP vs GFP-KPNA7 | p < 0.001  p = 0.308  p = 0.133  p > 0.999  p > 0.999  p = 0.711  p > 0.999  p > 0.999 |
| Fig. 1b - β-importin screen | | |
| Test used | One-way ANOVA; Bonferroni’s post hoc test | Three independent experiments |
| F (DFn, DFd) | F (11, 24) = 7.863 |  |
| P Value | p < 0.001 |  |
| Post hoc test | GFP vs GFP-KPNB1  GFP vs GFP-TNPO1  GFP vs GFP-TNPO2A  GFP vs GFP-TNPO2B  GFP vs GFP-IPO4  GFP vs GFP-IPO7  GFP vs GFP-IPO8  GFP vs GFP-IPO9  GFP vs GFP-IPO11  GFP vs GFP-TNPO3  GFP vs GFP-IPO13 | p = 0.008  p > 0.999  p = 0.006  p = 0.005  p = 0.011  p = 0.204  p > 0.999  p = 0.006  p = 0.432  p = 0.027  p < 0.001 |
| Fig. 1b - exportin screen | | |
| Test used | One-way ANOVA; Bonferroni’s post hoc test | Three independent experiments |
| F (DFn, DFd) | F (10, 22) = 6.17 |  |
| P Value | p < 0.001 |  |
| Post hoc test | GFP vs GFP-KPNB1  GFP vs GFP-XPO1  GFP vs GFP-XPO2  GFP vs GFP-XPOT  GFP vs GFP-XPO4  GFP vs GFP-XPO5  GFP vs GFP-XPO6  GFP vs GFP-XPO7  GFP vs GFP-RANBP6  GFP vs GFP-RANBP17 | p = 0.003  p > 0.999  p > 0.999  p > 0.999  p > 0.999  p = 0.332  p = 0.335  p > 0.999  p > 0.999  p > 0.999 |
| Fig. 2b | | |
| Test used | One-way ANOVA; Bonferroni’s post hoc test | Four independent experiments |
| F (DFn, DFd) | F (3, 12) = 22.3 |  |
| P Value | p < 0.001 |  |
| Post hoc test | GFP vs GFP-KPNB1 FL  GFP vs GFP-KPNB1 H1-9  GFP vs GFP-KPNB1 H10-19 | p = 0.004  p < 0.001  p > 0.999 |
| Fig. 2c | | |
| Test used | Two-way ANOVA; Bonferroni’s post hoc test | Four independent experiments |
| F (DFn, DFd) | F (3, 24) = 17  F (3, 24) = 12.9  F (1, 24) = 193 |  |
| P Value | p < 0.001  p < 0.001  p < 0.001 |  |
| Post hoc test: mCherry | GFP vs GFP-KPNB1 FL  GFP vs GFP-KPNB1 H1-9  GFP vs GFP-KPNB1 H10-19 | p > 0.999  p > 0.999  p > 0.999 |
| Post hoc test: mCherry-TDP-CTF | GFP vs GFP-KPNB1 FL  GFP vs GFP-KPNB1 H1-9  GFP vs GFP-KPNB1 H10-19 | p < 0.001  p < 0.001  p > 0.999 |
| Fig. 2e | | |
| Test used | One-way ANOVA; Bonferroni’s post hoc test | Four independent experiments |
| F (DFn, DFd) | F (10, 33) = 7.656 |  |
| P Value | p < 0.001 |  |
| Post hoc test | GFP vs GFP-KPNB1 FL  GFP vs GFP-KPNB1 H1-9  GFP vs GFP-KPNB1 H1-8  GFP vs GFP-KPNB1 H1-7  GFP vs GFP-KPNB1 H1-6  GFP vs GFP-KPNB1 H1-5  GFP vs GFP-KPNB1 H1-4  GFP vs GFP-KPNB1 H1-3  GFP vs GFP-KPNB1 H1-2  GFP vs GFP-KPNB1 H1 | p = 0.006  p < 0.001  p < 0.001  p = 0.005  p = 0.04  p = 0.480  p > 0.999  p > 0.999  p > 0.999  p = 0.106 |
| Fig. 3e | | |
| Test used | One-way ANOVA; Bonferroni’s post hoc test | Four independent experiments |
| F (DFn, DFd) | F (2, 39) = 81.66 |  |
| P Value | p < 0.001 |  |
| Post hoc test | GFP vs GFP-KPNB1 H1-8^WT^  GFP vs GFP-KPNB1 H1-8^mNIS^  GFP-KPNB1 H1-8^WT^ vs GFP-KPNB1 H1-8^mNIS^ | p < 0.001  p = 0.003  p < 0.001 |
| Fig. 3g | | |
| Test used | One-way ANOVA; Bonferroni’s post hoc test | Three independent experiments |
| n | Ctrl  FLAG-KPNB1 H1-8^WT^  FLAG-KPNB1 H1-8^mNIS^ | n=150  n=150  n=153 |
| F (DFn, DFd) | F (2, 6) = 327.3 |  |
| P Value | p < 0.001 |  |
| Post hoc test | Ctrl vs FLAG-KPNB1 H1-8^WT^  Ctrl vs FLAG-KPNB1 H1-8^mNIS^  FLAG-KPNB1 H1-8^WT^ vs FLAG-KPNB1 H1-8^mNIS^ | p < 0.001  p = 0.015  p < 0.001 |
| Fig. 3i | | |
| Test used | One-way ANOVA; Bonferroni’s post hoc test | Three independent experiments |
| n | Ctrl  FLAG-KPNB1 H1-8^WT^  FLAG-KPNB1 H1-8^mNIS^ | n=150  n=151  n=151 |
| F (DFn, DFd) | F (2, 6) = 168.6 |  |
| P Value | p < 0.001 |  |
| Post hoc test | Ctrl vs FLAG-KPNB1 H1-8^WT^  FLAG-KPNB1 H1-8^WT^ vs FLAG-KPNB1 H1-8^mNIS^ | p < 0.001  p < 0.001 |
| Fig. 4b | | |
| Test used | Two-way ANOVA; Bonferroni’s post hoc test | Three independent experiments |
| F (DFn, DFd) | F (2, 12) = 17.4  F (1, 12) = 250  F (2, 12) = 17.4 |  |
| P Value | p < 0.001  p < 0.001  p < 0.001 |  |
| Post hoc test | GFP-TDP-CTF: mCherry vs mCherry-KPNB1  GFP-TDP-43^mNLS^: mCherry vs mCherry-KPNB1  GFP-sTDP: mCherry vs mCherry-KPNB1  mCherry-KPNB1: GFP-TDP-CTF vs GFP-sTDP  mCherry-KPNB1: GFP-sTDP vs GFP-TDP-43^mNLS^ | p < 0.001  p = 0.013  p < 0.001  p < 0.001  p < 0.001 |
| Fig. 4g - dissolution of aggregation | | |
| Test used | One-way ANOVA; Bonferroni’s post hoc test | Three independent experiments |
| F (DFn, DFd) | F (9, 20) = 37 |  |
| P Value | p < 0.001 |  |
| Post hoc test:  TDP-CTF | BSA vs KPNB1 WT  BSA vs KPNB1 mNIS  BSA vs KPNB1 H1-9^WT^  BSA vs KPNB1 H1-9^mNIS^ | p < 0.001  p = 0.012  p < 0.001  p = 0.014 |
| Post hoc test:  TDP-CTF+Nup62FG | BSA vs KPNB1 WT  BSA vs KPNB1 mNIS  BSA vs KPNB1 H1-9^WT^  BSA vs KPNB1 H1-9^mNIS^  KPNB1 WT vs KPNB1 mNIS  KPNB1 H1-9^WT^ vs KPNB1 H1-9^mNIS^  BSA (TDP-CTF) vs BSA (TDP-CTF+Nup62FG) | p < 0.001  p = 0.009  p < 0.001  p = 0.001  p < 0.001  p < 0.001  p = 0.002 |
| Fig. 4g - prevention of aggregation | | |
| Test used | One-way ANOVA; Bonferroni’s post hoc test | Three independent experiments |
| F (DFn, DFd) | F (9, 20) = 79.4 |  |
| P Value | p < 0.001 |  |
| Post hoc test:  TDP-CTF | BSA vs KPNB1 WT  BSA vs KPNB1 mNIS  BSA vs KPNB1 H1-9^WT^  BSA vs KPNB1 H1-9^mNIS^ | p < 0.001  p = 0.002  p < 0.001  p = 0.002 |
| Post hoc test:  TDP-CTF+Nup62FG | BSA vs KPNB1 WT  BSA vs KPNB1 H1-9^WT^  KPNB1 WT vs KPNB1 mNIS  KPNB1 H1-9^WT^ vs KPNB1 H1-9^mNIS^  BSA (TDP-CTF) vs BSA (TDP-CTF+Nup62FG) | p < 0.001  p < 0.001  p < 0.001  p < 0.001  p < 0.001 |
| Fig. 5a | | |
| Test used | One-way ANOVA; Bonferroni’s post hoc test | n≥4 |
| F (DFn, DFd) | F (8, 41) = 53.85 |  |
| P Value | p < 0.001 |  |
| Post hoc test: LacZ | GG4 vs TDP-43^WT^  GG4 vs TDP-43^M337V^ | p > 0.999  p < 0.001 |
| Post hoc test: GG4 | LacZ vs Ketel RNAi  LacZ vs V5-Ketel | p = 0.445  p > 0.999 |
| Post hoc test:  TDP-43^WT^ | LacZ vs Ketel RNAi  LacZ vs V5-Ketel | p < 0.001  p > 0.999 |
| Post hoc test:  TDP-43^M337V^ | LacZ vs Ketel RNAi  LacZ vs V5-Ketel | p = 0.002  p < 0.001 |
| Fig. 5c | | |
| Test used | Two-way ANOVA; Bonferroni’s post hoc test | n=13 |
| F (DFn, DFd) | F (2, 72) = 34.3  F (2, 72) = 24.92  F (1, 72) = 58.76 |  |
| P Value | p < 0.001  p < 0.001  p < 0.001 |  |
| Post hoc test:  No Drug | LacZ vs Ketel RNAi  LacZ vs V5-Ketel | p = 0.077  p = 0.121 |
| Post hoc test: RU486 | LacZ vs Ketel RNAi  LacZ vs V5-Ketel | p > 0.999  p < 0.001 |
| Fig. 5d | | |
| Test used | OASIS tool; Fisher’s exact test | n=80 |
|  | TDP-43^M337V^: LacZ vs Ketel RNAi  TDP-43^M337V^: LacZ vs V5-Ketel | p < 0.01  p < 0.0001 |
| Fig. 6b | | |
| Test used | One-way ANOVA; Bonferroni’s post hoc test | Two independent experiments, eight technical replicates per experiment  n=52-264 |
| F (DFn, DFd) | F (7, 1381) = 109.2 |  |
| P Value | p < 0.001 |  |
| Post hoc test | GFP: mScarlet vs mScarlet-KPNB1  TDP-43^WT^: mScarlet vs mScarlet-KPNB1  TDP-43^mNLS^: mScarlet vs mScarlet-KPNB1  TDP-CTF: mScarlet vs mScarlet-KPNB1 | p > 0.999  p < 0.001  p < 0.001  p < 0.001 |
| Fig. 6d | | |
| Test used | Two-way ANOVA; Bonferroni’s post hoc test | Three independent experiments |
| n | mCherry  mCherry-KPNB1 FL  mCherry-KPNB1 H1-9  mCherry-KPNB1 H1-8^WT^  mCherry-KPNB1 H1-8^mNIS^  mCherry-KPNB1 H10-19 | n=79  n=66  n=81  n=76  n=80  n=74 |
| F (DFn, DFd) | F (10, 36) = 7.067  F (5, 36) = 9.552e-016  F (2, 36) = 54.45 |  |
| P Value | p < 0.001  p > 0.999  p < 0.001 |  |
| Post hoc test: cytoplasmic  GFP-TDP-43^mNLS^ | mCherry vs mCherry-KPNB1 H1-9^WT^  mCherry vs mCherry-KPNB1 H1-8^WT^  mCherry-KPNB1 H1-8^WT^ vs mCherry-KPNB1 H1-8^mNIS^ | p = 0.002  p < 0.001  p = 0.002 |
| Post hoc test: nucleocytoplasmic  GFP-TDP-43^mNLS^ | mCherry vs mCherry-KPNB1 H1-9^WT^  mCherry vs mCherry-KPNB1 H1-8^WT^  mCherry-KPNB1 H1-8^WT^ vs mCherry-KPNB1 H1-8^mNIS^ | p = 0.009  p = 0.001  p = 0.016 |
| Fig. 6f | | |
| Test used | Two-way ANOVA; Bonferroni’s post hoc test | Three independent experiments |
| n | FLAG-mCherry  FLAG-KPNB1 H1-9^WT^  FLAG-KPNB1 H1-9^mNIS^  FLAG-KPNB1 H1-8^WT^  FLAG-KPNB1 H1-8^mNIS^  FLAG-KPNB1 H10-19 | n=150  n=152  n=151  n=156  n=151  n=154 |
| F (DFn, DFd) | F (10, 36) = 152  F (5, 36) = 1.183e-007  F (2, 36) = 148.8 |  |
| P Value | p < 0.001  p > 0.999  p < 0.001 |  |
| Post hoc test: cytoplasmic  GFP-TDP-43^mNLS^ | FLAG-mCherry vs FLAG-KPNB1 H1-9^WT^  FLAG-mCherry vs FLAG-KPNB1 H1-9^mNIS^  FLAG-mCherry vs FLAG-KPNB1 H1-8^WT^  FLAG-KPNB1 H1-9^WT^ vs FLAG-KPNB1 H10-19  FLAG-KPNB1 H1-8^WT^ vs FLAG-KPNB1 H1-8^mNIS^ | p < 0.001  p < 0.001  p < 0.001  p < 0.001  p < 0.001 |
| Post hoc test: nucleocytoplasmic  GFP-TDP-43^mNLS^ | FLAG-mCherry vs FLAG-KPNB1 H1-9^WT^  FLAG-mCherry vs FLAG-KPNB1 H1-9^mNIS^  FLAG-mCherry vs FLAG-KPNB1 H1-8^WT^  FLAG-KPNB1 H1-9^WT^ vs FLAG-KPNB1 H1-9^mNIS^  FLAG-KPNB1 H1-9^WT^ vs FLAG-KPNB1 H10-19  FLAG-KPNB1 H1-8^WT^ vs FLAG-KPNB1 H1-8^mNIS^ | p < 0.001  p < 0.001  p < 0.001  p < 0.001  p < 0.001  p < 0.001 |
| Post hoc test:  nuclear  GFP-TDP-43^mNLS^ | FLAG-mCherry vs FLAG-KPNB1 H1-9^WT^  FLAG-mCherry vs FLAG-KPNB1 H1-8^WT^  FLAG-KPNB1 H1-9^WT^ vs FLAG-KPNB1 H1-9^mNIS^  FLAG-KPNB1 H1-9^WT^ vs FLAG-KPNB1 H10-19  FLAG-KPNB1 H1-8^WT^ vs FLAG-KPNB1 H1-8^mNIS^ | p < 0.001  p < 0.001  p < 0.001  p < 0.001  p < 0.001 |
| Supplementary Fig. 1a | | |
| Test used | Unpaired t test | Ten independent experiments |
| t(df) | t(18) = 15.63 (TDP-CTF)  t(18) = 0.1473 (endog. TDP-43) |  |
| P Value | p < 0.001 (TDP-CTF)  p = 0.884 (endog. TDP-43) |  |
| Supplementary Fig. 1d | | |
| Test used | Two-way ANOVA; Bonferroni’s post hoc test | Three independent experiments |
| F (DFn, DFd) | F (3, 16) = 0.4236  F (1, 16) = 174.7  F (3, 16) = 0.4236 |  |
| P Value | p = 0.739  p < 0.001  p = 0.739 |  |
| Post hoc test | TDP-CTF^WT^: mCherry vs mCherry-KPNB1  TDP-CTF^Q331K^: mCherry vs mCherry-KPNB1  TDP-CTF^M337V^: mCherry vs mCherry-KPNB1  TDP-CTF^A382T^: mCherry vs mCherry-KPNB1 | p < 0.001  p < 0.001  p < 0.001  p < 0.001 |
| Supplementary Fig. 1e | | |
| Test used | One-way ANOVA; Bonferroni’s post hoc test | Four independent experiments |
| F (DFn, DFd) | F (2, 9) = 17.3 (TDP-CTF)  F (2, 9) = 4.301 (TDP-43) |  |
| P Value | p < 0.001 (TDP-CTF)  p = 0.049 (TDP-43) |  |
| Post hoc test:  TDP-CTF | mCherry vs mCherry-KPNB1  mCherry vs untagged KPNB1 | p = 0.011  p < 0.001 |
| Post hoc test:  TDP-43 | mCherry vs mCherry-KPNB1  mCherry vs untagged KPNB1 | p = 0.056  p > 0.999 |
| Supplementary Fig. 1f | | |
| Test used | One-way ANOVA; Bonferroni’s post hoc test | Three independent experiments |
| F (DFn, DFd) | F (2, 6) = 0.2436 (TDP-CTF)  F (2, 6) = 0.09048 (TDP-43) |  |
| P Value | p = 0.791 (TDP-CTF)  p = 0.915 (TDP-43) |  |
| Post hoc test | mCherry vs mCherry-KPNB1  mCherry vs untagged KPNB1 | p > 0.999  p > 0.999 |
| Supplementary Fig. 2 | | |
| Test used | One-way ANOVA; Bonferroni’s post hoc test | Four independent experiments |
| F (DFn, DFd) | F (3, 12) = 4.705 |  |
| P Value | p = 0.021 |  |
| Post hoc test | GFP vs GFP-KPNB1 FL  GFP vs GFP-KPNB1 H1-9  GFP vs GFP-KPNB1 H10-19 | p > 0.999  p > 0.999  p = 0.265 |
| Supplementary Fig. 4a | | |
| Test used | One-way ANOVA; Bonferroni’s post hoc test | Three independent experiments |
| F (DFn, DFd) | F (2, 6) = 38.41 |  |
| P Value | p < 0.001 |  |
| Post hoc test | GFP vs GFP-TDP-CTF  GFP-TDP-CTF vs GFP-TDP-43 | p < 0.001  p = 0.002 |
| Supplementary Fig. 4c | | |
| Test used | One-way ANOVA; Bonferroni’s post hoc test | Three independent experiments |
| F (DFn, DFd) | F (3, 8) = 27.4 |  |
| P Value | p < 0.001 |  |
| Post hoc test | GFP vs GFP-TDP-CTF  GFP vs GFP-TDP-CTF^non-PrLD^  GFP vs GFP-TDP-PrLD | p < 0.01  p < 0.05  p < 0.05 |
| Supplementary Fig. 5d | | |
| Test used | Unpaired t test | Three independent experiments |
| t(df) | t(4) = 8.48 |  |
| P Value | p = 0.001 |  |
| Supplementary Fig. 5f | | |
| Test used | Two-way ANOVA; Bonferroni’s post hoc test | Three independent experiments |
| F (DFn, DFd) | F (1, 8) = 0.5402  F (1, 8) = 317.4  F (1, 8) = 0.5402 |  |
| P Value | p = 0.483  p < 0.001  p = 0.483 |  |
| Post hoc test | GFP-TDP-CTF^WT^: mCherry vs mCherry-KPNB1  GFP-TDP-CTF^Δ230-240^: mCherry vs mCherry-KPNB1 | p < 0.001  p < 0.001 |
| Supplementary Fig. 7 | | |
| Test used | One-way ANOVA; Bonferroni’s post hoc test | n=21-23 |
| F (DFn, DFd) | F (6, 143) = 177.4 |  |
| P Value | p < 0.001 |  |
| Post hoc test | GFP vs GFP-TDP-CTF  GFP vs GFP-TDP-43^mNLS^  GFP vs GFP-TDP-PrLD  GFP-TDP-CTF vs GFP-sTDP  GFP-TDP-43^mNLS^ vs GFP-sTDP | p < 0.001  p < 0.001  p < 0.001  p < 0.001  p < 0.001 |
| Supplementary Fig. 9b | | |
| Test used | Unpaired t test | n=21-22 |
| t(df) | t(41) = 14.5 |  |
| P Value | p < 0.001 |  |
| Supplementary Fig. 9f | | |
| Test used | Unpaired t test | Three independent experiments |
| t(df) | t(4) = 3.72 |  |
| P Value | p = 0.021 |  |
| Supplementary Fig. 9h | | |
| Test used | Two-way ANOVA; Bonferroni’s post hoc test | Four independent experiments |
| F (DFn, DFd) | F (1, 12) = 23.71  F (1, 12) = 213.7  F (1, 12) = 23.71 |  |
| P Value | p < 0.001  p < 0.001  p < 0.001 |  |
| Post hoc test | GFP-TDP-CTF^WT^: mCherry vs mCherry-KPNB1  GFP-TDP-CTF^VLIM-F^: mCherry vs mCherry-KPNB1  mCherry-KPNB1: GFP-TDP-CTF^WT^ vs GFP-TDP-CTF^VLIM-F^ | p < 0.001  p < 0.001  p < 0.001 |
| Supplementary Fig. 13 | | |
| Test used | One-way ANOVA; Sidak’s post hoc test | Two independent experiments |
| F (DFn, DFd) | F (2, 6) = 50.16 |  |
| P Value | p = 0.0002 |  |
| Post hoc test | LacZ vs V5-Ketel | p = 0.4845 |
| Supplementary Fig. 15c | | |
| Test used | OASIS tool; Fisher’s exact test | n=100 |
|  | RU486, TDP-43^M337V^: LacZ vs EP-Ketel | p < 0.0001 |
| Supplementary Fig. 16b | | |
| Test used | One-way ANOVA; Bonferroni’s post hoc test | n=5 |
| F (DFn, DFd) | F (2, 12) = 366 |  |
| P Value | p < 0.001 |  |
| Post hoc test | LacZ vs V5-Ketel  LacZ vs Ketel RNAi | p < 0.001  p < 0.001 |
| Supplementary Fig. 17b | | |
| Test used | One-way ANOVA; Bonferroni’s post hoc test | n=16-23 |
| F (DFn, DFd) | F (4, 91) = 118 |  |
| P Value | p < 0.001 |  |
| Post hoc test | Ctrl vs Ketel OE  Ctrl vs cdm OE  Ctrl vs cdm RNAi | p < 0.001  p < 0.001  p = 0.021 |
| Supplementary Fig. 18a | | |
| Test used | One-way ANOVA; Bonferroni’s post hoc test | Two independent experiments, eight technical replicates per experiment  n=52-264 |
| F (DFn, DFd) | F (7, 1261) = 47.82 |  |
| P Value | p < 0.001 |  |
| Post hoc test | GFP: mScarlet vs mScarlet-KPNB1  TDP-43^WT^: mScarlet vs mScarlet-KPNB1  TDP-43^mNLS^: mScarlet vs mScarlet-KPNB1  TDP-CTF: mScarlet vs mScarlet-KPNB1 | p > 0.999  p < 0.001  p = 0.599  p > 0.999 |
| Supplementary Fig. 18b | | |
| Test used | One-way ANOVA; Bonferroni’s post hoc test | Two independent experiments, eight technical replicates per experiment  n=52-264 |
| F (DFn, DFd) | F (7, 1188) = 80.57 |  |
| P Value | p < 0.001 |  |
| Post hoc test | GFP: mScarlet vs mScarlet-KPNB1  TDP-43^WT^: mScarlet vs mScarlet-KPNB1  TDP-43^mNLS^: mScarlet vs mScarlet-KPNB1  TDP-CTF: mScarlet vs mScarlet-KPNB1 | p > 0.999  p < 0.001  p < 0.001  p < 0.001 |
| Supplementary Fig. 19b | | |
| Test used | Two-way ANOVA; Bonferroni’s post hoc test | Three independent experiments |
| n | GFP  FLAG-KPNB1 H1-9^WT^  FLAG-KPNB1 H1-9^mNIS^  FLAG-KPNB1 H1-8^WT^  FLAG-KPNB1 H1-8^mNIS^  FLAG-KPNB1 H10-19 | n=168  n=178  n=158  n=162  n=160  n=161 |
| F (DFn, DFd) | F (10, 36) = 41.48  F (5, 36) = 1.775e-008  F (2, 36) = 22.51 |  |
| P Value | p < 0.001  p > 0.999  p < 0.001 |  |
| Post hoc test: cytoplasmic  mScarlet-TDP-CTF | GFP vs FLAG-KPNB1 H1-9^WT^  GFP vs FLAG-KPNB1 H1-9^mNIS^  GFP vs FLAG-KPNB1 H1-8^WT^  FLAG-KPNB1 H1-9^WT^ vs FLAG-KPNB1 H10-19  FLAG-KPNB1 H1-9^mNIS^ vs FLAG-KPNB1 H1-8^mNIS^  FLAG-KPNB1 H1-8^WT^ vs FLAG-KPNB1 H1-8^mNIS^ | p < 0.001  p < 0.001  p < 0.001  p < 0.001  p < 0.001  p < 0.001 |
| Post hoc test: nucleocytoplasmic  mScarlet-TDP-CTF | GFP vs FLAG-KPNB1 H1-9^mNIS^  GFP vs FLAG-KPNB1 H1-8^WT^  FLAG-KPNB1 H1-9^WT^ vs FLAG-KPNB1 H1-9^mNIS^  FLAG-KPNB1 H1-9^WT^ vs FLAG-KPNB1 H1-8^WT^  FLAG-KPNB1 H1-9^mNIS^ vs FLAG-KPNB1 H1-8^mNIS^  FLAG-KPNB1 H1-8^WT^ vs FLAG-KPNB1 H1-8^mNIS^ | p < 0.001  p = 0.001  p = 0.002  p = 0.03  p < 0.001  p = 0.006 |
| Post hoc test:  nuclear  mScarlet-TDP-CTF | GFP vs FLAG-KPNB1 H1-9^WT^  GFP vs FLAG-KPNB1 H1-8^WT^  FLAG-KPNB1 H1-9^WT^ vs FLAG-KPNB1 H1-9^mNIS^  FLAG-KPNB1 H1-9^WT^ vs FLAG-KPNB1 H1-8^WT^  FLAG-KPNB1 H1-9^WT^ vs FLAG-KPNB1 H10-19  FLAG-KPNB1 H1-8^WT^ vs FLAG-KPNB1 H1-8^mNIS^ | p < 0.001  p < 0.001  p < 0.001  p < 0.001  p < 0.001  p = 0.001 |
| Supplementary Fig. 21b | | |
| Test used | One-way ANOVA; Bonferroni’s post hoc test | n=21-29 |
| F (DFn, DFd) | F (3, 90) = 4.18 |  |
| P Value | p = 0.008 |  |
| Post hoc test | GFP vs GFP-KPNB1 FL  GFP vs GFP-KPNB1 H1-9  GFP vs GFP-KPNB1 H1-8 | p = 0.038  p = 0.128  p > 0.999 |

**Supplementary Table 3.** Summary of DNA constructs and sources.

| **Plasmids** | **Sources** |
| --- | --- |
| pEGFP-C1 / mCherry-C1 | Clontech |
| GFP-TDP-CTF/ mCherry-TDP-CTF | [1] |
| GFP-TDP-43^WT^ | [1] |
| GFP-TDP-43^mNLS^ (KRK…KVKR>AAA…AVAA) | [1] |
| GFP-sTDP | sTDP (gift from Sami Barmada [2]) was cloned into pEGFP-C1 |
| GFP-TDP-43^mNLS^ 1-265 | TDP-43^mNLS^ 1-265 fragment was cloned into pEGFP-C1 |
| GFP-TDP-CTF non-PrLD | TDP-CTF non-PrLD (amino-acids 208-274) was generated by PCR and cloned in pEGFP-C1 |
| GFP-TDP-43 PrLD | TDP-43 PrLD (amino-acids 275-414) was generated by PCR and cloned into pEGFP-C1 |
| GFP-TDP-CTF 229-414 → 260-414/ 208-259 → 208-219 | TDP-CTF deletion constructs were generated by PCR and cloned into pEGFP-C1 |
| GFP-TDP-CTF Q331K/ M337V/ A382T | ALS-causing mutations were amplified by PCR and cloned into GFP-TDP-CTF |
| GFP-TDP-CTF Δ274-313/ Δ314-353/ Δ354-393 | TDP-CTF PrLD deletion constructs (gift from Masato Hasegawa [3]) were cloned into GFP-TDP-CTF |
| GFP-TDP-CTF Δ230-240 | TDP-CTF lacking amino-acids 230-240 was generated by PCR and cloned into pEGFP-C1 |
| GFP-TDP-CTF F-A/ F-G / F-Y | Mutations were generated by site-directed mutagenesis and cloned into GFP-TDP-CTF |
| GFP-TDP-CTF G309F/ G368W/ 2xɸ/ 4xɸ/ K-R/ KRED-S/ FYW-L/ VLIM-F | Mutant constructs Addgene #107822/ #107837/ #107806/ #107805/ #118796/ #118795/ #118793/ #118794; gift from Rajat Rohatgi [4] were cloned into GFP-TDP-CTF |
| GFP-TDP-43^mNLS^ 5F-L | Gift from Chris Donnelly [5] |
| GFP-TDP-43^mNLS^ mNES | Mutations in the NES (I239A, L243A, L248A, I249A, I250A [6] were inserted in GFP-TDP-43^mNLS^ |
| GFP-TDP-43^mNLS^ G368W/ 4xɸ/ FYW-L/ VLIM-F | Mutant constructs (Addgene #107837/ #107805/ #118793/ #118794; gift from Rajat Rohatgi [4] were cloned into GFP-TDP-43^mNLS^ |
| GFP/ mCherry/ 3xFLAG-KPNB1 | mCherry and 3xFLAG were cloned into GFP-KPNB1 (Euroscarf # P30478; gift from Jan Ellenberg [7]) |
| Untagged KPNB1 | KPNB1 was amplified by PCR and cloned into pEGFP-C1 plasmid lacking the GFP tag |
| GFP-KPNB1 H1-9 → H1 | KPNB1 fragments were generated by PCR and cloned into pEGFP-C1 |
| GFP-KPNB1 H1-8^mNIS^ | NIS mutations (I178A, F217A, Y255A, I263R; described in [8] were generated by site-directed mutagenesis and cloned into pEGFP-C1 |
| GFP-KPNB1 H10-19 | KPNB1 H10-19 was generated by DNA synthesis and cloned into pEGFP-C1 |
| FLAG-KPNB1 H1-3 | KPNB1 H1-3 was generated by DNA synthesis and cloned into pEGFP-C1 |
| FLAG-KPNB1 H1-8^WT^/ H1-8^mNIS^ | 3xFLAG tag replaced GFP in GFP-KPNB1 H1-8^WT^/ H1-8^mNIS^ |
| mScarlet-C1 | mScarlet tag was cloned from mScarlet-I-LaminB (Addgene #98831) into pEGFP-C1 |
| V5-Ketel | Ketel coding sequence fused to a V5 N-terminal tag was cloned into pJFRC-MUH (Addgene #26213) |
| GFP-Ketel/ mScarlet-Ketel | Ketel was cloned into pEGFP- and mScarlet-C1 |
| mScarlet-Ketel H1-9/ Ketel H10-19 | Ketel fragments were generated by PCR and cloned into mScarlet-C1 |
| GFP-TNPO1 | Gift from James Shorter [9] |
| GFP-TNPO2A/ TNPO2B | TNPO2A and 2B constructs (gift from Joan Steitz [10]) were cloned into pEGFP-C1 |
| GFP-IPO4/ IPO7/ IPO8/ IPO9/ IPO11/ TNPO3/ IPO13 | Importin constructs (gift from Yuh Min Chook [11]) were cloned into pEGFP-C1 |
| GFP-IPO5 | Gift from Yi-Shuian Huang [12] |
| GFP-KPNA1 → KPNA7 | KPNA1 → KPNA7 constructs (Addgene #26677 → #26683; gift from Bryce Paschal [13]) were cloned into pEGFP-C1 |
| GFP-XPO1/ XPO2/ XPOT/ XPO6/ XPO7/ RANBP6/ RANBP17 | Exportin constructs (gift from Dirk Görlich) were cloned into pEGFP-C1 |
| GFP-XPO4 | XPO4 (gift from Yasushi Hiraoka [14]) was cloned into pEGFP-C1 |
| GFP-XPO5 | Addgene #58331; gift from Matthew Wood [15] |
| Nup62-mCherry | mCherry replaced GFP in pNup62-EGFP3 (Euroscarf #P30484; gift from Jan Ellenberg [7] |
| mCherry-Nup85 | mCherry replaced GFP in pEGFP3-Nup85 (Euroscarf #P30485; gift from Jan Ellenberg [7]) |
| GFP-Nup98 | Euroscarf #P30487; gift from Jan Ellenberg [7]) |
| NES-tdTomato-NLS | Gift from Martin Hetzer [16] |
| GFP-(GR)_100_ | A fragment with 100 GR repeats was generated by DNA synthesis and cloned into pEGFP-C1 |
| GFP-FUSΔ14 | FUS lacking the last 14 amino-acids was generated by DNA synthesis and cloned into pEGFP-C1 |
| AAV-Syn-GFP | Addgene #50465; gift from Bryan Roth |
| AAV-Syn-GFP-TDP-43^mNLS^ | GFP-TDP-43^mNLS^ was cloned into AAV-Syn-GFP |
| AAV-Syn-mScarlet-TDP-CTF | mScarlet-TDP-CTF was cloned into AAV-Syn-GFP |
| AAV-Syn-FLAG-mCherry | FLAG-mCherry was cloned into AAV-Syn-GFP |
| AAV-Syn-FLAG-KPNB1 H1-9^WT^/ H1-9^mNIS^/ H1-8^WT^/ H1-8^mNIS^/ H10-19 | FLAG-KPNB1 constructs were cloned into AAV-Syn-GFP |
| pGW1-GFP | GFP was cloned into pGW1 |
| pGW1-GFP-TDP-43 WT/ mNLS | TDP-43 WT/ mNLS were cloned into pGW1-GFP |
| pGW1-GFP-TDP-CTF | GFP-TDP-CTF was cloned into pGW1 |
| pGW1-mScarlet | mScarlet was cloned into pGW1 |
| pGW1-mScarlet-KPNB1 | KPNB1 was cloned into pGW1-mScarlet |
| pGW1-NLS-mTagBFP | NLS-mTagBFP was cloned into pGW1 |
| pGW1-HaloTag | HaloTag was cloned into pGW1 |
| His_6_-TDP-CTF | TDP-CTF was cloned into pET28a |
| His_6_-Nup62FG | His_6_-Nup62FG (amino-acids 1-268) was described previously [17]. |
| GST-KPNB1 WT/ mNIS/ H1-9^WT^/ H1-9^mNIS^ | KPNB1 constructs were cloned into pGEX-6P |

**Supplementary Table 4.** Summary of *Drosophila* strains and sources.

| **Drosophila strains** | **Sources** |
| --- | --- |
| UAS-hTDP-43, UAS-hTDP-43^M337V^ and UAS-TDP-43^ΔNLS^ | Gift from JP Taylor [18] |
| Gmr-Gal4 driver (1104) | Bloomington Drosophila Stock Center |
| elavGeneSwitch (GS)-Gal4 | “ |
| UAS-LacZ (8529 and 8530) | “ |
| P{EPgy2}Fs(2)Ket^EY06666^ (15967) | “ |
| UAS-Ketel RNAi (31242, 41845, 44576, and 27567) | “ |
| UAS-Ketel RNAi (107622) | Vienna Drosophila Resource Center |
| UAS-V5-Ketel | This study |
| cdm RNAi: y[1]v[1];P{y[+t7.7]v[+t1.8]=TRiP.JF01428}attP2 (31639) | Bloomington Drosophila Stock Center |
| cdm OE: y[1] w[*]; P{w[+mC]=UAS-imp13.G} (58711) | Bloomington Drosophila Stock Center |

**Supplementary Table 5.** Summary of reagents and sources.

| **Primary Antibodies** | **Sources** | **Catalog/lot number** |
| --- | --- | --- |
| Rabbit anti-TDP-43 | Proteintech | 12892-1-AP/00102386 |
| Mouse anti-phospho-TDP-43^S409/410^ | Cosmo | TIP-PTD-M01/11-9-20 |
| Rabbit anti-phospho-TDP-43^S409/410^ (for fly specimens) | Proteintech | 22309-1-AP |
| Rat anti-phospho-TDP-43^S409/410^ (for human tissue) | BioLegend | 829901/B352848 |
| Rabbit anti-KPNB1 (for western blot) | Novus Biologicals | NBP1-87712/00004091 |
| Mouse anti-KPNB1 (for immunocytochemistry) | Abcam | AB2811/GR3205596-11 |
| Mouse anti-KPNB1 (for human tissue staining) | Santa Cruz Biotechnology | SC-137016/G2621 |
| Mouse anti-nuclear pore complex proteins (mAb414) | BioLegend | 902901/B291104 |
| Rabbit anti-Nup50 | Abcam | AB137092/GR101996-8 |
| Mouse anti-Nup62 | BD labs | 610497/9107962 |
| Rabbit anti-Nup98 | Cell Signaling | 2598S/4 |
| Mouse anti-Ran | Sigma Aldrich | R4777 |
| Mouse anti-importin-α1 | Novus Biologicals | MAB6207 |
| Mouse anti-FLAG (M2) | Sigma Aldrich | F1804 |
| Mouse anti-β-tubulin | DSHB | E7-s |
| Mouse anti-β-actin | Sigma Aldrich | A2228 |
| Mouse anti-mCherry | Novus Biologicals | NBP1-96752/092421 |
| Chicken anti-GFP | Aves Labs | GFP-1020/GFP3717982 |

| **Secondary Antibodies and Fluorescent Dyes** | **Sources** | **Catalog/lot number** |
| --- | --- | --- |
| Anti-Mouse IgG, Alexa Fluor Plus 555 conjugated | Invitrogen | A32727/VC295499 |
| Anti-Mouse IgG, Alexa Fluor Plus 647 conjugated | Invitrogen | A32728/WK331591 |
| F’(ab) anti-Mouse IgG, Alexa Fluor Plus 488 conjugated | Invitrogen | A48286TR/WB322583 |
| Anti-Rabbit IgG, Alexa Fluor Plus 488 conjugated | Invitrogen | A32731/SC243838 |
| Anti-Rabbit IgG, Alexa Fluor Plus 555 conjugated | Invitrogen | A32732/UC277969 |
| Anti-Rabbit IgG, Alexa Fluor 647 Plus conjugated | Invitrogen | A32733/WH308578A |
| Anti-Rat IgG, Alexa Fluor 555 conjugated | Invitrogen | A21434/2184321 |
| Anti-Rat IgG, Alexa Fluor Plus 647 conjugated | Invitrogen | A48265TR/XA345979 |
| Anti-Chicken IgG, Alexa Fluor 488 conjugated | Invitrogen | A11039/2180688 |
| Anti-Mouse IgG, Alexa Fluor Plus 680 | Invitrogen | A32729/UK287771 |
| Anti-Mouse IgG, Alexa Fluor Plus 800 | Invitrogen | A32730/VL313935 |
| Anti-Rabbit IgG, Alexa Fluor Plus 680 | Invitrogen | A32734/VI308536 |
| Anti-Rabbit IgG, Alexa Fluor Plus 800 | Invitrogen | A32735/TG267417 |
| Anti-Chicken IgG, IRDye 680RD | LI-COR | 926-68075/C80717-13 |
| Anti-Chicken IgG, IRDye 800CW | LI-COR | 926-32218/C81002-07 |
| Live-or-Dye™ 640/662 Fixable Viability Staining Kit | Biotium | 32007 |
| Amylo-Glo RTD Amyloid Plaque Stain Reagent | Biosensis | TR-300-AG |
| Hoechst 33342 | Thermo Fisher Scientific | 62249/UD2750232 |
| DRAQ5 Fluorescent Probe | Thermo Fisher Scientific | 62251/528DR50200 |

| **Chemicals and reagents** | **Sources** | **Catalog/lot number** |
| --- | --- | --- |
| PolyMag Neo | OZ Biosciences | PG61000 |
| NeuroMag | OZ Biosciences | NM51000 |
| Protease Inhibitor Tablets, EDTA free | Thermo Fisher Scientific | A32965/VL3120614 |
| ProLong® Glass Antifade Mountant | Invitrogen | P36980/2342881 |
| GFP-Trap magnetic agarose beads | Chromotek | gtma-100 |
| RFP-Trap magnetic agarose beads | Chromotek | rtma-20 |
| Dako Antibody Diluent | Agilent | S0809/11213330 |
| Dako Target Retrieval Solution | Agilent | S2369/41362183 |
| True Black Lipofuscin Autofluorescence Quencher | Biotium | 23007/21T0325 |
| RU486 | Cayman Chemical | 10006317 |
| Polyethylenimine Hydrochloride (PEI) | Polysciences | 24765-1 |

**References**

1. Chou CC, Zhang Y, Umoh ME, Vaughan SW, Lorenzini I, Liu F, et al. TDP-43 pathology disrupts nuclear pore complexes and nucleocytoplasmic transport in ALS/FTD**.** *Nat Neurosci* 2018;21**:**228-239.

2. Weskamp K, Tank EM, Miguez R, McBride JP, Gomez NB, White M, et al. Shortened TDP43 isoforms upregulated by neuronal hyperactivity drive TDP43 pathology in ALS**.** *J Clin Invest* 2020;130**:**1139-1155.

3. Shimonaka S, Nonaka T, Suzuki G, Hisanaga S, Hasegawa M. Templated Aggregation of TAR DNA-binding Protein of 43 kDa (TDP-43) by Seeding with TDP-43 Peptide Fibrils**.** *J Biol Chem* 2016;291**:**8896-8907.

4. Schmidt HB, Barreau A, Rohatgi R. Phase separation-deficient TDP43 remains functional in splicing**.** *Nat Commun* 2019;10**:**4890.

5. Mann JR, Gleixner AM, Mauna JC, Gomes E, DeChellis-Marks MR, Needham PG, et al. RNA Binding Antagonizes Neurotoxic Phase Transitions of TDP-43**.** *Neuron* 2019;102**:**321-338 e328.

6. Winton MJ, Igaz LM, Wong MM, Kwong LK, Trojanowski JQ, Lee VM. Disturbance of nuclear and cytoplasmic TAR DNA-binding protein (TDP-43) induces disease-like redistribution, sequestration, and aggregate formation**.** *J Biol Chem* 2008;283**:**13302-13309.

7. Rabut G, Doye V, Ellenberg J. Mapping the dynamic organization of the nuclear pore complex inside single living cells**.** *Nat Cell Biol* 2004;6**:**1114-1121.

8. Bednenko J, Cingolani G, Gerace L. Importin beta contains a COOH-terminal nucleoporin binding region important for nuclear transport**.** *J Cell Biol* 2003;162**:**391-401.

9. Guo L, Kim HJ, Wang H, Monaghan J, Freyermuth F, Sung JC, et al. Nuclear-Import Receptors Reverse Aberrant Phase Transitions of RNA-Binding Proteins with Prion-like Domains**.** *Cell* 2018;173**:**677-692 e620.

10. Rebane A, Aab A, Steitz JA. Transportins 1 and 2 are redundant nuclear import factors for hnRNP A1 and HuR**.** *RNA* 2004;10**:**590-599.

11. Soniat M, Cagatay T, Chook YM. Recognition Elements in the Histone H3 and H4 Tails for Seven Different Importins**.** *J Biol Chem* 2016;291**:**21171-21183.

12. Chao HW, Lai YT, Lu YL, Lin CL, Mai W, Huang YS. NMDAR signaling facilitates the IPO5-mediated nuclear import of CPEB3**.** *Nucleic Acids Res* 2012;40**:**8484-8498.

13. Kelley JB, Talley AM, Spencer A, Gioeli D, Paschal BM. Karyopherin alpha7 (KPNA7), a divergent member of the importin alpha family of nuclear import receptors**.** *BMC Cell Biol* 2010;11**:**63.

14. Tsuchiya M, Ogawa H, Suzuki T, Sugiyama N, Haraguchi T, Hiraoka Y. Exportin 4 interacts with Sox9 through the HMG Box and inhibits the DNA binding of Sox9**.** *PLoS One* 2011;6**:**e25694.

15. Seow Y, Sibley CR, Wood MJ. Artificial mirtron-mediated gene knockdown: functional DMPK silencing in mammalian cells**.** *RNA* 2012;18**:**1328-1337.

16. Hatch EM, Fischer AH, Deerinck TJ, Hetzer MW. Catastrophic nuclear envelope collapse in cancer cell micronuclei**.** *Cell* 2013;154**:**47-60.

17. Konishi HA, Yoshimura SH. Interactions between non-structured domains of FG- and non-FG-nucleoporins coordinate the ordered assembly of the nuclear pore complex in mitosis**.** *FASEB J* 2020;34**:**1532-1545.

18. Ritson GP, Custer SK, Freibaum BD, Guinto JB, Geffel D, Moore J, et al. TDP-43 mediates degeneration in a novel Drosophila model of disease caused by mutations in VCP/p97**.** *J Neurosci* 2010;30**:**7729-7739.
